# Comparative Efficacy and Acceptability of Noninvasive Brain Stimulation for Migraine: A Systematic Review and Network Meta‐Analysis of Randomized Controlled Trials

**DOI:** 10.1002/brb3.71566

**Published:** 2026-07-19

**Authors:** Wen‐Ting Sun, Jiakun Liu, Yao‐Hao Jiang, Ying‐Kun Zhao, Yi‐Cai, Yi‐Feng Wu, Tian‐li Li, Pei‐Feng Chang

**Affiliations:** ^1^ Department of Cardiovascular Medicine Dongzhimen Hospital Beijing China; ^2^ Department of Integrated Traditional Chinese and Western Medicine Cardiology China–Japan Friendship Hospital Beijing China; ^3^ Beijing University of Chinese Medicine Beijing China

**Keywords:** migraine, network meta‐analysis, noninvasive brain stimulation, randomized controlled trial, transcranial direct current stimulation

## Abstract

**Objective:**

This network meta‐analysis (NMA) aimed to compare and rank the efficacy and acceptability of different noninvasive brain stimulation (NIBS) techniques for migraine treatment in adults.

**Method:**

We systematically searched PubMed, Web of Science, Embase, Cochrane Library, and ClinicalTrials.gov through October 13, 2025, for randomized controlled trials (RCTs) in adults with migraine. The primary outcomes were changes in monthly migraine days (MMD) and dropout rates, analyzed using a random‐effects model. Heterogeneity was assessed with *I* ^2^, risk of bias (RoB) with the Cochrane RoB 2 tool, and evidence quality with Grading of Recommendations Assessment, Development, and Evaluation (GRADE) criteria.

**Result:**

This NMA integrated data from 28 RCTs encompassing 1920 participants. Among the evaluated interventions, visual cortex (V1) transcranial direct current stimulation (tDCS) over Oz–Cz (VC‐tDCS‐OzCz)—an anodal protocol targeting the V1—ranked highest for reducing MMD (mean difference [MD] = −1.75; 95% CI: −3.37 to −0.13; surface under the cumulative ranking curve [SUCRA]: 83.5%). For acceptability, only transcutaneous occipital nerve stimulation (tONS) significantly reduced dropout rates versus sham (risk ratio [RR] = 0.36, 95% CI: 0.16–0.82).

**Conclusion:**

Among the interventions evaluated, VC‐tDCS‐OzCz showed the largest reduction in MMD. However, these findings are based on predominantly moderate‐to‐low certainty evidence (GRADE), substantial heterogeneity, and a sparse network; they require confirmation in adequately powered, protocol‐standardized trials.

## Graphical abstract

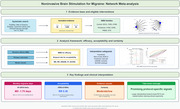

**Abbreviations**
95% CI: 95% confidence interval
DLPFC: dorsolateral prefrontal cortex
GRADE: Grading of Recommendations Assessment, Development, and Evaluation
ICHD: International Classification of Headaches Disorders
M1: primary motor cortex
MD: mean difference
NIBS: noninvasive brain stimulation
NMA: network meta‐analysis
Nvns: noninvasive vagus nerve stimulation
PICOS: population, intervention, comparison, outcomes, and study design
PMES: percutaneous mastoid electrical stimulation
RCT: randomized controlled trial
RR: risk ratio
STS: supraorbital transcutaneous stimulation
SUCRA: surface under the cumulative ranking curve
taVNS: transcutaneous auricular vagus nerve stimulation
tDCS: transcranial direct current stimulation
tONS: transcutaneous occipital nerve stimulation
VC: visual cortex
VAS: visual analog scale

## 1 Background

Migraine is a primary neurovascular disorder characterized by recurrent episodes of unilateral, throbbing, moderate‐to‐severe headache. These episodes are often accompanied by a spectrum of symptoms, ranging from nausea, vomiting, photophobia, and phonophobia to more complex neurological disturbances such as unilateral sensory abnormalities or olfactory changes. The global impact of migraine is substantial; it ranks as the second leading cause of disability worldwide and accounts for 16.3% of all disability stemming from neurological conditions (Amiri et al. 2022). While its precise pathophysiology is not fully elucidated, it is understood to involve both the central and peripheral nervous systems (IHS 2018). Proposed key mechanisms include peripheral and central sensitization, impaired habituation, thalamocortical dysrhythmia, and cortical hyperexcitability (Goadsby et al. 2017).

While recent advances in preventive therapy have targeted the calcitonin gene‐related peptide (CGRP) pathway with drugs like monoclonal antibodies and oral gepants (Cohen et al. 2022), their clinical utility is constrained by significant limitations. These therapies act primarily in the periphery, limiting their efficacy in patients with centrally driven mechanisms and often resulting in low response rates or residual headaches (Edvinsson et al. 2018). Furthermore, adverse reactions and tolerability issues contribute to poor patient compliance (Whyte and Tepper 2009). Compounding these challenges, the risk of medication overuse headache (MOH) from acute‐phase treatments complicates clinical management.

In this context, neuromodulation—encompassing noninvasive, minimally invasive, and surgical approaches—has emerged as a valuable alternative. Noninvasive strategies, in particular, are gaining attention as a promising nonpharmacological option for migraine prevention (Puledda et al. 2017). Although the certainty of existing evidence is limited by study heterogeneity, this approach offers a vital avenue for patients who prefer nonpharmacological treatments or have had a suboptimal response to medication. Noninvasive brain stimulation (NIBS) technologies are broadly classified into two main categories: central stimulation, which directly targets the cerebral cortex (e.g., transcranial direct current stimulation [tDCS] and repetitive transcranial magnetic stimulation [rTMS]) and peripheral stimulation, which targets peripheral nerves (e.g., noninvasive vagus nerve stimulation [nVNS], transcutaneous electrical nerve stimulation [TENS], transcutaneous occipital nerve stimulation [tONS], and supraorbital transcutaneous stimulation [STS]). tDCS and rTMS, two primary NIBS techniques, operate via distinct mechanisms. tDCS modulates cortical excitability by delivering a weak (1–2 mA) constant direct current through scalp electrodes, inducing long‐term potentiation (LTP)–like changes in synaptic plasticity (Lefaucheur et al. 2017). Conversely, rTMS utilizes transient, high‐intensity magnetic pulses to transcranially induce electrical currents, which cause local neuronal depolarization and thereby alter cortical excitability.

While mounting evidence indicates that NIBS is a safe, well‐tolerated, and effective approach for migraine prevention, the optimal protocol remains unclear. Existing studies have explored various stimulation targets and parameters, often yielding conflicting results, and direct head‐to‐head comparisons between techniques are scarce. Consequently, a comprehensive, quantitative review is urgently needed to evaluate the relative efficacy of these different NIBS protocols. Although the network meta‐analysis (NMA) by Cheng et al. (2022) provided preliminary evidence in this field, the study had certain limitations. Its literature search was limited to June 2021, omitting several important randomized controlled trials (RCTs) published thereafter. Additionally, the assessed outcome measures were relatively limited. Therefore, this study aims to conduct an updated and more comprehensive NMA. By incorporating the latest RCTs and expanding the assessment to include outcomes such as quality of life, we aim to provide a more robust evidence base for clinical decision‐making and guideline updates regarding NIBS in migraine treatment.

## 2 Method

### 2.1 Search Strategy and Study Registration

A systematic search was conducted across multiple databases, including PubMed, EMBASE, Web of Science, and the Cochrane Library. Gray literature was searched on ClinicalTrials.gov. The search spanned from the inception of each database to October 13, 2025. Specific search terms are documented in Table S2. To minimize bias, we also manually searched review articles and pairwise meta‐analyses for potentially eligible studies. The initial search strategy employed a combination of MeSH terms, free‐text keywords, and filters for migraine and headache, as well as for noninvasive electrical stimulation, combined using Boolean operators (AND/OR). This NMA was reported in accordance with the Preferred Reporting Items for Systematic Reviews and Meta‐Analyses (PRISMA‐NMA) statement and was registered in the PROSPERO database (anonymized).

### 2.2 Inclusion and Exclusion Criteria

The inclusion criteria were defined using the PICOS (population, intervention, comparison, outcomes, and study design) framework as follows: Population (P): Adult patients (aged ≥ 18 years) diagnosed with migraine according to the criteria of the International Classification of Headache Disorders (ICHD‐II or ICHD‐III beta version). Eligible populations included episodic migraine (EM), chronic migraine (CM), or a mix of both, with or without aura. No restrictions were placed on gender or ethnicity. Intervention (I): Any NIBS technique. Comparison (C): Studies comparing NIBS with sham stimulation, as well as studies directly comparing different NIBS protocols. Outcomes (O): Studies were eligible if they reported ≥ 1 of the following outcomes, all prespecified in the PROSPERO protocol (anonymized) before literature screening: Primary: (1) change in monthly migraine days (MMD) and (2) acceptability (all‐cause dropout rate). Secondary: (1) pain intensity (visual analog scale [VAS]: 0–10), (2) attack frequency, (3) attack duration, (4) analgesic use, (5) Headache Impact Test‐6 (HIT‐6) score, (6) responder rate (≥ 50% reduction in migraine frequency), and (7) adverse events (AEs) (≥ 1 event). Studies reporting only other outcomes were excluded. When multiple time points were available, the rule in data analysis was applied. Eligible outcomes under this protocol may differ from the original trials' primary end points; studies qualified regardless of the end point's designation in the original publication. Study Design (S): RCTs.

Studies were excluded if they met any of the following criteria:

1. Populations that included patients with an unconfirmed migraine diagnosis or mixed headache types (e.g., tension‐type headache, secondary headaches).
2. Insufficient data reporting that prevented quantitative analysis or data extraction. Nonrandomized study designs.
3. Presence of confounding co‐interventions (e.g., concurrent initiation of new medications or therapies in one arm only).
4. Publication types other than full‐text articles, such as conference abstracts, commentaries, case reports, guidelines, or duplicate publications.
5. Non‐English language publications.

### 2.3 Primary and Secondary Outcomes

The primary outcome measures were (1) change in MMD and (2) acceptability (dropout rate), defined as the proportion of participants who withdrew from the study for any reason before its completion. Secondary outcome measures included (1) change in migraine severity, assessed using a VAS for pain intensity, where 0 represents no pain and 10 represents the most severe pain; (2) attack frequency; (3) duration; (4) analgesic medication usage; (5) HIT‐6 score; (6) responder rate, defined as ≥ 50% reduction in baseline migraine frequency after intervention; and (7) AEs, defined as the proportion of patients reporting at least one AE. All outcomes relied on patient self‐reporting and were primarily recorded in headache diaries.

### 2.4 Data Extraction

Literature screening, data extraction, and risk of bias (RoB) assessment were independently performed by two reviewers (W. T. S. and Y. H. J.). Any disagreements were resolved by consensus or through adjudication by a third reviewer (P. F. C.). Data extraction was performed using a predesigned Excel template. Extracted data included study information (first author, publication year, country), baseline characteristics of the study population (sex distribution, age, sample size), diagnostic criteria, migraine type, treatment and follow‐up duration, stimulation parameters (e.g., current intensity, frequency), intervention details (device model, stimulation site, montage configuration, reference electrode, electrode size, stimulation duration), and primary outcome measures. All data were obtained directly from the published literature; no additional data were requested from the original authors.

### 2.5 Definition of Nodes and Treatment Protocols

In this NMA, each intervention is defined as a unique combination of the following three core elements: (1) stimulation modality: the specific type of NIBS (e.g., tDCS, rTMS); (2) stimulation target: the specific cortical region (e.g., primary motor cortex [M1], dorsolateral prefrontal cortex [DLPFC], visual cortex [VC]); and (3) key distinguishing parameter: for tDCS, primarily polarity (anode vs. cathode) and for rTMS, primarily frequency (e.g., high‐frequency rTMS ≥ 5 Hz, low‐frequency rTMS ≤ 1 Hz).

### 2.6 RoB Assessment

Two reviewers (W. T. S. and Y. H. J.) independently assessed the RoB for the primary outcome in each included RCT using the Cochrane RoB tool version 2.0 (RoB 2.0). The assessment covered the following five domains:

1. Bias arising from the randomization process.
2. Bias due to deviations from intended interventions.
3. Bias due to missing outcome data.
4. Bias in measurement of the outcome.
5. Bias in selection of the reported result.

The RoB for each domain was judged as “low risk,” “some concerns,” or “high risk,” leading to an overall RoB judgment for each study. Any disagreements between the reviewers were resolved by consensus or, if necessary, through adjudication by a senior researcher (T. L. L.). Given the inherent challenges of blinding in neurostimulation trials, a lack of participant or personnel blinding did not, in itself, result in a “high risk” judgment for the relevant domains.

### 2.7 Quality Assessment

We assessed the certainty of the evidence for each outcome using the GRADE (Grading of Recommendations, Assessment, Development, and Evaluations) approach. The certainty was evaluated across five domains: RoB, inconsistency, indirectness, imprecision, and publication bias. Each outcome was rated as having one of four levels of certainty: high, moderate, low, or very low. The assessment was performed independently by two reviewers (W. T. S. and Y. K. Z) using the GRADEpro software. Any disagreements were resolved through discussion to reach consensus or, if needed, by consulting a third reviewer. As all included studies were RCTs, the initial certainty of evidence for each outcome started at “high.” The final certainty level could be downgraded based on concerns within the five domains mentioned above.

### 2.8 Assessment of Transitivity

Transitivity is a core assumption of NMA. We assessed the distribution of potential effect modifiers—migraine subtype, treatment duration, follow‐up length, stimulation parameters, and RoB—across treatment nodes. A node‐level summary is provided in Table S4.

### 2.9 Data Analysis

This NMA was conducted within a frequentist framework using STATA 17.0 software. First, we constructed an evidence network diagram, which revealed that all interventions formed a single connected network with sham stimulation as the central node, confirming the feasibility of performing an NMA. Given the heterogeneity among studies and the limited number of studies included in the analysis, a random‐effects model was used for all analyses to obtain more robust and conservative effect estimates. For continuous outcomes, the effect size was the mean difference (MD) or Hedges' *g* for standardized MD. For dichotomous outcomes—including dropout rates, responder rates (≥ 50% reduction in migraine frequency from baseline), and AEs—risk ratios (RRs) with 95% confidence intervals (CI) were used. RR was chosen over OR because it is more directly interpretable for clinicians and is the recommended effect measure for prospective study designs such as RCTs. When a study reported outcomes at multiple time points, data were extracted at the primary end point of the original trial. If the primary end point was not specified, the time point closest to the end of treatment was used. If only posttreatment follow‐up was available, the earliest such time point was selected. Detailed treatment and follow‐up time points are provided in Table 1 and summarized at the node level in Table S4. All effect sizes are presented with their 95% CI. In cases where a study arm for a dichotomous outcome had zero events, a continuity correction of 0.5 was applied. All comparisons were two‐sided tests, with *p* < 0.05 considered statistically significant. The statistical heterogeneity for each pairwise comparison was quantified using the *I* ^2^ statistic, where a value ≥ 50% was considered to indicate substantial heterogeneity. To quantify the range of treatment effects expected in future studies, 95% prediction intervals were calculated for key intervention‐versus‐sham/control comparisons summarized in Table S11. All analyses were conducted under the core assumptions of NMA: homogeneity, transitivity, and consistency. First, we performed standard pairwise meta‐analyses for all direct comparisons available in the network. Subsequently, indirect and mixed‐treatment effects were estimated using a random‐effects model. To assess the consistency assumption, we used both local and global approaches. The node‐splitting method was used to detect local inconsistency between direct and indirect evidence for specific comparisons. A design‐by‐treatment interaction model was employed to evaluate global inconsistency across the entire network. These tests evaluate the consistency assumption rather than confirm it; a nonsignificant result does not establish consistency, particularly in sparse networks where statistical power is limited (Higgins et al. 2014). We used the surface under the cumulative ranking curve (SUCRA) values to rank the interventions. A higher SUCRA value indicates a greater likelihood of an intervention being among the most effective options (Salanti et al. 2011). Finally, potential publication bias was evaluated by examining comparison‐adjusted funnel plots and using Egger's test where appropriate. Finally, to assess the robustness of the results, we conducted a sensitivity analysis by excluding studies with lower methodological quality or greater heterogeneity to observe changes in the pooled effect size.

**TABLE 1:** Characteristics of the included studies.

Study	Country	Diagnostic criteria	Sex ratio	Age (mean ± SD)	Duration (week)	Follow‐up duration (week)	Migraine type	Active stimulation	Participants	AE	Main outcome
Pohl et al. (2021)	Switzerland	ICHD‐III	EG: 12 F/0 M; CG: 10 F/1 M	EG = 41 ± 15; CG = 34 ± 10	4	16	Episodic migraine	VC‐tDCS‐OzCz	EG = 11; CG = 12	EG: 21 AEs; CG: 21 AEs	Migraine days
Hodaj et al. (2022)	France	ICHD‐III	EG: 10 F/8 M; CG: 16 F/2 M	EG = 54.5 ± 10.6; CG = 46.1 ± 14.1	8	12	Chronic migraine	a‐tDCS‐C3+c‐tDCS‐Fp2	EG = 14; CG = 14	No	Headache days
Dalla Volta et al. (2020)	Italy	ICHD‐III	Total: 30 F/15 M	45 ± 3.7	5 days	16	Chronic migraine	c‐tDCS‐FC	EG = 28; CG = 17	No	Headache days
DaSilva et al. (2023)	USA	ICHD‐III	EG: 2 F/11 M; CG: 1 F/11 M	EG = 30.1 ± 13.8; CG = 30.3 ± 8.8	2	4	Episodic migraine with or without aura	HD‐tDCS‐M1	EG = 13; CG = 12	No	Headache days, responder rate
Rahimi et al. (2020)	Iran	ICHD‐III beta	M1: 12 F/3 M; S1: 14 F/1 M; sham: 14 F/1 M	M1 = 36.40 ± 13.18; S1 = 34.06 ± 11.51; sham = 33.66 ± 11.28	10	48	Episodic migraine with or without aura	c‐M1‐tDCS‐C4; c‐S1‐tDCS‐CP4	M1 = 15; S1 = 15; sham = 15	No	Headache days, migraine frequency, headache intensity
Antal et al. (2011)	Germany	ICHD‐II	EG: 12 F/1 M; CG: 11 F/2 M	EG = 33.2 ± 10.4; CG = 32.3 ± 12.3	6	8	Episodic (with or without aura)	c‐tDCS‐Oz+a‐tDCS‐Cz	EG = 12; CG = 14	No	Migraine frequency
Rocha et al. (2015)	Germany	ICHD‐II	EG: 9 F/1 M; CG: 5 F/10 M	EG = 22 ± 4; CG = 28 ± 14	4	4	Episodic migraine	c‐tDCS‐Oz+a‐tDCS‐Cz	EG = 10; CG = 5	No	Headache intensity
Andrade et al. (2017)	Brazil	ICHD‐III	EG: 5 F/5 M; CG: 2 F/1 M	M1 = 31.5 ± 13.9; DLPFC = 32.3 ± 14.0; sham = 34.5 ± 13.4	4	NA	Refractory chronic migraine	a‐tDCS‐M1/DLPFC‐C3	EG = 9; CG = 4	EG: 23 AEs; CG: 6 AEs	Quality of life
Grazzi et al. (2020)	Italy	ICHD‐III	EG: 77 F/12 M; CG: 37 F/9 M	Anodal = 47.8 ± 10.8; cathodal = 47.7 ± 13.1; sham = 45.5 ± 11.3	5	48	Chronic migraine with medication overuse	a/c‐tDCS‐C4	EG = 99; CG = 46	No	Migraine days
Şirin et al. (2021)	Turkey	ICHD‐III	EG: 28 F/8 M; CG: 35 F/6 M	EG = 42.8 ± 8.84	3 days	4	Both episodic and chronic migraine	a‐tDCS‐M1‐C3	EG = 36; CG = 41	EG: 5 AEs; CG: 0 AEs	Headache days
Auvichayapat et al. (2012)	Thailand	ICHD‐II	EG: 14 F/6 M; CG: 12 F/5 M	EG = 28.60 ± 6.83; CG = 35.0 ± 13.54	20 days	12	Both episodic and chronic migraine	a‐tDCS‐M1‐C3	EG = 20; CG = 17	No	Headache frequency
Aksu et al. (2023)	Turkey	ICHD‐III	EG: 11 F/0 M; CG: 12 F/0 M	EG = 36.00 ± 12.29; CG = 43.08 ± 10.30	4	12	Migraine and allodynia	a‐tDCS‐M1‐C3	EG = 11; CG = 12	No	Allodynia levels and responder rates
Teepker et al. (2010)	Germany	ICHD‐II	EG: 13 F/1 M; CG: 9 F/4 M	EG = 30.71 ± 8.94; CG = 40.62 ± 11.53	5 days	8	Chronic migraine	Lf‐rTMS‐Cz	EG = 14; CG = 13	EG: 7 AEs; CG: 8 AEs	Migraine frequency
Granato et al. (2019)	Italy	ICHD‐III	NA	EG = 46.1 ± 7.14; CG = 45.4 ± 12.7	2	12	Chronic migraine and medication overuse headache	Hf‐rTMS‐LDLPFC	EG = 7; CG = 7	EG: 2 AEs; CG: 0 AEs	Headache days
Amin et al. (2020)	Egypt	ICHD‐III	EG: 13 F/1 M; CG: 15 F/4 M	EG = 37.4 ± 11.7; CG = 32.2 ± 9.8	1	4	Episodic migraine	Hf‐rTMS‐LDLPFC	EG = 13; CG = 16	No	Migraine frequency
Misra et al. (2013)	India	ICHD‐II	EG: 44 F/6 M; CG: 44 F/6 M	EG = 35.62 ± 10.07; CG = 35.06 ± 10.38	4	4	Chronic migraine	Hf‐rTMS‐LDLPFC	EG = 47; CG = 48	No	Headache frequency, > 50% improvement on a 0–100 VAS
Song et al. (2025)	China	ICHD‐III	EG: 9 F/5 M; CG: 8 F/6 M	EG = 36.4 ± 7.1; CG = 38.5 ± 5.6	2	4	Migraine with or without aura	Hf‐rTMS‐LDLPFC	EG = 14; CG = 14	No	Migraine days and headache severity on the VAS
Conforto et al. (2014)	Brazil	ICHD‐II	EG: 7 F/0 M; CG: 7 F/0 M	EG = 41.4 ± 12.5; CG = 36.2 ± 11.3	8	NR	Chronic migraine	Hf‐rTMS‐LDLPFC	EG = 7; CG = 7	No	Intervention adherence, headache days, adverse event incidence
Lipton et al. (2010)	USA	ICHD‐II	EG: 67 F/15 M; CG: 63 F/19 M	EG = 38.8 ± 11.2; CG = 40.1 ± 10.8	Single acute treatment, up to 3 attacks	2 h; sustained response at 24/48 h	Migraine with aura	sTMS	EG = 82; CG = 82	No	Pain‐free response 2 h
Najib et al. (2022)	USA	ICHD‐III	EG: 49 F/7 M; CG: 44 F/13 M	EG = 40.3 ± 13.9; CG = 44.6 ± 10.7	12	—	Migraine with or without aura	Unilateral‐nVNS	EG = 56; CG = 57	No	Migraine days and responder rates
Diener et al. (2019)	Germany	ICHD‐II	EG: 142 F/23 M; CG: 138 F/29 M	EG = 43.5 ± 11.1; CG = 41.4 ± 12.3	12	24	Migraine with or without aura	Bi‐nVNS	EG = 165; CG = 167	EG: 169 AEs; CG: 172 AEs	Migraine days
Zhang et al. (2021)	China	ICHD‐II	NA	NA	4	NA	Episodic migraine without aura	1 Hz taVNS	EG = 33; CG = 26	No	Migraine days
Silberstein et al. (2016)	USA	ICHD‐II	EG: 26 F/4 M; CG: 27 F/2 M	EG = 40.5 ± 14.2; CG = 38.8 ± 11.1	8	16	Chronic migraine	Rt‐nVNS	EG = 30; CG = 29	EG: 12 AEs; CG: 8 AEs	Migraine days and attack frequency
Straube et al. (2015)	Germany	ICHD‐II	1 Hz: 18 F/4 M; 25 Hz: 21 F/3 M	1 Hz = 43.8 ± 11.5; 25 Hz = 39.3 ± 12.4	12	0	Chronic migraine	1 Hz taVNS; 25 Hz taVNS	1 Hz = 22; 25 Hz = 24	1 Hz: 31 AEs; 25 Hz: 70 AEs	Headache days
Juan et al. (2017)	China	ICHD‐II	EG: 7 F/33 M; CG: 8 F/32 M	EG = 38.63 ± 11.46; CG = 36.50 ± 10.37	12	NA	Episodic migraine	PMES	EG = 40; CG = 40	No	Migraine days, severity of migraine days
Deng et al. (2020)	China	ICHD‐III	PMES: 39 F/6 M; STS‐Afz: 37 F/8 M	PMES = 33.73 ± 9.74; STS‐Afz = 31.33 ± 5.96	12	NA	Episodic migraine with or without aura	PMES; STS‐Afz	PMES = 45; STS‐Afz = 45	PMES: 0 AEs; STS: 6 AEs	Migraine days, responder rate
Schoenen et al. (2013)	Belgium	ICHD‐II	EG: 31 F/3 M; CG: 30 F/3 M	EG = 34.59 ± 11.01; CG = 39.06 ± 9.87	12	NA	Migraine with or without aura	STS‐Afz	EG = 34; CG = 33	No	Migraine days, responder rate
Liu et al. (2017)	China	ICHD‐III	EG: 52 F/14 M; CG: 18 F/4 M	EG = 37.6 ± 10.5; CG = 44.27 ± 8.32	4	12	Migraine without aura	tONS‐2 Hz; tONS‐100 Hz; tONS‐2/100 Hz	EG = 66; CG = 22	EG: 1 AEs; CG: 0 AEs	Responder rate

*Note*: Participants represent the number of individuals included in the final analysis of each study; the total number randomized across all 28 trials was 1920. Sex ratios reflect the as‐randomized sample; participant numbers reflect the as‐analyzed sample. Discrepancies arise from post‐randomization exclusions.

Abbreviations: AE = adverse event, CG = control group, DLPFC = dorsal lateral prefrontal cortex, EG = experimental group, ICHD‐II = International Classification of Headache Disorders second edition, ICHD‐III = International Classification of Headache Disorders third edition, LDLPFC = left dorsal lateral prefrontal cortex, M1 = primary motor cortex, NA = not available, rTMS = repetitive transcranial magnetic stimulation, sTMS = single‐pulse TMS, tDCS = transcranial direct current stimulation, TMS = transcranial magnetic stimulation, VAS = visual analog scale.

## 3 Results

### 3.1 Literature Search and Study Characteristics

**FIGURE 1:**
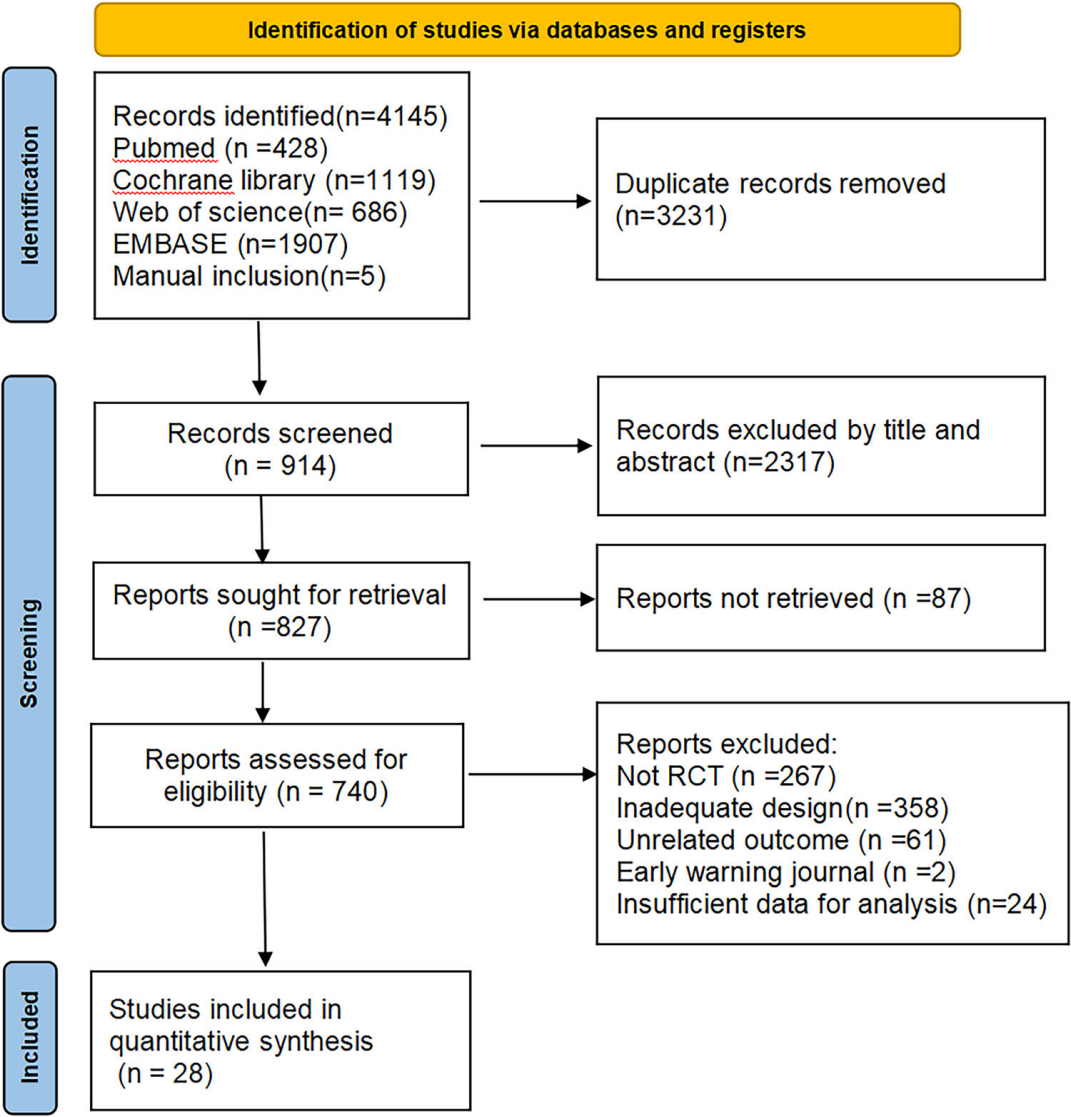
PRISMA flow diagram.

The literature selection process is detailed in the PRISMA flow diagram (Figure 1). The initial database search yielded 4145 records. After screening titles and abstracts, 914 articles were retrieved for full‐text eligibility assessment. Of these, 886 were excluded for specific reasons outlined in Figure 1, resulting in a final inclusion of 28 RCTs. A total of 1920 participants were randomized across the 28 studies. During the follow‐up period, 131 participants (6.8%) withdrew from the studies. The pooled sample consisted of 1381 female participants (70.1%); sex data were not reported in two studies. The reported mean age across studies ranged from 22 to 54.5 years. The included studies evaluated 24 distinct NIBS protocols. The most prevalent were tDCS (Pohl et al. 2021; Hodaj et al. 2022; Dalla Volta et al. 2020; DaSilva et al. 2023; Rahimi et al. 2020; Antal et al. 2011; Rocha et al. 2015; Andrade et al. 2017; Grazzi et al. 2020; Şirin et al. 2021; Auvichayapat et al. 2012; Aksu et al. 2023) (12 trials; *n* = 525), TMS (Teepker et al. 2010; Granato et al. 2019; Amin et al. 2020; Misra et al. 2013; Song et al. 2025; Conforto et al. 2014; Lipton et al. 2010) (7 trials; *n* = 401), and VNS (Najib et al. 2022; Diener et al. 2019; Zhang et al. 2021; Silberstein et al. 2016; Straube et al. 2015) (5 trials; *n* = 629). Additional protocols included transcutaneous mastoid electrical stimulation (PMES) (Juan et al. 2017; Deng et al. 2020) (*n* = 125), STS (Deng et al. 2020; Schoenen et al. 2013) (*n* = 112), and tONS (Liu et al. 2017) (*n* = 88). All trials adhered to the ICHD migraine diagnostic criteria. The patient populations primarily comprised CM (12 trials), EM (11 trials), and mixed or other populations (5 trials). Treatment cycles ranged from 3 days to 12 weeks. Follow‐up periods spanned from 4 to 48 weeks, with 4 and 12 weeks being the most common follow‐up durations. Characteristics of the included studies are summarized in Table 1. Of the 24 active treatment nodes, 18 were supported by only a single RCT (*k* = 1). Hf‐rTMS‐LDLPFC (*k* = 5), a‐tDCS‐M1‐C3 (*k* = 4), c‐tDCS‐Oz+a‐tDCS‐Cz (*k* = 2), 1Hz_taVNS (transcutaneous auricular vagus nerve stimulation) (*k* = 2), STS‐Afz (*k* = 2), and PMES (*k* = 2) were supported by more than one study. The number of studies (*k*) and total sample size (*N*) for each treatment node are reported in Table S4 (node‐level transitivity assessment).

### 3.2 Primary Outcome: The Changes in MMD

**FIGURE 2:**
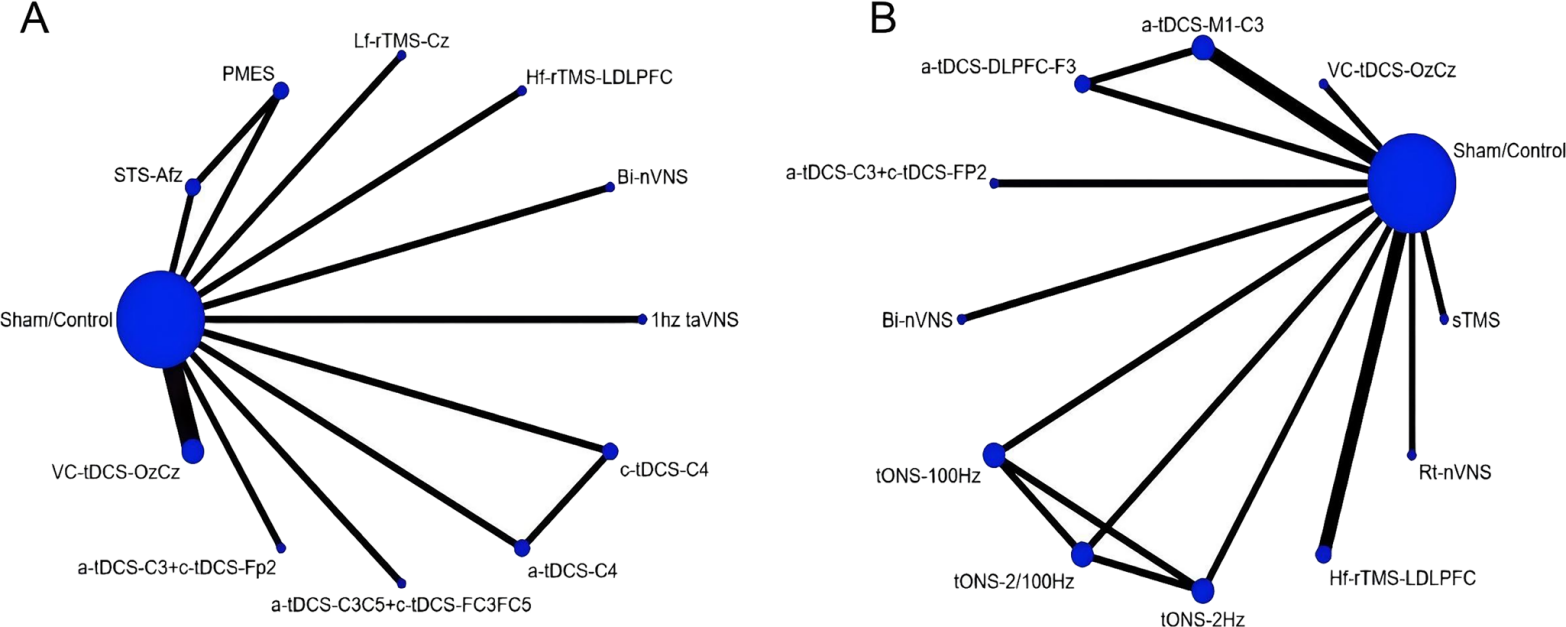
Network diagrams of direct evidence for (A) monthly migraine days and (B) dropout rate. The nodes represent the different interventions, with their size being proportional to the total number of participants (sample size) for each intervention. The lines connecting the nodes indicate direct comparisons from the included randomized controlled trials (RCTs), and the thickness of the lines is proportional to the number of trials for each comparison.

**FIGURE 3:**
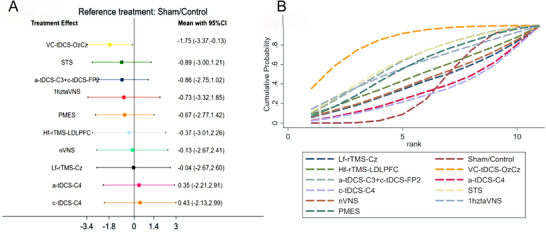
Network meta‐analysis of the change in monthly migraine days (MMDs). (A) Network forest plot comparing the effectiveness of interventions. The effect size is the mean difference (MD) with 95% confidence intervals (CIs). MD values less than 0 favor the intervention over the comparator. (B) SUCRA (surface under the cumulative ranking curve) plot ranking the interventions. The SUCRA value represents the probability of an intervention being the best treatment option, with higher values indicating a higher ranking.

Of the 11 interventions evaluated for MMD (Figure 2A), only VC‐tDCS‐OzCz showed a statistically significant reduction compared with the control group (MD = −1.75; 95% CI: −3.37 to −0.13) (Figure 3). SUCRA rankings identified VC‐tDCS‐OzCz as the most effective intervention (83.5%), followed by STS‐Afz (62.4%) and a‐tDCS‐C3+c‐tDCS‐FP2 (61.2%) (Table S5A), although the latter two did not reach statistical significance. Other protocols, including 1 Hz_taVNS (MD = −0.73; 95% CI: −3.32 to 1.85), also did not reach statistical significance. In the sensitivity analysis excluding studies with a high RoB, the effect estimate for VC‐tDCS‐OzCz remained similar in magnitude but was no longer statistically significant (MD = −1.78; 95% CI: −3.71 to 0.14) (Table S8A), with the wider CI reflecting the reduced precision of indirect evidence following the exclusion of studies from other nodes. These results highlight the influence of methodological quality on the precision of efficacy estimates for NIBS. The 95% prediction interval for VC‐tDCS‐OzCz versus sham/control was −5.00 to 1.50, crossing the null. Prediction intervals for additional key intervention‐versus‐sham/control comparisons also crossed the null and are summarized in Table S11.

### 3.3 Primary Outcome: The Reduction in the Dropout Rate

Among the 11 interventions evaluated for dropout rates (Figure 2B), only tONS (tONS‐2/100 Hz; RR = 0.36, 95% CI: 0.16–0.82) significantly reduced dropout rates compared to sham stimulation (Table 2). However, single‐pulse transcranial magnetic stimulation (sTMS; RR = 2.33), left M1 anodal tDCS (a‐tDCS‐M1‐C3; RR = 2.02), and high‐frequency rTMS stimulation of the left dorsolateral prefrontal cortex (Hf‐rTMS‐LDLPFC; RR = 1.22) showed higher dropout rates than the sham stimulation group (Figure S2G). In the SUCRA ranking for dropout rate, VC‐tDCS‐OzCz and tONS‐2/100 Hz ranked highest; however, only tONS‐2/100 Hz showed a statistically significant reduction in dropout rate versus sham/control (Table S5G).

**TABLE 2:** League table of the outcome of changes in dropout rate.

VC‐tDCS‐OzCz											
0.28 (0.02, 5.16)	tONS‐2/100Hz										
0.18 (0.01, 3.07)	0.62 (0.24, 1.61)	tONS‐100Hz									
0.18 (0.01, 3.07)	0.62 (0.24, 1.61)	1.00 (0.46, 2.19)	tONS‐2Hz								
0.13 (0.01, 2.26)	0.48 (0.18, 1.24)	0.76 (0.34, 1.68)	0.76 (0.34, 1.68)	Rt‐nVNS							
0.13 (0.00, 8.92)	0.47 (0.02, 12.33)	0.75 (0.03, 18.88)	0.75 (0.03, 18.88)	0.98 (0.04, 24.16)	a‐tDCS‐DLPFC‐F3						
0.12 (0.01, 2.01)	0.43 (0.18, 1.04)	0.69 (0.35, 1.38)	0.69 (0.35, 1.38)	0.91 (0.53, 1.56)	0.93 (0.04, 22.36)	Bi‐nVNS					
0.10 (0.00, 2.11)	0.36 (0.08, 1.57)	0.57 (0.14, 2.27)	0.57 (0.14, 2.27)	0.75 (0.20, 2.79)	0.77 (0.03, 22.86)	0.82 (0.24, 2.86)	a‐tDCS‐C3+c‐tDCS‐FP2				
0.10 (0.01, 1.63)	*0.36 (0.16, 0.82)	0.57 (0.30, 1.08)	0.57 (0.30, 1.08)	0.75 (0.47, 1.21)	0.77 (0.03, 18.21)	0.82 (0.64, 1.05)	1.00 (0.29, 3.39)	Sham/Control			
0.08 (0.00, 1.73)	0.29 (0.07, 1.29)	0.47 (0.12, 1.86)	0.47 (0.12, 1.86)	0.62 (0.16, 2.30)	0.63 (0.02, 18.75)	0.67 (0.19, 2.35)	0.82 (0.14, 4.63)	0.82 (0.24, 2.79)	HF‐rTMS‐LDLPFC		
0.05 (0.00, 1.34)	0.18 (0.03, 1.23)	0.28 (0.04, 1.83)	0.28 (0.04, 1.83)	0.37 (0.06, 2.30)	0.38 (0.02, 6.21)	0.41 (0.07, 2.40)	0.49 (0.06, 4.20)	0.49 (0.09, 2.87)	0.60 (0.07, 5.16)	a‐tDCS‐M1‐C3	
*0.04 (0.00, 0.94)	*0.15 (0.03, 0.73)	0.24 (0.06, 1.06)	0.24 (0.06, 1.06)	0.32 (0.08, 1.31)	0.33 (0.01, 10.15)	0.35 (0.09, 1.35)	0.43 (0.07, 2.59)	0.43 (0.11, 1.60)	0.52 (0.09, 3.17)	0.87 (0.10, 7.80)	sTMS

*Note*: Pairwise (upper‐right portion) and network (lower‐left portion) meta‐analysis results are presented as risk ratios (RRs) with 95% confidence intervals for all‐cause dropout rate. For dropout rate, RR < 1 indicates fewer dropouts and therefore better acceptability for the treatment specified by the relevant row/column orientation. Bold results marked with * indicate statistical significance.

### 3.4 Secondary Outcomes: Change in Pain Intensity, Attack Frequency, and Analgesic Use

Among the 15 interventions evaluating pain intensity (Figure S1F), none demonstrated statistical significance compared with the control group. Based on SUCRA ranking, low‐frequency repetitive transcranial magnetic stimulation (Lf‐rTMS‐Cz) had the highest probability of being optimal (SUCRA = 89.6%) (TableS 5F). Among the eight interventions evaluating attack frequency (Figure S1B), PMES (MD = −1.49, 95% CI: −3.32 to 0.33) and a‐tDCS‐M1‐C3 (MD = −1.38, 95% CI: −3.20 to 0.44) showed numerically greater reductions, but neither reached statistical significance (Figure S2B). PMES ranked highest by SUCRA (83.0%) and may represent a candidate intervention for future study (Table S5B). For analgesic use (12 interventions) (Figure S1H), only two interventions showed a statistically significant reduction compared with sham: anodal tDCS over the left M1 (C3) with the cathode over the right supraorbital region (Fp2) (MD = −0.93, 95% CI: −1.67 to −0.20) and bilateral nVNS (Bi‐nVNS) (MD = −0.66, 95% CI: −1.17 to −0.15) (Figure S2H). Consistent with these findings, these two interventions also achieved the highest SUCRA rankings, at 95.8% and 89.7%, respectively (Table S5H). Notably, the robustness of some results was compromised in sensitivity analyses; for instance, STS‐Afz and PMES, which were initially nonsignificant, became statistically significant in terms of pain intensity.

### 3.5 Secondary Outcomes: Duration, HIT‐6, Responder Rate, and Adverse Effects

Among the 10 interventions evaluating migraine duration (Figure S1D), only c‐M1‐tDCS‐C4 significantly shortened duration (MD = −1.34, 95% CI: −2.25 to −0.43) and achieved the highest SUCRA score (94.7%) (Figure S2D). Among the seven interventions evaluated for HIT‐6 scores (Figure S1E), cathodal tDCS over the right M1 cortex (c‐tDCS‐C4; MD = −0.86, 95% CI: −1.60 to −0.12) may be the most effective method for improving migraine's impact on daily life (Figure S2E). In terms of responder rate (Figure S1C), PMES and STS‐Afz significantly outperformed the control group. Regarding safety, only Hf‐rTMS‐LDLPFC demonstrated a higher risk of AEs (RR = 2.57, 95% CI: 1.05–6.29) (Figure S2I), while no statistically significant differences were observed for other interventions. Sensitivity analyses validated the robustness of our findings.

### 3.6 RoB, Publication Bias, Inconsistency, Quality of Evidence

**FIGURE 4:**
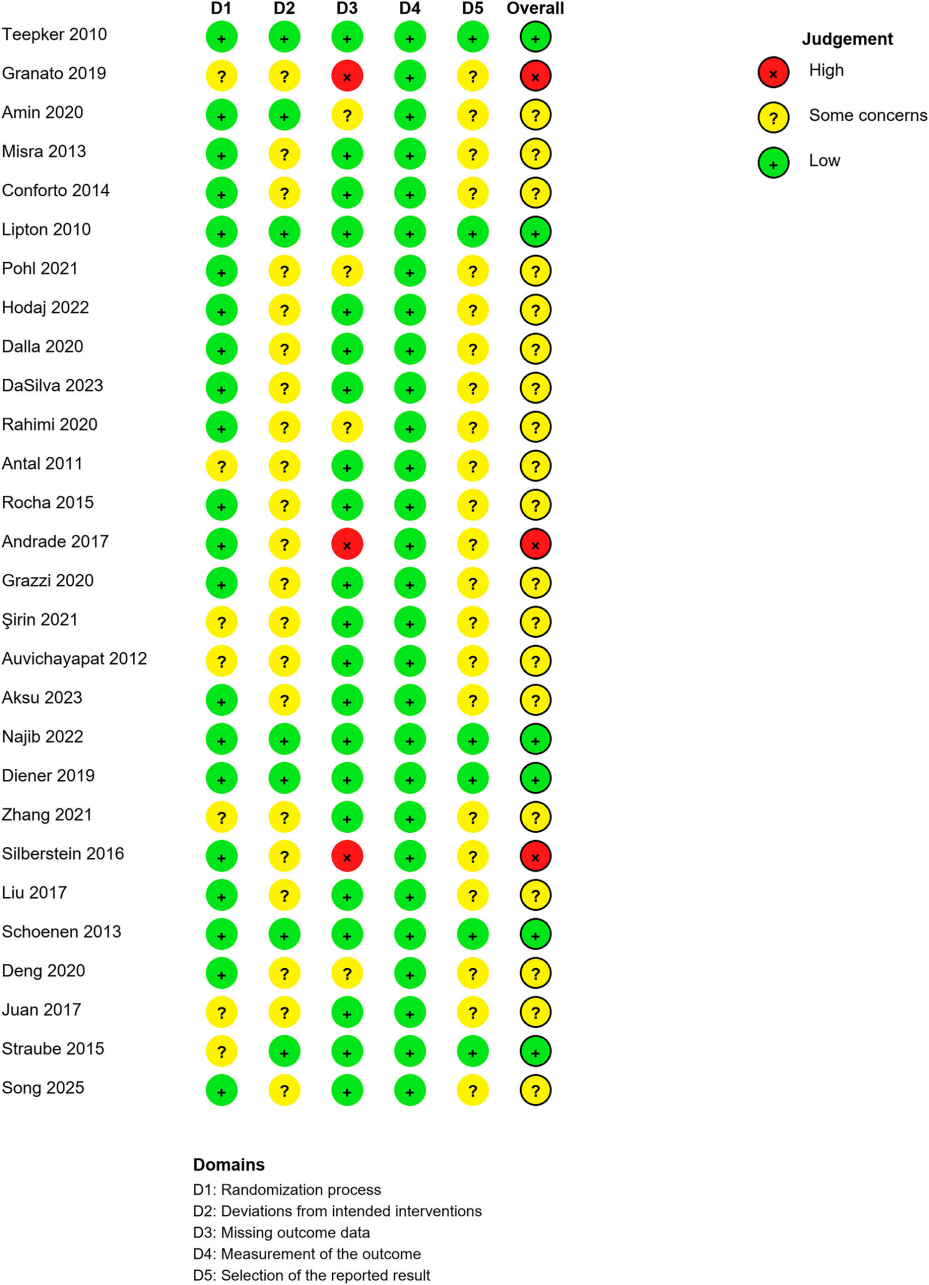
Risk of bias summary for each included study.

The overall RoB assessment for the 28 included studies is detailed in Figure 4. Six studies were at low RoB (21.4%). The majority of studies (67.9% [19/28]) were judged to have “some concerns,” while an additional three studies (10.7%) were at high RoB. The primary driver for these judgments was concerns related to incomplete outcome data (Domain 3 of the RoB 2.0 tool). Assessment of publication bias, using funnel plots and formal statistical tests (Egger's and Thompson–Sharp), did not reveal any significant evidence of small‐study effects (Figure S4). We assessed the network for both global and local inconsistency. The design‐by‐treatment interaction model revealed no significant global inconsistency across the network (*p* > 0.05). Furthermore, the node‐splitting analysis found no significant local inconsistency between direct and indirect evidence for any of the evaluated comparisons (*p* > 0.05). The certainty of the evidence for each outcome was evaluated using the GRADE framework. As detailed in Table S10A,B, the certainty of evidence for the primary outcomes was mostly rated as moderate or low. Substantial heterogeneity (*I* ^2^ ≥ 50%) was observed in two outcomes: MMD (*I* ^2^ = 71.3%) and pain intensity (*I* ^2^ = 94.7%). The remaining outcomes showed negligible heterogeneity: attack frequency (*I* ^2^ = 0%), responder rate (*I* ^2^ = 0%), migraine duration (*I* ^2^ = 0%), analgesic use (*I* ^2^ = 0%), AEs (*I* ^2^ = 0%), HIT‐6 (not estimable), and dropout rate (*I* ^2^ = 0%). Detailed heterogeneity statistics for all outcomes, including *Q* statistics and *Q*‐test *p* values, are provided in Table S7.

## 4 Discussion

This study presents a NMA of 28 RCTs, comparing the efficacy and acceptability of various NIBS techniques—including tDCS, rTMS, and multi‐target combined interventions—for the treatment of migraine. This analysis yields several key findings. Our primary finding is that VC‐tDCS‐OzCz was the most effective protocol for reducing MMD. This finding is protocol‐specific and should not be generalized to tDCS as a modality. However, as elaborated below, the evidence is of low certainty, derived from a single small trial, and its reproducibility remains uncertain. Prior work found that V1 inhibition reduces migraine days (Ahdab et al. 2019), consistent with our results. Although the optimal protocol here differs from Cheng et al. (2022), both suggest therapeutic potential at different cortical targets. Our estimate of −1.75 days is substantially more conservative than the −8.70 days reported by Cheng et al. (2022), primarily reflecting differences in included studies and analytical methods. Dawood Rahimi et al.’s (2023) emphasis on montage specificity over monopolar stimulation further supports this interpretation. The efficacy of tDCS in migraine is theoretically grounded: Anodal frontal stimulation may modulate thalamocortical circuits (Naegel et al. 2018), while cathodal occipital stimulation may interrupt cortical spreading depression (Ahdab et al. 2019). However, the traditional excitatory/inhibitory dichotomy oversimplifies tDCS effects, which depend on axonal orientation relative to the electric field (Naegel et al. 2018) and produce network‐level rather than focal modulation (Vaseghi et al. 2015). Future studies integrating tDCS with neuroimaging are needed to clarify mechanisms of action.

The −1.75 MMD reduction represents a 22%–44% decrease from the baseline 4–8 MMD typical of EM (Pohl et al. 2021), falling within the conventional MCID range of 1.0–1.5 MMD for EM. For CM (≥ 15 headache days/month), the same absolute reduction would represent a substantially smaller proportional benefit, and generalizability to this population remains unknown. The reduction is modest compared to CGRP monoclonal antibodies (−1.5 to −2.5 MMD vs. placebo), but VC‐tDCS‐OzCz offers a favorable safety profile. For patients who cannot tolerate or prefer to avoid pharmacotherapy, this safety advantage may partially offset the modest efficacy. Although VC‐tDCS‐OzCz achieved the highest SUCRA value (83.5%) for MMD reduction, several limitations warrant caution. First, the 95% CI (−3.37 to −0.13) approaches the null, indicating imprecision. Second, the 95% prediction interval crossed the null, and the benefit may not replicate in future studies. Third, the evidence is of low certainty (GRADE), based on a single trial of 11 participants (*k* = 1; RoB: some concerns), limiting the reliability of both the effect estimate and the ranking. The ranking primarily indicates that this intervention merits prioritization in future confirmatory trials, not that it should be adopted as a first‐line treatment. Other tDCS protocols—including a‐tDCS‐M1‐C3, c‐tDCS‐FC, and a‐tDCS‐C3+c‐tDCS‐FP2—did not significantly reduce MMD, reinforcing the protocol‐specific nature of tDCS effects. Only one of nine tDCS nodes reached statistical significance. Regarding safety and tolerability, analyses revealed no statistically significant difference in dropout rates between the vast majority of NIBS protocols and the control group, indicating overall good tolerability of NIBS. Except for Hf‐rTMS‐LDLPFC, other interventions showed no statistically significant difference in AE risk compared to the control group. Regarding responder rates, our study identified PMES as a highly effective intervention for improving patient response. Compared to patients receiving sham stimulation, those treated with PMES were approximately 4.5 times more likely to achieve a clinically effective response (RR = 4.46, 95% CI: 2.51–7.91). A key finding of this study is that the efficacy assessments of many NIBS techniques exhibit instability in sensitivity analyses. Specifically, after excluding studies with high RoB, certain interventions that did not show significant effects in the primary analysis—such as STS‐Afz and PMES for pain intensity—demonstrated statistical significance. This phenomenon was observed across multiple outcome measures, including pain intensity, attack frequency, analgesic dosage, and HIT‐6 scores, strongly indicating that the methodological quality of included studies is a decisive factor influencing the overall evidence synthesis in this field. A core challenge in current brain stimulation research lies in the heterogeneity of treatment protocols. Significant variations exist across studies in every aspect of design—from stimulation targets and electrode polarity to specific device parameters (such as intensity, frequency, and duration) and even the total number of treatment sessions. This lack of uniformity makes it difficult to establish a universally accepted optimal protocol and is a key source of discrepancies between studies and meta‐analyses. A promising direction is the combination of neuromodulation with pharmacotherapy, aligning with current personalized, multimodal pain management guidelines. Clinical trials (e.g., TACTIC, NCT05161871) (Ornello et al. 2025) have already begun exploring this approach. Several interventions with high SUCRA rankings are supported by limited evidence; VC‐tDCS‐OzCz and Lf‐rTMS‐Cz are supported by single small studies (*k* = 1, *n* ≤ 30), while PMES is supported by only two studies. In sparse networks, rankings are sensitive to individual trial estimates. A high SUCRA value indicates only the probability of ranking best within the analyzed set, not clinical superiority. Readers should evaluate each intervention holistically—considering effect size, uncertainty, evidence certainty, and evidential basis—rather than relying on rankings alone. The substantial heterogeneity for MMD (*I* ^2^ = 71.3%) and pain intensity (*I* ^2^ = 94.7%) likely reflects the diversity of populations, protocols, and measurement methods across the included studies. Patient samples ranged from EM to CM, treatment duration from 3 days to 12 weeks, and stimulation parameters varied even within nominally identical nodes (detailed in Tables S3 and S4). These sources of variability, elaborated in the limitations, may account for the observed *I* ^2^ values.

### 4.1 Limitations

This study also has several limitations. First, the analysis results revealed significant statistical heterogeneity. This may be attributed to differences among studies in baseline patient characteristics (such as migraine frequency and medication overuse), stimulation parameters, and statistical adjustment methods. Second, the included original studies themselves had limitations, including generally small sample sizes and short follow‐up periods. This constrained our statistical power for conducting in‐depth subgroup analyses and prevented assessment of the intervention's long‐term effects. Third, achieving effective blinding in neuromodulation trials is highly challenging. Active stimulation often induces skin sensations, potentially leading to unblinding by patients or investigators, which inevitably increases the RoB. Consequently, some observed efficacy may be attributable to placebo effects rather than the true effect of the intervention. Finally, this NMA relies heavily on indirect comparisons. Fourth, the included studies exhibited marked variability in treatment duration (ranging from 3 days to 12 weeks) and follow‐up length (4–48 weeks). Our prespecified time point selection rule enhances consistency but does not eliminate potential confounding from unequal treatment exposure. Fifth, owing to the sparse network structure (18 of 24 active nodes with *k* = 1), formal exploration of treatment duration, follow‐up length, or other potential effect modifiers via meta‐regression or subgroup analysis was not feasible; the specific sources of the observed heterogeneity therefore remain uncertain. Future RCTs should adopt standardized protocols with prespecified core outcome time points and extended follow‐up. This would both establish optimal treatment durations and help disentangle the clinical, methodological, and interventional sources of heterogeneity.

## 5 Conclusion

Among the interventions evaluated, VC‐tDCS‐OzCz showed the largest point estimate for reducing MMD (MD = −1.75; 95% CI: −3.37 to −0.13) and achieved the highest SUCRA ranking (83.5%). However, this finding must be interpreted with substantial caution given the low certainty of evidence (GRADE), the 95% prediction interval crossing the null, the sparse network structure, and unresolved transitivity concerns (detailed in the multidimensional assessment and limitations). Based on the available evidence, VC‐tDCS‐OzCz warrants further study in confirmatory trials but the current evidence is insufficient to support its recommendation as a first‐line preventive treatment. Adequately powered RCTs with standardized protocols, extended follow‐up, and direct head‐to‐head comparisons are required.

## Author Contributions

All authors made a significant contribution to the work reported, that is, in the conception, study design, execution, acquisition of data, analysis, and interpretation. All authors took part in drafting, revising, or critically reviewing the article; gave final approval of the version to be published; have agreed on the journal to which the article has been submitted; and agree to be accountable for all aspects of the work.

## Funding

This work was supported by the China‐Japan Friendship Hospital Elite Plan Talent Cultivation Project (ZRJY2024‐QMPY09).

## Ethics Statement

Ethics approval and informed consent were not required because this study was a systematic review and network meta‐analysis of previously published studies.

## Consent

The authors have nothing to report.

## Conflicts of Interest

The authors declare no conflicts of interest.

## Supporting information




**Supplementary Materials**: brb371566‐sup‐0001‐SuppMat.docx

## Data Availability

The original contributions presented in this study are documented in the article/Supporting Information. For further inquiries, please contact the corresponding author.

## References

[brb371566-bib-0001] Ahdab, R. , A. G. Mansour , G. Khazen , et al. 2019. “Cathodal Transcranial Direct Current Stimulation of the Occipital Cortex in Episodic Migraine: A Randomized Sham‐Controlled Crossover Study.” Journal of Clinical Medicine 9, no. 1: 60. 10.3390/jcm9010060. 31888011 PMC7019486

[brb371566-bib-0002] Aksu, S. , T. C. Şirin , B. R. Hasırcı Bayır , et al. 2023. “Long‐Term Prophylactic Transcranial Direct Current Stimulation Ameliorates Allodynia and Improves Clinical Outcomes in Individuals With Migraine.” Neuromodulation: Technology at the Neural Interface 26, no. 4: 778–787. 10.1016/j.neurom.2022.06.007. 35965182

[brb371566-bib-0003] Amin, R. , T. Emara , S. Ashour , et al. 2020. “The Role of Left Prefrontal Transcranial Magnetic Stimulation in Episodic Migraine Prophylaxis.” Egyptian Journal of Neurology, Psychiatry and Neurosurgery 56, no. 1: 19. 10.1186/s41983-019-0140-5.

[brb371566-bib-0004] Amiri, P. , S. Kazeminasab , S. A. Nejadghaderi , et al. 2022. “Migraine: A Review on Its History, Global Epidemiology, Risk Factors, and Comorbidities.” Frontiers in Neurology 12: 800605. 10.3389/fneur.2021.800605. 35281991 PMC8904749

[brb371566-bib-0005] Andrade, S. M. , R. E. L. de Brito Aranha , E. A. de Oliveira , et al. 2017. “Transcranial Direct Current Stimulation Over the Primary Motor vs Prefrontal Cortex in Refractory Chronic Migraine: A Pilot Randomized Controlled Trial.” Journal of the Neurological Sciences 378: 225–232. 10.1016/j.jns.2017.05.007. 28566169

[brb371566-bib-0006] Antal, A. , N. Kriener , N. Lang , K. Boros , and W. Paulus . 2011. “Cathodal Transcranial Direct Current Stimulation of the Visual Cortex in the Prophylactic Treatment of Migraine.” Cephalalgia 31, no. 7: 820–828. 10.1177/0333102411399349. 21398419

[brb371566-bib-0007] Auvichayapat, P. , T. Janyacharoen , A. Rotenberg , et al. 2012. “Migraine Prophylaxis by Anodal Transcranial Direct Current Stimulation, a Randomized, Placebo‐Controlled Trial.” Journal of the Medical Association of Thailand 95, no. 8: 1003–1012. 23061303

[brb371566-bib-0008] Cheng, Y.‐C. , B.‐Y. Zeng , C.‐M. Hung , et al. 2022. “Effectiveness and Acceptability of Noninvasive Brain and Nerve Stimulation Techniques for Migraine Prophylaxis: A Network Meta‐Analysis of Randomized Controlled Trials.” Journal of Headache and Pain 23, no. 1: 28. 10.1186/s10194-022-01401-3. 35184742 PMC8903676

[brb371566-bib-0009] Cohen, F. , H. Yuan , and S. D. Silberstein . 2022. “Calcitonin Gene‐Related Peptide (CGRP)‐Targeted Monoclonal Antibodies and Antagonists in Migraine: Current Evidence and Rationale.” Biodrugs 36, no. 3: 341–358. 10.1007/s40259-022-00530-0. 35476215 PMC9043885

[brb371566-bib-0010] Conforto, A. B. , E. Amaro , A. L. Gonçalves , et al. 2014. “Randomized, Proof‐Of‐Principle Clinical Trial of Active Transcranial Magnetic Stimulation in Chronic Migraine.” Cephalalgia 34, no. 6: 464–472. 10.1177/0333102413515340. 24326236

[brb371566-bib-0011] Dalla Volta, G. , S. Marceglia , P. Zavarise , and F. Antonaci . 2020. “Cathodal tDCS Guided by Thermography as Adjunctive Therapy in Chronic Migraine Patients: A Sham‐Controlled Pilot Study.” Frontiers in Neurology 11: 121. 10.3389/fneur.2020.00121. 32153497 PMC7047836

[brb371566-bib-0012] DaSilva, A. F. , D. J. Kim , M. Lim , et al. 2023. “Effect of High‐Definition Transcranial Direct Current Stimulation on Headache Severity and Central μ‐Opioid Receptor Availability in Episodic Migraine.” Journal of Pain Research 16: 2509–2523. 10.2147/JPR.S407738. 37497372 PMC10368121

[brb371566-bib-0013] Dawood Rahimi, M. , M. Taghi Kheirkhah , and J. Salehi Fadardi . 2023. “Efficacy of tDCS in Chronic Migraine: A Multiprotocol Randomized Controlled Trial.” Clinical Neurophysiology 150: 119–130. 10.1016/j.clinph.2023.03.013. 37060843

[brb371566-bib-0014] Deng, Y. , M. Zheng , L. He , J. Yang , G. Yu , and J. Wang . 2020. “A Head‐To‐Head Comparison of Percutaneous Mastoid Electrical Stimulator and Supraorbital Transcutaneous Stimulator in the Prevention of Migraine: A Prospective, Randomized Controlled Study.” Neuromodulation: Technology at the Neural Interface 23, no. 6: 770–777. 10.1111/ner.13127. 32096902

[brb371566-bib-0015] Diener, H.‐C. , P. J. Goadsby , M. Ashina , et al. 2019. “Non‐Invasive Vagus Nerve Stimulation (nVNS) for the Preventive Treatment of Episodic Migraine: The Multicentre, Double‐Blind, Randomised, Sham‐Controlled PREMIUM Trial.” Cephalalgia 39, no. 12: 1475–1487. 10.1177/0333102419876920. 31522546 PMC6791025

[brb371566-bib-0016] Edvinsson, L. , K. A. Haanes , K. Warfvinge , and D. N. Krause . 2018. “CGRP as the Target of New Migraine Therapies Successful Translation From Bench to Clinic.” Nature Reviews Neurology 14: 338–350. 10.1038/s41582-018-0003-1. 29691490

[brb371566-bib-0017] Goadsby, P. J. , P. R. Holland , M. Martins‐Oliveira , J. Hoffmann , C. Schankin , and S. Akerman . 2017. “Pathophysiology of Migraine: A Disorder of Sensory Processing.” Physiological Reviews 97, no. 2: 553–622. 10.1152/physrev.00034.2015. 28179394 PMC5539409

[brb371566-bib-0018] Granato, A. , J. Fantini , F. Monti , et al. 2019. “Dramatic Placebo Effect of High Frequency Repetitive TMS in Treatment of Chronic Migraine and Medication Overuse Headache.” Journal of Clinical Neuroscience 60: 96–100. 10.1016/j.jocn.2018.09.021. 30316627

[brb371566-bib-0019] Grazzi, L. , S. Usai , N. Bolognini , et al. 2020. “No Efficacy of Transcranial Direct Current Stimulation on Chronic Migraine With Medication Overuse: A Double Blind, Randomised Clinical Trial.” Cephalalgia 40, no. 11: 1202–1211. 10.1177/0333102420931050. 32536270

[brb371566-bib-0020] Headache Classification Committee of the International Headache Society (IHS) . 2018. “The International Classification of Headache Disorders, 3rd Edition.” Cephalalgia 38, no. 1: 1–211. 10.1177/0333102417738202. 29368949

[brb371566-bib-0021] Higgins, J. P. , C. Del Giovane , A. Chaimani , D. M. Caldwell , and G. Salanti . 2014. “Evaluating the Quality of Evidence From a Network Meta‐Analysis.” Value in Health 17, no. 7: A324. 10.1016/j.jval.2014.08.572. 27200533

[brb371566-bib-0022] Hodaj, H. , J.‐F. Payen , G. Mick , et al. 2022. “Long‐Term Prophylactic Efficacy of Transcranial Direct Current Stimulation in Chronic Migraine. A Randomised, Patient‐Assessor Blinded, Sham‐Controlled Trial.” Brain Stimulation 15, no. 2: 441–453. 10.1016/j.brs.2022.02.012. 35219923

[brb371566-bib-0023] Juan, Y. , O. Shu , L. Jinhe , et al. 2017. “Migraine Prevention With Percutaneous Mastoid Electrical Stimulator: A Randomized Double‐Blind Controlled Trial.” Cephalalgia 37, no. 13: 1248–1256. 10.1177/0333102416678623. 27821639

[brb371566-bib-0024] Lefaucheur, J.‐P. , A. Antal , S. S. Ayache , et al. 2017. “Evidence‐Based Guidelines on the Therapeutic Use of Transcranial Direct Current Stimulation (tDCS).” Clinical Neurophysiology 128: 56–92. 10.1016/j.clinph.2016.10.087. 27866120

[brb371566-bib-0025] Lipton, R. B. , D. W. Dodick , S. D. Silberstein , et al. 2010. “Single‐Pulse Transcranial Magnetic Stimulation for Acute Treatment of Migraine With Aura: A Randomised, Double‐Blind, Parallel‐Group, Sham‐Controlled Trial.” Lancet Neurology 9, no. 4: 373–380. 10.1016/S1474-4422(10)70054-5. 20206581

[brb371566-bib-0026] Liu, Y. , Z. Dong , R. Wang , et al. 2017. “Migraine Prevention Using Different Frequencies of Transcutaneous Occipital Nerve Stimulation: A Randomized Controlled Trial.” Journal of Pain 18, no. 8: 1006–1015. 10.1016/j.jpain.2017.03.012. 28428093

[brb371566-bib-0027] Misra, U. K. , J. Kalita , and S. K. Bhoi . 2013. “High‐Rate Repetitive Transcranial Magnetic Stimulation in Migraine Prophylaxis: A Randomized, Placebo‐Controlled Study.” Journal of Neurology 260, no. 11: 2793–2801. 10.1007/s00415-013-7072-2. 23963471

[brb371566-bib-0028] Naegel, S. , J. Biermann , N. Theysohn , et al. 2018. “Polarity‐Specific Modulation of Pain Processing by Transcranial Direct Current Stimulation—A Blinded Longitudinal fMRI Study.” Journal of Headache and Pain 19, no. 1: 99. 10.1186/s10194-018-0924-5. 30355321 PMC6755563

[brb371566-bib-0029] Najib, U. , T. Smith , N. Hindiyeh , et al. 2022. “Non‐Invasive Vagus Nerve Stimulation for Prevention of Migraine: The Multicenter, Randomized, Double‐Blind, Sham‐Controlled PREMIUM II Trial.” Cephalalgia 42, no. 7: 560–569. 10.1177/03331024211068813. 35001643

[brb371566-bib-0031] Ornello, R. , A. D'Atri , R. De Icco , et al. 2025. “Effectiveness of Transcranial Direct Current Stimulation and Monoclonal Antibodies Acting on the CGRP as a Combined Treatment for Migraine (TACTIC): Results of a Randomized Controlled Trial.” Cephalalgia 45, no. 5: 3331024251325567. 10.1177/03331024251325567. 40384614

[brb371566-bib-0032] Pohl, H. , M. Moisa , H.‐H. Jung , et al. 2021. “Long‐Term Effects of Self‐Administered Transcranial Direct Current Stimulation in Episodic Migraine Prevention: Results of a Randomized Controlled Trial.” Neuromodulation: Technology at the Neural Interface 24, no. 5: 890–898. 10.1111/ner.13292. 33078518

[brb371566-bib-0033] Puledda, F. , R. Messina , and P. J. Goadsby . 2017. “An Update on Migraine: Current Understanding and Future Directions.” Journal of Neurology 264, no. 9: 2031–2039. 10.1007/s00415-017-8434-y. 28321564 PMC5587613

[brb371566-bib-0034] Rahimi, M. D. , J. S. Fadardi , M. Saeidi , I. Bigdeli , and R. Kashiri . 2020. “Effectiveness of Cathodal tDCS of the Primary Motor or Sensory Cortex in Migraine: A Randomized Controlled Trial.” Brain Stimulation 13, no. 3: 675–682. 10.1016/j.brs.2020.02.012. 32289696

[brb371566-bib-0035] Rocha, S. , L. Melo , C. Boudoux , Á. Foerster , D. Araújo , and K. Monte‐Silva . 2015. “Transcranial Direct Current Stimulation in the Prophylactic Treatment of Migraine Based on Interictal Visual Cortex Excitability Abnormalities: A Pilot Randomized Controlled Trial.” Journal of the Neurological Sciences 349, no. 1–2: 33–39. 10.1016/j.jns.2014.12.018. 25579414

[brb371566-bib-0036] Salanti, G. , A. E. Ades , and J. P. A. Ioannidis . 2011. “Graphical Methods and Numerical Summaries for Presenting Results From Multiple‐Treatment Meta‐Analysis: An Overview and Tutorial.” Journal of Clinical Epidemiology 64, no. 2: 163–171. 10.1016/j.jclinepi.2010.03.016. 20688472

[brb371566-bib-0037] Schoenen, J. , B. Vandersmissen , S. Jeangette , et al. 2013. “Migraine Prevention With a Supraorbital Transcutaneous Stimulator: A Randomized Controlled Trial.” Neurology 80, no. 8: 697–704. 10.1212/WNL.0b013e3182825055. 23390177

[brb371566-bib-0038] Silberstein, S. D. , A. H. Calhoun , R. B. Lipton , et al. 2016. “Chronic Migraine Headache Prevention With Noninvasive Vagus Nerve Stimulation: The EVENT Study.” Neurology 87, no. 5: 529–538. 10.1212/WNL.0000000000002918. 27412146 PMC4970666

[brb371566-bib-0039] Şirin, T. C. , S. Aksu , B. R. H. Bayir , et al. 2021. “Is Allodynia a Determinant Factor in the Effectiveness of Transcranial Direct Current Stimulation in the Prophylaxis of Migraine?.” Neuromodulation 24, no. 5: 899–909. 34058041 10.1111/ner.13409

[brb371566-bib-0040] Song, P. , S. Li , Y. Shao , et al. 2025. “High Frequency‐rTMS of the Left DLPFC Relieve Headaches and Enhance Frontal‐Temporal Connectivity in Migraine.” Clinical Neurophysiology 173: 166–172. 10.1016/j.clinph.2025.03.019. 40147179

[brb371566-bib-0041] Straube, A. , J. Ellrich , O. Eren , B. Blum , and R. Ruscheweyh . 2015. “Treatment of Chronic Migraine With Transcutaneous Stimulation of the Auricular Branch of the Vagal Nerve (Auricular t‐VNS): A Randomized, Monocentric Clinical Trial.” Journal of Headache and Pain 16, no. 1: 63. 10.1186/s10194-015-0543-3. 26156114 PMC4496420

[brb371566-bib-0042] Teepker, M. , J. Hötzel , N. Timmesfeld , et al. 2010. “Low‐Frequency rTMS of the Vertex in the Prophylactic Treatment of Migraine.” Cephalalgia 30, no. 2: 137–144. 10.1111/j.1468-2982.2009.01911.x. 19515124

[brb371566-bib-0043] Vaseghi, B. , M. Zoghi , and S. Jaberzadeh . 2015. “How Does Anodal Transcranial Direct Current Stimulation of the Pain Neuromatrix Affect Brain Excitability and Pain Perception? A Randomised, Double‐Blind, Sham‐Control Study.” PLoS ONE 10, no. 3: e0118340. 10.1371/journal.pone.0118340. 25738603 PMC4349802

[brb371566-bib-0044] Whyte, C. A. , and S. J. Tepper . 2009. “Adverse Effects of Medications Commonly Used in the Treatment of Migraine.” Expert Review of Neurotherapeutics 9: 1379–1391. 10.1586/ern.09.47. 19769452

[brb371566-bib-0045] Zhang, Y. , Y. Huang , H. Li , et al. 2021. “Transcutaneous Auricular Vagus Nerve Stimulation (taVNS) for Migraine: An fMRI Study.” Regional Anesthesia & Pain Medicine 46, no. 2: 145–150. 10.1136/rapm-2020-102088. 33262253

