# Supplementary material for: Comparative Efficacy and Acceptability of Noninvasive Brain Stimulation for Migraine: A Systematic Review and Network Meta‐Analysis of Randomized Controlled Trials
Source: Brain Behav. 2026 Jul 19;16(7):e71566. doi: 10.1002/brb3.71566 (PMC13382076; doi:10.1002/brb3.71566)

| ****Supplementary materials - Supplementary tables and Supplementary figures**** | **Page** |
| --- | --- |
| eTable 1. PRISMA network checklist of systematic review | 2-5 |
| eTable 2. Detailed search strategy. | 6-8 |
| eTable 3. Characteristics of the included studies | 9-10 |
| eTable4：Transitivity Assessment | 10-11 |
| eTable 5. SUCRA | 11-17 |
| eTable 6. League table | 18-23 |
| eTable 7.Overall heterogeneity levels for each of the nine outcomes. | 24-27 |
| eTable 8. Sensitivity analysis | 28-37 |

| eTable 9. Risk of bias of each domain for each study | 38-39 |
| --- | --- |
| eTable 10.Quality of evidence for primary outcome | 40-44 |
| eTable 11. Prediction intervals for key intervention-versus-sham/control comparisons | 45 |
| eFigure 1. network structure | 44-47 |
| eFigure 2. forest map | 47-54 |
| eFigure 3. SUCRA optimization diagram | 51-56 |
| eFigure 4.Funnel plot and Egger/Thompson-Sharp test | 57-60 |

**eTable 1. PRISMA-NMA Checklist**

| **Section** | **Topic / Item** | **#** | **Checklist item** | **Reported on page / location** |
| --- | --- | --- | --- | --- |
| **TITLE** | **Title** | **1** | Identify the report as a systematic review incorporating a network meta-analysis. | Main manuscript p1 |
| **ABSTRACT** | **Structured summary** | **2** | Provide a structured summary including background, objectives, methods, eligibility criteria, synthesis methods, results, limitations, conclusions, funding, and registration where applicable. | Main manuscript p1–2 |
| **INTRODUCTION** | **Rationale** | **3** | Describe the rationale for the review in the context of what is already known, including why a network meta-analysis was conducted. | Main manuscript p2–4 |
|  | **Objectives** | **4** | Provide an explicit statement of the questions addressed with reference to participants, interventions, comparisons, outcomes, and study design (PICOS). | Main manuscript p. 1 and p 4–6 |
| **METHODS** | **Protocol and registration** | **5** | Indicate whether a review protocol exists and provide registration information where available. | Main manuscript p4 |
|  | **Eligibility criteria** | **6** | Specify study and report characteristics used as eligibility criteria, including PICOS, follow-up, years/language/publication status, eligible treatments, and node definitions. | Main manuscript p 4–7 |
|  | **Information sources** | **7** | Describe all information sources, including databases and date last searched. | Main manuscript p4; Supplementary eTable 2, p8 |
|  | **Search** | **8** | Present the full electronic search strategy for at least one database, including limits, so that it can be repeated. | Supplementary eTable 2, p8 |
|  | **Study selection** | **9** | State the process for selecting studies, including screening, eligibility assessment, and inclusion in the review/meta-analysis. | Main manuscript p4 and 10; Figure 1, p35 |
|  | **Data collection process** | **10** | Describe the method of data extraction and processes for obtaining or confirming data. | Main manuscript p5 |
|  | **Data items** | **11** | List and define variables for which data were sought and any assumptions or simplifications made. | Main manuscript p5–7; Table 1, p26; Supplementary eTables 3–4, p11 and 13 |
|  | **Geometry of the network** | **S1** | Describe methods used to explore network geometry and potential biases related to the evidence base. | Main manuscript p8–9; Figure 2, p37; Supplementary eFigure 1, p49 |
|  | **Risk of bias within individual studies** | **12** | Describe methods for assessing risk of bias within individual studies and how this was used in synthesis. | Main manuscript p6–7 |
|  | **Summary measures** | **13** | State the principal summary measures and additional summary measures such as SUCRA rankings. | Main manuscript p8–9 |
|  | **Planned methods of analysis** | **14** | Describe methods for handling data and combining study results for each network meta-analysis, including variance structure, multi-arm trials where applicable, and model assumptions. | Main manuscript p8–10 |
|  | **Assessment of inconsistency** | **S2** | Describe statistical methods used to evaluate agreement between direct and indirect evidence. | Main manuscript p9 |
|  | **Risk of bias across studies** | **15** | Specify assessments of bias that may affect the cumulative evidence, such as publication bias or selective reporting. | Main manuscript p9; Supplementary eFigure 5, p61 |
|  | **Additional analyses** | **16** | Describe methods for additional analyses, such as sensitivity analyses or subgroup/meta-regression if performed. | Main manuscript p10; Supplementary eTable 8, p31 |
| **RESULTS** | **Study selection** | **17** | Give numbers of studies screened, assessed for eligibility, and included, with reasons for exclusions at each stage, ideally with a flow diagram. | Main manuscript p10; Figure 1, p35 |
|  | **Presentation of network structure** | **S3** | Provide a network graph of the included studies to visualize treatment-network geometry. | Figure 2, p37; Supplementary eFigure 1, p49 |
|  | **Summary of network geometry** | **S4** | Provide an overview of network characteristics, including abundance of trials and participants across interventions and pairwise comparisons. | Main manuscript pp. 10–11; Supplementary eTable 4, p13 |
|  | **Study characteristics** | **18** | Present characteristics for each included study and provide citations. | Table 1, p26; Supplementary eTables 3–4, p11 and 13 |
|  | **Risk of bias within studies** | **19** | Present risk-of-bias data for each study and, if available, outcome-level assessment. | Figure 4, p39; Supplementary eTable 9, p40; Supplementary eFigure 4, p 60 |
|  | **Results of individual studies** | **20** | For all outcomes, present summary data by intervention group and effect estimates with confidence intervals; modified approaches may be used for large networks. | Figure 3, p38; Supplementary eFigures 2A–I, p 52; Supplementary eTables 6A–I, p 21–25 |
|  | **Synthesis of results** | **21** | Present results of each meta-analysis, including confidence intervals; league tables, forest plots, and treatment rankings may be used. | Main manuscript p10–13; Table 2, p33; Figure 3, p38; Supplementary eTables 5A–I, p14–19; eTables 6A–I, p21–25 |
|  | **Exploration for inconsistency** | **S5** | Describe results from investigations of inconsistency, including model-fit measures, statistical tests, or inconsistency estimates. | Main manuscript p13; Supplementary eTable 7, p 27 |
|  | **Risk of bias across studies** | **22** | Present results of assessments of bias across studies for the evidence base. | Main manuscript p13; Supplementary eFigure 4, p 61 |
|  | **Results of additional analyses** | **23** | Give results of additional analyses, if performed, such as sensitivity analyses or alternative model/network specifications. | Main manuscript p 11–13; Supplementary eTable 8, p 31 |
| **DISCUSSION** | **Summary of evidence** | **24** | Summarize main findings, including strength/certainty of evidence for each main outcome and clinical relevance. | Main manuscript p 14–16; Supplementary eTables 10A–B, p 42–43 |
|  | **Limitations** | **25** | Discuss limitations at study, outcome, and review levels, including assumptions of transitivity and consistency and concerns related to network geometry. | Main manuscript p16–17 |
|  | **Conclusions** | **26** | Provide a general interpretation of results in the context of other evidence and implications for future research. | Main manuscript p17 |
| **FUNDING** | **Funding** | **27** | Describe funding sources and other support for the systematic review and the role of funders. | Main manuscript p19 |

**eTable 2: Key word applied in each database and result**

| **Database** | **Search**  **number** | **keyword** | **R**  **es**  **ul**  **t** |
| --- | --- | --- | --- |
| PubMed | #1 | ("repetitive transcranial magnetic stimulation" OR rTMS[Title/Abstract] OR "Transcranial Magnetic  Stimulation" [Title/Abstract] OR TMS[Title/Abstract] OR "transcranial direct current stimulation" [Title/Abstract] OR  tDCS[Title/Abstract] OR "vagus nerve stimulation" [Title/Abstract] OR "vagal nerve stimulation" [Title/Abstract] OR  "transcutaneous vagus nerve stimulation" [Title/Abstract] OR tVNS[Title/Abstract] OR "Non-invasive vagus nerve  stimulation" [Title/Abstract] OR nVNS[Title/Abstract] OR "Vagus nerve stimulation" [Title/Abstract] OR VNS[Title/Abstract] OR "static magnetic field stimulation" [Title/Abstract] OR SMS[Title/Abstract] OR tSMS[Title/Abstract] OR "transcranial  alternating current stimulation" [Title/Abstract] OR tACS[Title/Abstract] OR "transcutaneous auricular vagus nerve  stimulation" [Title/Abstract] OR taVNS[Title/Abstract] OR "transcutaneous electrical nerve stimulation" [Title/Abstract] OR TENS[Title/Abstract] OR "Auricular Vagus Nerve Stimulation" [Title/Abstract] OR at-VNS[Title/Abstract] OR "transcranial occipital nerve stimulation" [Title/Abstract] OR "Percutaneous electrical nerve stimulation" [Title/Abstract] OR  PENS[Title/Abstract] OR "non-invasive brain stimulation" [Title/Abstract] OR "nerve stimulation" [Title/Abstract] OR  "electrical nerve stimulation" [Title/Abstract] OR "electrical stimulation" [Title/Abstract] OR "Non-invasive electrical nerve stimulation" [Title/Abstract] OR "non-invasive treatments" [Title/Abstract]) | 1438  58 |
|  | #2 | ("Migraine Disorders"[Mesh]) OR (headache*[Title/Abstract]) OR (migraine[Title/Abstract]) OR (migrain*[Title/Abstract]) | 1580  61 |
|  | #3 | (random[Title/Abstract] OR randomized[Title/Abstract] OR "randomized clinical trial" [Title/Abstract] OR RCT[Title/Abstract] OR placebo[Title/Abstract]) | 1317  885 |
|  | #4 | #1 AND #2 AND #3 | 428 |
| Cochrane library | #1 | MeSH descriptor: [Migraine Disorders] explode all trees | 3945 |
|  | #2 | ("Migraine Hemicrania":ti,ab,kw) OR ("Hemicrania Migraines":ti,ab,kw) OR ("Migraines Hemicrania":ti,ab,kw) OR ("Hemicrania Migraine":ti,ab,kw) OR ("Headaches Sick":ti,ab,kw) | 41 |
|  | #3 | ("Sick Headaches":ti,ab,kw) OR("Headache, Sick":ti,ab,kw) OR ("migraine headache":ti,ab,kw) OR ("Migraine Headaches":ti,ab,kw) OR  ("Migraine Disorder":ti,ab,kw) | 6679 |
|  | #4 | ("headache*":ti,ab,kw) OR ("migraine":ti,ab,kw) OR ("migraine*":ti,ab,kw) | 4388  0 |

| **Database** | **Search**  **number** | **keyword** | **R**  **es**  **ul**  **t** |
| --- | --- | --- | --- |
|  | #5 | #1 OR #2 OR #3 OR #4 | 4388  0 |
|  | #6 | MeSH descriptor: [Randomized Controlled Trial] explode all trees | 34 |
|  | #7 | ("Controlled Clinical Trials":ti,ab,kw) OR ("Randomized":ti,ab,kw) OR  ("Randomized Clinical":ti,ab,kw) OR ("Clinical Trials":ti,ab,kw) OR  ("random":ti,ab,kw) | 1333  160 |
|  | #8 | ("randomised":ti,ab,kw) OR ("randomized clinical trial":ti,ab,kw) OR  ("placebo control trial":ti,ab,kw) OR ("placebo controlled":ti,ab,kw) OR  ("RCT":ti,ab,kw) | 1298  873 |
|  | #9 | #6 OR #7 OR #8 | 1374  643 |
|  | #10 | ("repetitive transcranial magnetic stimulation":ti,ab,kw) OR  ("rTMS":ti,ab,kw) OR ("transcranial magnetic stimulation":ti,ab,kw) OR  ("TMS":ti,ab,kw) OR ("transcranial direct-current stimulation":ti,ab,kw) | 1716  4 |
|  | #11 | ("tDCS":ti,ab,kw) OR ("vagus-nerve stimulation":ti,ab,kw) OR  ("vagal-nerve stimulation":ti,ab,kw) OR ("transcutaneous vagus nerve stimulation":ti,ab,kw) OR ("tVNS":ti,ab,kw) | 8623 |
|  | #12 | ("Non-invasive vagus nerve stimulation":ti,ab,kw) OR ("nVNS":ti,ab,kw) OR ("vagus nerve stimulation":ti,ab,kw) OR ("VNS":ti,ab,kw) OR("static magnetic field stimulation":ti,ab,kw) | 2397 |
|  | #13 | ("SMS":ti,ab,kw) OR ("tSMS":ti,ab,kw) OR ("transcranial alternating current stimulation":ti,ab,kw) OR ("tACS":ti,ab,kw) OR ("transcutaneous auricular vagus nerve stimulation":ti,ab,kw) | 5027 |
|  | #14 | ("taVNS":ti,ab,kw) OR ("transcutaneous electrical nerve stimulation":ti,ab,kw) OR ("TENS":ti,ab,kw) OR ("Auricular Vagus Nerve Stimulation":ti,ab,kw) OR ("at-VNS":ti,ab,kw) | 6156 |
|  | #15 | ("transcranial occipital nerve stimulation":ti,ab,kw) OR ("Percutaneous electrical nerve stimulation":ti,ab,kw) OR ("PENS":ti,ab,kw) OR ("non-invasive brain stimulation":ti,ab,kw) OR ("nerve stimulation":ti,ab,kw) | 1549  9 |
|  | #16 | ("electrical nerve stimulation":ti,ab,kw) OR ("electrical stimulation":ti,ab,kw) OR ("Non-invasive electrical nerve stimulation":ti,ab,kw) OR ("non-invasive treatments":ti,ab,kw) | 1552  6 |
|  | #17 | #10 OR #11 OR #12 OR #13 OR #14 OR #15 OR #16 | 4348  7 |
|  | #18 | #5 AND #9 AND #17 | 1119 |
| Embase | #1 | ('non invasive brain stimulation'/exp OR 'non invasive brain stimulation':ab,ti OR 'electrical nerve stimulation'/exp OR  'electrical nerve stimulation':ab,ti OR 'electrostimulation'/exp OR 'electrostimulation':ab,ti OR 'nerve stimulation'/exp OR 'nerve | 3712  00 |

| **Database** | **Search**  **number** | **keyword** | **R**  **es**  **ul**  **t** |
| --- | --- | --- | --- |
|  |  | stimulation':ab,ti OR'non-invasive treatments':ab,ti OR 'non-invasive electrical stimulation':ab,ti OR 'non-invasive nerve stimulation':ab,ti OR 'repetitive transcranial magnetic stimulation'/exp OR 'rTMS':ab,ti OR 'transcranial magnetic  stimulation'/exp OR 'TMS':ab,ti OR 'transcranial direct current stimulation'/exp OR 'tDCS':ab,ti OR 'vagal nerve  stimulation'/exp OR 'vagus nerve stimulation':ab,ti OR 'transcutaneous vagus nerve stimulation'/exp OR 'transcutaneous vagus nerve stimulation':ab,ti OR 'tvns':ab,ti OR 'tVNS':ab,ti OR 'non invasive vagus nerve stimulation'/exp OR 'nvns':ab,ti OR 'static magnetic field stimulation'/exp OR 'sms':ab,ti OR 'transcranial alternating current stimulation'/exp OR 'tacs':ab,ti OR  'transcutaneous auricular vagus nerve stimulation'/exp OR 'tavns':ab,ti OR 'transcutaneous electrical nerve stimulation'/exp OR 'tens':ab,ti OR 'auricular vagus nerve stimulation'/exp OR 'at-vns':ab,ti OR 'transcranial occipital nerve stimulation'/exp OR  'percutaneous electrical nerve stimulation'/exp OR 'pens':ab,ti) |  |
|  | #2 | ('migraine'/exp OR 'migraine':ab,ti) OR ('headache'/exp OR 'headache':ab,ti) | 4218  27 |
|  | #3 | ('randomized controlled trial'/exp OR 'randomized controlled trial':ab,ti) OR ('placebo':ab) OR ('clinical trial':ab,ti) OR ('controlled clinical trial':ab,ti) OR ('clinical study':ti,ab) | 1697  061 |
|  | #4 | #1 AND #2 AND #3 | 1907 |
| Web of science | #1 | ( TS=("repetitive transcranial magnetic stimulation" OR "rTMS" OR "Transcranial Magnetic Stimulation" OR "TMS") OR  TS=("transcranial direct current stimulation" OR "tDCS") OR TS=("vagus nerve stimulation" OR "vagal nerve stimulation" OR "VNS" OR "transcutaneous vagus nerve stimulation" OR "tVNS" OR "Non-invasive vagus nerve stimulation" OR "nVNS" OR "transcutaneous auricular vagus nerve stimulation" OR "taVNS" OR "Auricular Vagus Nerve Stimulation" OR "at-VNS") OR  TS=("static magnetic field stimulation" OR "SMS" OR "tSMS") OR TS=("transcranial alternating current stimulation" OR  "tACS") OR TS=("transcutaneous electrical nerve stimulation" OR "TENS") OR TS=("transcranial occipital nerve stimulation") OR TS=("Percutaneous electrical nerve stimulation" OR "PENS") OR TS=("non-invasive brain stimulation" OR "non-invasive electrical nerve stimulation" OR "non-invasive treatments") OR TS=("nerve stimulation" OR "electrical nerve stimulation" OR "electrical stimulation")) | 1867  16 |
|  | #2 | TS=(MigraineDisorders (Topic) or headache* (Topic) or migraine (Topic) or migrain* (Topic)) | 1022  33 |
|  | #3 | TS=(random (Topic) or randomized (Topic) or randomised (Topic) or randomized clinical trial (Topic) or RCT (Topic) or placebo (Topic)) | 1712  284 |
|  | #4 | #1 AND #2 AND #3 | 686 |

**eTable 3. Characteristics of the included studies**

| **Study** | **publication** | **Feature of Stimulator Device** | **Stimulation Electrode** | **Electrode Size** | **Intensity** | **Frequency** |
| --- | --- | --- | --- | --- | --- | --- |
| Pohl 2021[12] | Neuromodulation | DC-STIMULATOR PLUS, NeuroConn, Ilmenau, Germany | Anodal VC (Oz-Cz) | 35 cm² electrodes | 1 mA | NA |
| Hodaj 2022[13] | Brain Stimulation | tarstim neurostimulator (Neuroelectrics, Barcelona, Spain) | Anodal C3 + cathodal Fp2 | 35 cm² electrodes | 2 mA; 1.5 mA; 2 mA | NA |
| Dalla 2020[14] | Front Neurol | CE marked constant current stimulator (HDCkit—Newronika srl) | Frontal cortex | Cathodal 15 cm²; Anodal 35 cm² | 1000 μA | NA |
| DaSilva 2023[15] | Journal of Pain Research | M1 HD-tDCS stimulator (Soterix Medical Inc., NY, USA) | M1 | 35 cm² electrode | 1 mA | NA |
| Rahimi 2020[16] | Brain Stimulation | battery-driven current stimulator (Neurostim2 Brain Stimulation Device™; MedinaTebCo.) | Cathodal M1 (C4) or S1 (CP4) | 35 cm² electrode | 1 mA | NA |
| Antal 2011[17] | Cephalalgia | battery-driven constant current stimulator (NeuroConn, Ilmenau, Germany) | Cathodal VC | 35 cm² electrode | 2 mA | NA |
| Rocha 2015[18] | J Neurol Sci | battery-driven constant current stimulator (NeuroConn, Germany) | V1 Cathodal VC | 35 cm² electrode | 2 mA | NA |
| Andrade 2017[19] | J Neurol Sci | neurostimulator developed by TransCranial Technologies (Hong Kong, China) | LM1; DLPFC | 35 cm² electrode | 2 mA | NA |
| Grazzi 2020[20] | Cephalalgia | battery-driven constant current stimulator (BrainStim, EMS srl, Bologna, Italy) | RM1 (C4) | 35 cm² electrode | 1 mA | NA |
| Şirin 2021[21] | Neuromodulation | NeuroConn DC-Stimulator Plus programmable direct current stimulator | LM1 (C3) | 35 cm² electrode | 2 mA | NA |
| Auvichayapat 2012[22] | J Med Assoc Thai | surface sponge electrodes | LM1 | / | 2 mA | NA |
| Aksu 2023[23] | Neuromodulation | NeuroConn DC-Stimulator Plus programmable tDCS device (NeuroConn GmbH, Ilmenau, Germany) | M1 | / | 2 mA | NA |
| Teepker 2010[24] | Cephalalgia | MagPro compact, Dantec, Denmark | Vertex | Figure-of-eight, diameter 11 cm | 100% RMT | 1 |
| Granato 2019[25] | J Clin Neurosci | A figure-of-eight coil | DLPFC | / | 100% RMT | 20 |
| Amin 2020[26] | Neurology, Psychiatry and Neurosurgery | The coil | LDLPFC | Figure-of-eight (MC-B70) coil | 100% RMT | 5 |
| Misra 2013[27] | Neurology | Magstim Rapid-2 (Whiteland, Walsh, UK) | LDLPFC | Air-cooled figure-eight coil of 7 cm diameter | 70% RMT | 10 |
| Song 2025[28] | Clin Neurophysiol | Magstim Rapid 2 stimulator (Magstim, Co. Ltd, UK) | LDLPFC | A figure-of-eight coil with a diameter of 70 mm | 90% RMT | 20 |
| Conforto 2014[29] | Cephalalgia | MagPro X100 (Alpine Biomed) | LDLPFC | Figure-of-eight (MC-B70) coil | 110% RMT | 10 |
| Lipton 2010[30] | The Lancet Neurology | Cerena Transcranial Magnetic Stimulator | Occipital bone | / | 0.9 T | Single pulse |
| Najib 2022[31] | Cephalalgia | / | Vagus nerve | / | 60 mA | 25 |
| Diener 2019[32] | Cephalalgia | / | Vagus nerve | / | 60 mA | 25 |
| Zhang 2021[33] | Regional Anesthesia and Pain Medicine | / | Auricular branch of vagus nerve (left ear) | 40 × 40 mm | 10 mA | 1 |
| Silberstein 2016[34] | Neurology | GammaCore®, electroCore, LLC, Basking Ridge, NJ | Neck in the vicinity of the vagus nerve | 30 mm × 94 mm | 60 mA | NA |
| Straube 2015[35] | Journal of Headache and Pain | The NEMOS t-VNS device | Left ear | / | NR | 1 Hz; 25 Hz |
| Juan 2017[36] | Cephalalgia | / | Bilateral ear mastoid | / | 1.0–1.2 V | 1.8 kHz |
| Deng 2020[37] | Cephalalgia | / | PMES: bilateral mastoid area behind the ear; STS-Afz: supraorbital area (Afz) | / | 10 mA | PMES: 1.8 kHz; STS-Afz: 60 Hz |
| Schoenen 2013[38] | Neurology | Cefaly, STX-Med., Herstal, Belgium | Bilateral supratrochlear and supraorbital nerves | Electrodes (42 × 24 mm) | 16 mA | 60 |
| Liu 2017[39] | Pain | HANS-200A machine, JiSheng Medical Technology Limited Company, China | Bilateral occipital nerves | / | 5–12 mA (active arms; average approximately 9–10 mA) | 2 Hz; 100 Hz; 2/100 Hz |

**Abbreviation:**DLPFC = dorsolateral prefrontal cortex;LDLPFC = left dorsal lateral prefrontal cortex; LM1 = left primary motor cortex; M1 = primary motor cortex; NA = not applicable; RMT = resting motor threshold; S1 = primary somatosensory cortex; V1 = visual cortex; VC = visual cortex; Cz = vertex; Oz = occipital (midline).

**eTable4：Transitivity Assessment**

| **Treatment_Node** | **k** | **Total_N** | **Migraine_Type** | **Treatment_Duration** | **Followup_weeks** | **RoB_Summary** | **Key_Stimulation_Parameters** | **Contributing_Studies** |
| --- | --- | --- | --- | --- | --- | --- | --- | --- |
| VC-tDCS-OzCz | 1 | 11 | EM | 4 weeks | 16 | Some concerns | 1 mA anodal VC over Oz-Cz | Pohl 2021 |
| a-tDCS-C3+c-tDCS-FP2 | 1 | 14 | CM | 8 weeks | 12 | Some concerns | 2 mA anodal C3 + cathodal Fp2 | Hodaj 2022 |
| c-tDCS-FC | 1 | 28 | CM | 5 days | 16 | Low | 1 mA cathodal frontal cortex | Dalla 2020 |
| c-tDCS-Oz+a-tDCS-Cz | 2 | 22 | EM | 4–6 weeks | 4–8 | Some concerns (both) | 2 mA cathodal Oz + anodal Cz | Antal 2011 (N=12); Rocha 2015 (N=10) |
| a-tDCS-M1-C3 | 4 | 72 | EM + CM (mixed) | 3 days – 4 weeks | 4–12 | Some concerns (all) | 1–2 mA anodal M1 (C3) | Şirin 2021 (N=36); Auvichayapat 2012 (N=20); Aksu 2023 (N=11); Andrade 2017-M1 arm (~5) |
| a-tDCS-DLPFC-F3 | 1 | 4 | Refractory CM | 4 weeks | NR | Some concerns | 2 mA anodal DLPFC (F3) | Andrade 2017-DLPFC arm |
| a-tDCS-C4 | 1 | 50 | CM | 5 weeks | 48 | Some concerns | 1 mA anodal C4 | Grazzi 2020-anodal arm |
| c-tDCS-C4 | 1 | 49 | CM | 5 weeks | 48 | Some concerns | 1 mA cathodal C4 | Grazzi 2020-cathodal arm |
| HD-tDCS-M1 | 1 | 13 | EM ± aura | 2 weeks | 4 | Some concerns | 1 mA HD-tDCS over M1 (C3-C5 / FC3-FC5) | DaSilva 2023 |
| c-M1-tDCS-C4 | 1 | 15 | EM ± aura | 10 weeks | 48 | Some concerns | 1 mA cathodal M1 (C4) | Rahimi 2020-M1 arm |
| c-S1-tDCS-CP4 | 1 | 15 | EM ± aura | 10 weeks | 48 | Some concerns | 1 mA cathodal S1 (CP4) | Rahimi 2020-S1 arm |
| Hf-rTMS-LDLPFC | 5 | 88 | EM + CM (mixed) | 2–12 weeks | 4–12; NR in Conforto 2014 | Some concerns (all) | 5–20 Hz 70–110% RMT LDLPFC | Granato 2019 (N=7); Amin 2020 (N=13); Misra 2013 (N=47); Song 2025 (N=14); Conforto 2014 (N=7) |
| Lf-rTMS-Cz | 1 | 14 | CM | 5 days | 8 | Some concerns | 1 Hz 100% RMT vertex (Cz) | Teepker 2010 |
| sTMS | 1 | 82 | Migraine with aura | Single acute treatment, up to 3 attacks | 2 h; sustained response at 24/48 h | Low | 0.9 T single-pulse occipital | Lipton 2010 |
| Bi-nVNS | 1 | 165 | Migraine ± aura | 12 weeks | 24 | Low | 25 Hz 60mA bilateral cervical VNS | Diener 2019 |
| Unilateral-nVNS | 1 | 56 | Migraine ± aura | 12 weeks | NR | Low | 25 Hz 60 mA unilateral cervical VNS | Najib 2022 |
| Rt-nVNS | 1 | 30 | CM | 8 weeks | 16 | Some concerns | 60 mA right cervical VNS | Silberstein 2016 |
| 1Hz_taVNS | 2 | 55 | EM without aura + CM | 4–12 weeks | 0/NR | Some concerns | 1 Hz auricular stimulation (10 mA in Zhang; intensity NR in Straube) | Zhang 2021 (N=33); Straube 2015-1Hz arm (N=22) |
| 25Hz_taVNS | 1 | 24 | CM | 12 weeks | 0 | Some concerns | 25 Hz auricular stimulation; intensity NR | Straube 2015-25Hz arm (N=24) |
| tONS-2Hz | 1 | 22 | Migraine without aura | 4 weeks | 12 | Some concerns | 2 Hz, 5–12 mA bilateral occipital | Liu 2017-2Hz arm |
| tONS-100Hz | 1 | 22 | Migraine without aura | 4 weeks | 12 | Some concerns | 100 Hz, 5–12 mA bilateral occipital | Liu 2017-100Hz arm |
| tONS-2_100Hz | 1 | 22 | Migraine without aura | 4 weeks | 12 | Some concerns | 2/100 Hz, 5–12 mA bilateral occipital | Liu 2017-2/100Hz arm |
| STS-Afz | 2 | 79 | EM + migraine ± aura | 12 weeks | NR | Some concerns | 60 Hz, 10–16 mA supraorbital | Schoenen 2013 (N=34); Deng 2020-STS arm (N=45) |
| PMES | 2 | 85 | EM | 12 weeks | NR | Some concerns | 1.8kHz mastoid area | Deng 2020-PMES arm (N=45); Juan 2017 (N=40) |
| Sham/Control | NA | 770 | NA | NA | NA | NA | Reference arm (all sham/control groups pooled) | Unique sham/control participants; active comparator arms in head-to-head trials (e.g., Deng 2020 and Straube 2015) are counted under their own treatment nodes. |

The sham/control total (N=770) reflects unique sham/control participants and excludes active comparator arms in head-to-head trials (e.g., Deng 2020 and Straube 2015), which are counted under their own treatment nodes.

**eTable 5A: SUCRA of the changes of migraine days**

**Abbreviation** SUCRA: surface under the cumulative ranking curve;The SUCRA value ranges from 0 to 100%, and the higher, the better. PrBest= The probability of becoming the optimal intervention (%); MeanRank= Average ranking (the smaller, the better)


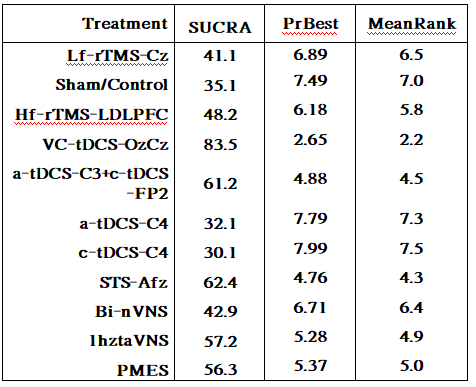


**eTable 5B: SUCRA of the attack frequency**


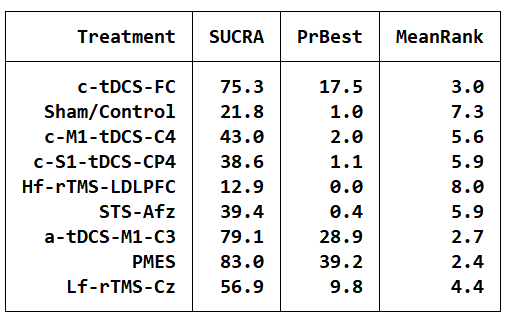


**eTable 5C: SUCRA of responder rate**


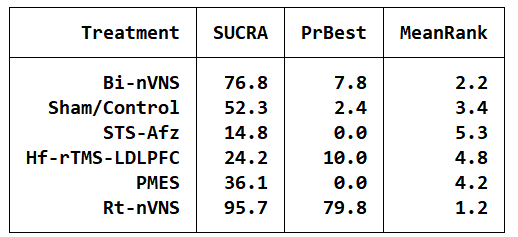


**eTable 5D: SUCRA of the duration**


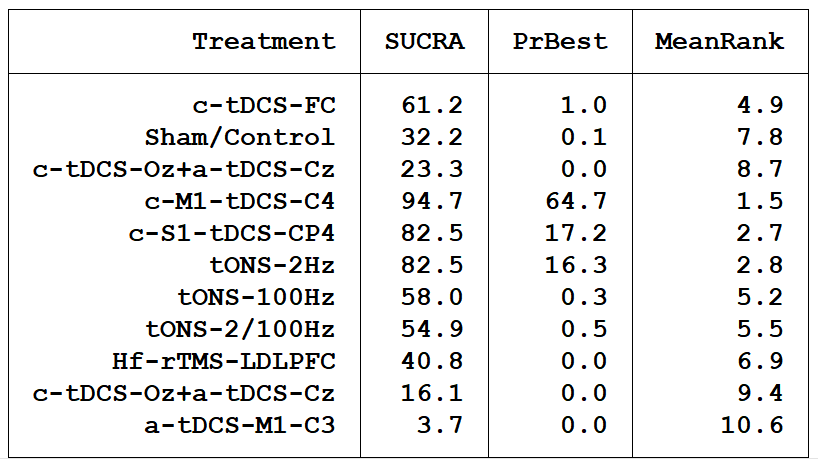


**eTable 5E: SUCRA of the HIT-6**


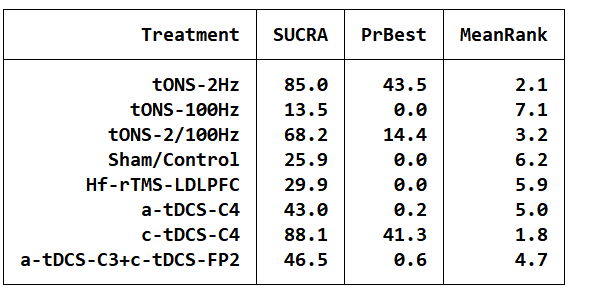


**eTable 5F: SUCRA of the pain intensity**


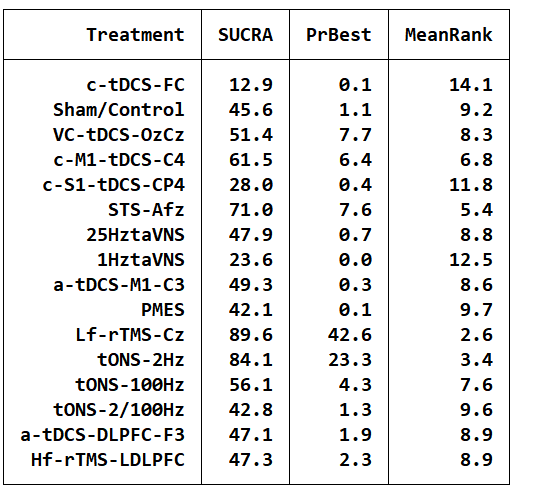


**eTable 5G: SUCRA of the drop-out rate**


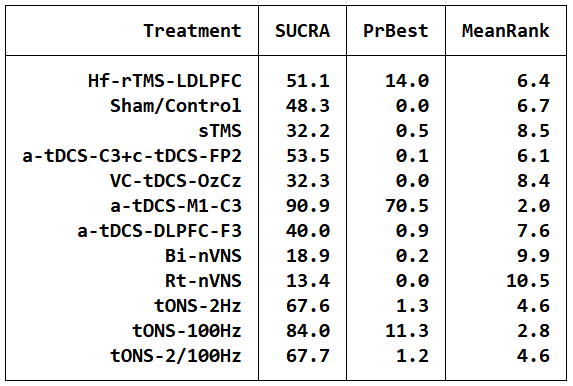


eTable 5H: SUCRA of analgesic use


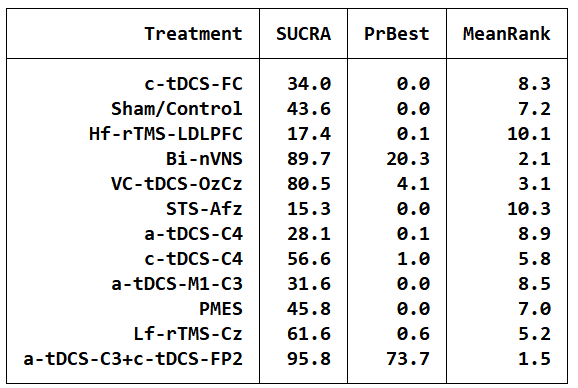


**eTable 5I: SUCRA of the adverse events**


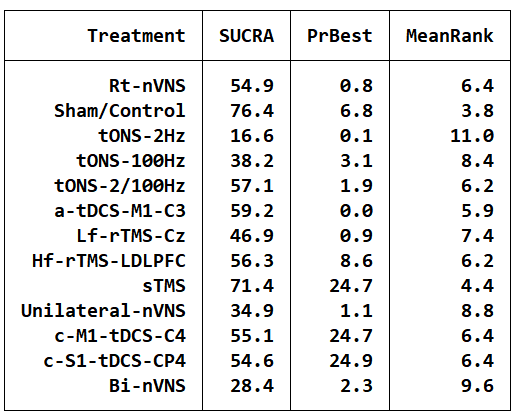


**eTable6A: League table of the outcome of changes in monthly migraine days**

| **VC-tDCS-OzCz** |  |  |  |  | |  | |  | |  |  | |  |  |  |
| --- | --- | --- | --- | --- | --- | --- | --- | --- | --- | --- | --- | --- | --- | --- | --- |
| -0.85 (-3.51,1.80) | **STS** |  |  |  |  |  |  |  |  |  |  |  |  |  |  |
| -0.88 (-3.37,1.61) | -0.03 (-2.86,2.80) | **a-tDCS-C3+c-tDCS-FP2** |  |  |  |  |  |  |  |  |  |  |  |  |  |
| -1.01 (-4.06,2.04) | -0.16 (-3.49,3.17) | -0.13 (-3.33,3.07) | **1hztaVNS** | |  | |  | |  |  |  |  | | | |
| -1.07 (-3.73,1.58) | -0.22 (-2.32,1.87) | -0.19 (-3.01,2.63) | -0.06 (-3.39,3.27) | | **PMES** | |  |  |  |  |  |  |  |  |  |
| -1.38 (-4.47,1.72) | -0.52 (-3.89,2.85) | -0.49 (-3.73,2.75) | -0.36 (-4.05,3.33) | | -0.30 (-3.67,3.07) | | **Hf-rTMS-LDLPFC** | |  |  |  |  |  |  |  |
| -1.62 (-4.63,1.39) | -0.76 (-4.06,2.53) | -0.73 (-3.90,2.43) | -0.60 (-4.22,3.02) | | -0.54 (-3.84,2.75) | | -0.24 (-3.90,3.42) | | **Bi-nVNS** |  |  |  |  |  |  |
| -1.71 (-4.81,1.38) | -0.86 (-4.23,2.52) | -0.83 (-4.07,2.42) | -0.70 (-4.39,3.00) | | -0.64 (-4.01,2.73) | | -0.34 (-4.06,3.39) | | -0.09 (-3.75,3.57) | **Lf-rTMS-Cz** |  |  |  |  |  |
| **-1.75 (-3.37,-0.13)** | -0.89 (-3.00,1.21) | -0.86 (-2.75,1.02) | -0.73 (-3.32,1.85) | | -0.67 (-2.77,1.42) | | -0.37 (-3.01,2.26) | | -0.13 (-2.67,2.41) | -0.04 (-2.67,2.60) | **Sham/Control** |  |  |  |  |
| -2.10 (-5.13,0.94) | -1.24 (-4.56,2.07) | -1.21 (-4.40,1.97) | -1.08 (-4.72,2.56) | | -1.02 (-4.33,2.29) | | -0.72 (-4.39,2.95) | | -0.48 (-4.08,3.13) | -0.38 (-4.06,3.29) | -0.35 (-2.91,2.21) | **a-tDCS-C4** | |  |  |

Pairwise (upper-right portion) and network (lower-left portion) meta-analysis results are presented as mean differences (MDs) with 95% confidence intervals for changes in monthly migraine days. Interventions are reported in order of mean ranking for monthly migraine days improvement. MD < 0 indicates a greater reduction in monthly migraine days for the treatment specified by the relevant row/column orientation. Bold results marked with * indicate statistical significance. Abbreviations: 95%CI: 95% confidence interval; a-tDCS-C3 + c-tDCS-FP2: anode tDCS over C3 and cathode tDCS over FP2; Hf-rTMS-DLPFC: high frequency rTMS over dorsolateral prefrontal cortex; Lf-rTMS-Cz: Low frequency rTMS over Cz; NIBS: noninvasive brain stimulation; NMA: network meta- analysis; RR: risk ratio; Sham: sham control; MD: mean difference; a-tDCS-C4: anode tDCS over C4; nVNS: non invasive vagal nerve stimulation;PMES:Transcutaneous Mastoid Electrical Stimulation;STS:Supraorbital transcutaneous stimulation;VC-tDCS- OzCz:Visual cortex- transcranial direct current stimulation over OzCz;taVNS:Transcutaneous auricular vagus nerve stimulation.

**eTable 6B: League table of the outcome of changes in attack frequency**

| PMES |  |  |  | c-M1-tDCS-C4 | |  |  |  |
| --- | --- | --- | --- | --- | --- | --- | --- | --- |
| -0. 11 (-1.37, 1. 15) | a-tDCS-M1-C3 |  |  |  |  |  |  |  |
| -0.30 (-1.81, 1.21) | -0. 19 (-1.70, 1.32) | c-tDCS-FC |  |  |  |  |  |  |
| -0.71 (-2.49, 1.07) | -0.60 (-2.37, 1. 18) | -0.41 (-1.84, 1.03) | Lf-rTMS-Cz |  |  |  |  |  |
| -0.99 (-2.59,0.60) | -0.88 (-2.47,0.71) | -0.69 (-1.90,0.51) | -0.28 (-1.81, 1.24) |  |  |  |  |  |
| -1.06 (-2.54,0.42) | -0.94 (-2.42,0.53) | -0.75 (-1.80,0.29) | -0.35 (-1.75, 1.05) | -0.06 (-1.22, 1. 10) | STS-Afz |  |  |  |
| -1.07 (-2.67,0.53) | -0.96 (-2.56,0.64) | -0.77 (-1.98,0.44) | -0.36 (-1.89, 1. 16) | -0.08 (-1.00,0.84) | -0.02 (-1.19, 1. 15) | c-S1-tDCS-CP4 |  |  |
| -1.49 (-3.32,0.33) | -1.38 (-3.20,0.44) | -1. 19 (-2.69,0.30) | -0.78 (-2.55,0.98) | -0.50 (-2.08, 1.08) | -0.44 (-1.90, 1.02) | -0.42 (-2.01, 1. 16) | Sham/Control |  |
| -1.51 (-2.81,-0.21) | -1.40 (-2.70,-0. 10) | -1.21 (-1.98,-0.44) | -0.80 (-2.01,0.41) | -0.52 (-1.44,0.40) | -0.46 (-1.16,0.25) | -0.44 (-1.37,0.49) | -0.02 (-1.30, 1.26) | Hf-rTMS-LDLPFC |

Pairwise (upper-right portion) and network (lower-left portion) meta-analysis results are presented as mean differences (MDs) with 95% confidence intervals for changes in attack frequency. Interventions are reported in order of mean ranking for attack-frequency reduction. MD < 0 indicates a greater reduction in attack frequency for the treatment specified by the relevant row/column orientation. Bold results marked with * indicate statistical significance. Abbreviations: 95%CI: 95% confidence interval; Hf-rTMS-DLPFC: high frequency rTMS over dorsolateral prefrontal cortex; Lf-rTMS-Cz: Low frequency rTMS over Cz; Sham: sham control; PMES:Transcutaneous Mastoid Electrical Stimulation;STS-Afz:Supraorbital transcutaneous stimulator over Afz;c-S1-tDCS-CP4:cathode S1-tDCS over CP4;c-M1-tDCS-C4:cathode M1-tDCS over C4

**eTable 6C: League table of the outcome of changes in responder rate**

| Sham/Control |  |  |  |  |  |
| --- | --- | --- | --- | --- | --- |
| 0.78 (0.56, 1. 10) | Bi-nVNS |  |  |  |  |
| 0.45 (0.20, 1.02) | 0.58 (0.24, 1.39) | Hf-rTMS-DLPFC |  |  |  |
| 0.28 (0.16, 0.51) | 0.36 (0. 18, 0.72) | 0.63 (0.23, 1.72) | STS-Afz |  |  |
| (0. 15 (0.01, 2.74) | 0. 19 (0.01, 3.57) | 0.33 (0.02, 6.78) | 0.52 (0.03, 10.27) | Rt-nVNS |  |
| 0.22 (0.13, 0.40) | 0.29 (0.15, 0.56) | 0.50 (0.18, 1.34) | 0.79 (0.61, 1.04) | 1.52 (0.08, 29.76) | PMES |

Pairwise (upper-right portion) and network (lower-left portion) meta-analysis results are presented as risk ratios (RRs) with 95% confidence intervals for responder rate. Interventions are reported in order of mean ranking of treatment response. For responder rate, RR > 1 indicates a higher response probability for the treatment specified by the relevant row/column orientation. Bold results marked with * indicate statistical significance. Abbreviations: 95%CI: 95% confidence interval; Hf-rTMS-DLPFC: high frequency rTMS over dorsolateral prefrontal cortex; PMES:Transcutaneous Mastoid Electrical Stimulation;STS-Afz:Supraorbital transcutaneous stimulator over Afz; Bi-nVNS: Bilateral vagus nerve stimulation; Rt-nVNS:Right vagus nerve stimulation;

**eTable 6D: League table of the outcome of changes in duration**

| c-M1-tDCS-C4 |  |  |  | tONS-100Hz | |  | Sham/Control | |  | |
| --- | --- | --- | --- | --- | --- | --- | --- | --- | --- | --- |
| -0.32 (-1.23,0.60) | c-S1-tDCS-CP4 |  |  |  |  |  |  |  |  |  |
| -0.32 (-1.23,0.60) | -0.00 (-0.72,0.72) | tONS-2Hz |  |  |  |  |  |  |  |  |
| -0.80 (-1.60,-0.00) | -0.49 (-1.46,0.49) | -0.49 (-1.46,0.49) | c-tDCS-FC |  |  |  |  |  |  |  |
| -0.87 (-1.66,-0.07) | -0.55 (-1.53,0.43) | -0.55 (-1.53,0.43) | -0.06 (-0.94,0.81) |  |  |  |  |  |  |  |
| -0.92 (-1.74,-0. 10) | -0.61 (-1.60,0.39) | -0.61 (-1.60,0.39) | -0. 12 (-1.01,0.77) | -0.06 (-0.95,0.83) | tONS-2/100Hz |  |  |  |  |  |
| -1. 19 (-1.97,-0.42) | -0.88 (-1.84,0.08) | -0.88 (-1.84,0.08) | -0.39 (-1.25,0.46) | -0.33 (-1.18,0.53) | -0.27 (-1.15,0.60) | Hf-rTMS-LDLPFC |  |  |  |  |
| -1.34 (-2.25,-0.43) | -1.03 (-2.10,0.04) | -1.03 (-2.10,0.04) | -0.54 (-1.52,0.44) | -0.48 (-1.45,0.50) | -0.42 (-1.41,0.57) | -0. 15 (-1. 11,0.81) |  |  |  |  |
| -1.45 (-1.96,-0.95) | -1. 14 (-1.90,-0.38) | -1. 14 (-1.90,-0.38) | -0.65 (-1.27,-0.03) | -0.59 (-1.20,0.03) | -0.53 (-1.17,0. 11) | -0.26 (-0.85,0.33) | -0. 11 (-0.87,0.65) | c-tDCS-Oz+a-tDCS-Cz |  |  |
| -1.63 (-2.41,-0.85) | -1.31 (-2.27,-0.35) | -1.31 (-2.27,-0.35) | -0.82 (-1.68,0.03) | -0.76 (-1.62,0.09) | -0.70 (-1.58,0. 17) | -0.43 (-1.03,0. 16) | -0.28 (-1.24,0.68) | -0. 17 (-0.76,0.42) | a-tDCS-M1-C3 |  |
| -1.93 (-2.71,-1. 15) | -1.62 (-2.58,-0.65) | -1.62 (-2.58,-0.65) | -1.13 (-1.99,-0.27) | -1.07 (-1.92,-0.21) | -1.01 (-1.89,-0. 13) | -0.74 (-1.34,-0. 14) | -0.59 (-1.55,0.37) | -0.48 (-1.07,0. 12) | -0.31 (-0.90,0.29) | Lf-rTMS-Cz |

Pairwise (upper-right portion) and network (lower-left portion) meta-analysis results are presented as mean differences (MDs) with 95% confidence intervals for changes in migraine duration. Interventions are reported in order of mean ranking for duration reduction. MD < 0 indicates a greater reduction in migraine duration for the treatment specified by the relevant row/column orientation. Bold results marked with * indicate statistical significance. Abbreviations: 95%CI: 95% confidence interval;c-tDCS-Oz + a-tDCS-Cz: cathode tDCS over Oz and anode tDCS over

Cz;Hf-rTMS-DLPFC: high frequency rTMS over dorsolateral prefrontal cortex; Lf-rTMS-Cz: Low frequency rTMS over Cz;Sham: sham control; c-S1-tDCS-CP4:cathode S1-tDCS over CP4;c-M1-tDCS-C4:cathode M1-tDCS over C4;c-tDCS-FC:cathode tDCS over FC; tONS: transcutaneous occipital nerve stimulation.

**eTable 6E: League table of the outcome of changes in HIT-6**

| tONS-2Hz |  |  |  |  |  |  |  |
| --- | --- | --- | --- | --- | --- | --- | --- |
| -0.01 (-1.00,0.98) | c-tDCS-C4 |  |  |  |  |  |  |
| -0.32 (-1.41,0.77) | -0.31 (-1.29,0.66) | tONS-2/100Hz |  |  |  |  |  |
| -0.64 (-1.61,0.34) | -0.63 (-1.23,-0.03) | -0.31 (-1.28,0.65) | a-tDCS-C3+c-tDCS-FP2 |  |  |  |  |
| -0.67 (-1.65,0.31) | -0.66 (-1.26,-0.06) | -0.35 (-1.32,0.62) | -0.03 (-0.63,0.56) | a-tDCS-C4 |  |  |  |
| -0.87 (-1.75,0.01) | -0.86 (-1.60,-0. 12) | -0.55 (-1.42,0.32) | -0.23 (-0.96,0.49) | -0.20 (-0.92,0.52) | Sham/Control |  |  |
| -0.83 (-1.71,0.05) | -0.83 (-1.56,-0.09) | -0.51 (-1.38,0.36) | -0.20 (-0.92,0.53) | -0. 16 (-0.89,0.56) | 0.04 (-0.38,0.45) | Hf-rTMS-LDLPFC |  |
| -0.96 (-1.74,-0. 19) | -0.96 (-1.57,-0.35) | -0.64 (-1.41,0. 12) | -0.33 (-0.92,0.26) | -0.29 (-0.89,0.30) | -0. 10 (-0.51,0.32) | -0.13 (-0.54,0.28) | tONS-100Hz |

Pairwise (upper-right portion) and network (lower-left portion) meta-analysis results are presented as mean differences (MDs) with 95% confidence intervals for changes in HIT-6 score. Interventions are reported in order of mean ranking for HIT-6 improvement. MD < 0 indicates a greater reduction in HIT-6 score for the treatment specified by the relevant row/column orientation. Bold results marked with * indicate statistical significance. Abbreviations: 95%CI: 95% confidence interval; a-tDCS-C3 + c-tDCS-FP2: anode tDCS over C3 and cathode tDCS over FP2;tONS: transcutaneous occipital nerve stimulation.Sham: sham control;Hf-rTMS-DLPFC: high frequency rTMS over dorsolateral prefrontal cortex; a-tDCS-C4:anode tDCS over C4;

**eTable 6F: League table of the outcome of changes in pain intensity**

| Lf-rTMS-Cz | tONS-2Hz | |  | tONS-100Hz | |  |  |  |  |  | |
| --- | --- | --- | --- | --- | --- | --- | --- | --- | --- | --- | --- |
| -0.43 (-3.00,2. 13) |  |  |  |  |  |  |  |  |  |  |  |
| -1.83 (-5.56, 1.90) | -1.40 (-5. 11,2.31) | c-M1-tDCS-C4 |  |  |  |  |  |  |  |  |  |
| -1.46 (-4.83, 1.91) | -1.03 (-4.38,2.32) | 0.38 (-2.95,3.70) | STS-Afz |  |  |  |  |  |  |  |  |
| -2.07 (-5.76, 1.62) | -1.64 (-5.31,2.03) | -0.24 (-3.88,3.41) | -0.61 (-3.89,2.66) |  |  |  |  |  |  |  |  |
| -2.32 (-6.78,2. 13) | -1.89 (-6.33,2.55) | -0.49 (-4.91,3.93) | -0.87 (-4.99,3.26) | -0.25 (-4.64,4. 13) | VC-tDCS-OzCz |  |  |  |  |  |  |
| -2.42 (-5.50,0.67) | -1.99 (-5.04, 1.07) | -0.58 (-3.61,2.45) | -0.96 (-3.53, 1.61) | -0.34 (-3.32,2.63) | -0.09 (-3.98,3.80) | a-tDCS-M1-C3 |  |  |  |  |  |
| -2.46 (-5.82,0.91) | -2.03 (-5.37, 1.32) | -0.62 (-3.94,2.69) | -1.00 (-3.05, 1.05) | -0.39 (-3.66,2.89) | -0.13 (-4.25,3.98) | -0.04 (-2.61,2.53) | 25HztaVNS |  |  |  |  |
| -2.52 (-6.20, 1. 16) | -2.09 (-5.75, 1.57) | -0.69 (-4.33,2.95) | -1.06 (-4.33,2.20) | -0.45 (-4.05,3. 14) | -0.20 (-4.58,4. 18) | -0. 11 (-3.07,2.86) | -0.07 (-3.33,3.20) | Hf-rTMS-LDLPFC |  |  |  |
| -2.69 (-6.37,0.99) | -2.26 (-5.92, 1.40) | -0.86 (-4.50,2.77) | -1.24 (-4.50,2.03) | -0.62 (-4.22,2.97) | -0.37 (-4.75,4.01) | -0.28 (-3.24,2.69) | -0.24 (-3.50,3.02) | -0. 17 (-2.70,2.36) | tONS-2/100Hz |  |  |
| -2.72 (-5.69,0.26) | -2.29 (-5.24,0.67) | -0.88 (-3.81,2.04) | -1.26 (-3.71, 1. 19) | -0.65 (-3.52,2.22) | -0.39 (-4.20,3.41) | -0.30 (-2.33, 1.73) | -0.26 (-2.70,2. 18) | -0.20 (-3.05,2.66) | -0.02 (-2.88,2.83) | PMES |  |
| -2.51 (-6.19, 1. 17) | -2.08 (-5.74, 1.58) | -0.68 (-4.31,2.96) | -1.05 (-4.32,2.22) | -0.44 (-4.03,3. 16) | -0. 19 (-4.56,4. 19) | -0.09 (-3.06,2.87) | -0.05 (-3.31,3.21) | 0.01 (-2.52,2.54) | 0. 18 (-2.35,2.72) | 0.21 (-2.65,3.07) | a-tDCS-DLPFC-F3 |
| -2.60 (-6.27, 1.07) | -2. 17 (-5.82, 1.48) | -0.77 (-4.39,2.85) | -1. 15 (-4.40,2. 11) | -0.53 (-4. 12,3.05) | -0.28 (-2.81,2.25) | -0. 19 (-3. 14,2.76) | -0. 15 (-3.40,3. 10) | -0.08 (-3.65,3.49) | 0.09 (-3.48,3.66) | 0. 11 (-2.73,2.96) | -0.09 (-3.67,3.48) |
| -3.45 (-7.16,0.26) | -3.02 (-6.71,0.67) | -1.62 (-5.28,2.05) | -1.99 (-5.29, 1.31) | -1.38 (-5.00,2.24) | -1.13 (-5.53,3.27) | -1.03 (-4.03, 1.97) | -0.99 (-4.29,2.30) | -0.93 (-4.54,2.69) | -0.76 (-4.37,2.86) | -0.73 (-3.62,2. 16) | -0.94 (-4.55,2.67) |
| -4.44 (-8.22,-0.67) | -4.01 (-7.77,-0.25) | -2.61 (-6.34, 1. 12) | -2.98 (-6.36,0.39) | -2.37 (-6.06, 1.32) | -2. 12 (-6.58,2.34) | -2.03 (-5. 11, 1.06) | -1.98 (-5.35, 1.39) | -1.92 (-5.60, 1.76) | -1.75 (-5.43, 1.93) | -1.72 (-4.33,0.88) | -1.93 (-5.62, 1.75) |
| -3.34 (-6.01,-0.67) | -2.91 (-5.55,-0.27) | -1.51 (-4. 12, 1. 10) | -1.88 (-3.95,0. 18) | -1.27 (-3.82, 1.28) | -1.02 (-4.59,2.55) | -0.93 (-2.47,0.61) | -0.89 (-2.94, 1. 17) | -0.82 (-3.35, 1.71) | -0.65 (-3.18, 1.88) | -0.62 (-1.94,0.69) | -0.83 (-3.37, 1.70) |

| Sham/Control |  |  |  |
| --- | --- | --- | --- |
| -0.85 (-4.45,2.76) | c-S1-tDCS-CP4 |  |  |
| -1.84 (-5.51, 1.83) | -0.99 (-4.70,2.72) | c-tDCS-FC |  |

| Sham/Control |  | | |
| --- | --- | --- | --- |
| -0.74 (-3.26, 1.78) | 0. 11 (-2.47,2.68) | 1. 10 (-1.57,3.77) | 1HztaVNS |

Pairwise (upper-right portion) and network (lower-left portion) meta-analysis results are presented as standardized mean differences (SMDs) with 95% confidence intervals for changes in pain intensity. Interventions are reported in order of mean ranking for pain-intensity reduction. SMD < 0 indicates a greater reduction in pain intensity for the treatment specified by the relevant row/column orientation. Bold results marked with * indicate statistical significance. Abbreviations: 95%CI: 95% confidence interval; Lf-rTMS-Cz: Low frequency rTMS over Cz; Sham: sham control; tONS: transcutaneous occipital nerve stimulation.VNS:vagus nerve stimulation.PMES:Transcutaneous Mastoid Electrical Stimulation; a-tDCS-DLPFC-F3: anode tDCS over DLPFC ;STS-Afz:Supraorbital transcutaneous stimulator over Afz;Hf-rTMS-DLPFC: high frequency rTMS over dorsolateral prefrontal cortex;VC-tDCS-OzCz:Visual cortex- transcranial direct current stimulation over OzCz; taVNS:Transcutaneous auricular vagus nerve stimulation;c-tDCS-FC:cathode tDCS over FC; c-S1-tDCS-CP4: PMES:Transcutaneous Mastoid Electrical Stimulation; Hf-rTMS-DLPFC: high frequency rTMS over dorsolateral prefrontal cortex;

**eTable 6G: League table of the outcome of changes in drop-out rate**

| VC-tDCS-OzCz |  |  |  |  |  |  |  | Sham/Control | |  | |
| --- | --- | --- | --- | --- | --- | --- | --- | --- | --- | --- | --- |
| 0.28 (0.02, 5. 16) | tONS-2/100Hz |  |  |  |  |  |  |  |  |  |  |
| 0. 18 (0.01, 3.07) | 0.62 (0.24, 1.61) | tONS-100Hz |  |  |  |  |  |  |  |  |  |
| 0. 18 (0.01, 3.07) | 0.62 (0.24, 1.61) | 1.00 (0.46, 2. 19) | tONS-2Hz |  |  |  |  |  |  |  |  |
| 0.13 (0.01, 2.26) | 0.48 (0.18, 1.24) | 0.76 (0.34, 1.68) | 0.76 (0.34, 1.68) | Rt-nVNS |  |  |  |  |  |  |  |
| 0.13 (0.00, 8.92) | 0.47 (0.02, 12.33) | 0.75 (0.03, 18.88) | 0.75 (0.03, 18.88) | 0.98 (0.04, 24. 16) | a-tDCS-DLPFC-F3 |  |  |  |  |  |  |
| 0. 12 (0.01, 2.01) | 0.43 (0.18, 1.04) | 0.69 (0.35, 1.38) | 0.69 (0.35, 1.38) | 0.91 (0.53, 1.56) | 0.93 (0.04, 22.36) | Bi-nVNS |  |  |  |  |  |
| 0. 10 (0.00, 2. 11) | 0.36 (0.08, 1.57) | 0.57 (0. 14, 2.27) | 0.57 (0. 14, 2.27) | 0.75 (0.20, 2.79) | 0.77 (0.03, 22.86) | 0.82 (0.24, 2.86) | a-tDCS-C3+c-tDCS-FP2 |  |  |  |  |
| 0. 10 (0.01, 1.63) | 0.36 (0.16, 0.82) | 0.57 (0.30, 1.08) | 0.57 (0.30, 1.08) | 0.75 (0.47, 1.21) | 0.77 (0.03, 18.21) | 0.82 (0.64, 1.05) | 1.00 (0.29, 3.39) |  |  |  |  |
| 0.08 (0.00, 1.73) | 0.29 (0.07, 1.29) | 0.47 (0. 12, 1.86) | 0.47 (0. 12, 1.86) | 0.62 (0.16, 2.30) | 0.63 (0.02, 18.75) | 0.67 (0.19, 2.35) | 0.82 (0. 14, 4.63) | 0.82 (0.24, 2.79) | HF-rTMS-LDLPFC |  |  |
| 0.05 (0.00, 1.34) | 0. 18 (0.03, 1.23) | 0.28 (0.04, 1.83) | 0.28 (0.04, 1.83) | 0.37 (0.06, 2.30) | 0.38 (0.02, 6.21) | 0.41 (0.07, 2.40) | 0.49 (0.06, 4.20) | 0.49 (0.09, 2.87) | 0.60 (0.07, 5. 16) | a-tDCS-M1-C3 |  |
| *0.04 (0.00, 0.94) | *0. 15 (0.03, 0.73) | 0.24 (0.06, 1.06) | 0.24 (0.06, 1.06) | 0.32 (0.08, 1.31) | 0.33 (0.01, 10. 15) | 0.35 (0.09, 1.35) | 0.43 (0.07, 2.59) | 0.43 (0. 11, 1.60) | 0.52 (0.09, 3. 17) | 0.87 (0.10, 7.80) | sTMS |

Pairwise (upper-right portion) and network (lower-left portion) meta-analysis results are presented as risk ratios (RRs) with 95% confidence intervals for all-cause dropout rate. Interventions are reported in order of mean ranking of acceptability. For dropout rate, RR < 1 indicates fewer dropouts and therefore better acceptability for the treatment specified by the relevant row/column orientation. Bold results marked with * indicate statistical significance. Abbreviations: 95%CI: 95% confidence interval; a-tDCS-C3 + c-tDCS-Fp2: anode tDCS overC3 and cathode tDCS over Fp2; Hf-rTMS-DLPFC: high frequency rTMS over dorsolateral prefrontal cortex;VC-tDCS-OzCz:Visual cortex- transcranial direct current stimulation over OzCz; Bi-nVNS: Bilateral vagus nerve stimulation; a-tDCS-M1-C3: tONS: transcutaneous occipital nerve stimulation; Sham/Control: sham control; a-tDCS-DLPFC-F3: sTMS: Rt-nVNS:Right vagus nerve stimulation;

**eTable 6H: League table of the outcome of changes in analgesic use**

| a-tDCS-C3+c-tDCS-FP2 | Bi-nVNS | |  |  |  |  |  |  | |
| --- | --- | --- | --- | --- | --- | --- | --- | --- | --- |
| -0.27 (-1.03,0.49) |  |  |  |  |  |  |  |  |  |
| -0.43 (-1.19,0.34) | -0. 16 (-0.51,0.20) | VC-tDCS-OzCz |  |  |  |  |  |  |  |
| -0.93 (-1.67,-0.20) | -0.66 (-1.17,-0. 15) | -0.51 (-1.03,0.01) | Sham/Control |  |  |  |  |  |  |
| -0.77 (-1.63,0.09) | -0.50 (-1.18,0. 17) | -0.35 (-1.03,0.34) | 0. 16 (-0.49,0.81) | c-tDCS-C4 |  |  |  |  |  |
| -0.74 (-1.51,0.04) | -0.47 (-1.03,0. 10) | -0.31 (-0.89,0.27) | 0.20 (-0.34,0.73) | 0.04 (-0.66,0.73) | Lf-rTMS-Cz |  |  |  |  |
| -0.91 (-1.66,-0. 16) | -0.64 (-1.17,-0. 11) | -0.48 (-1.03,0.06) | 0.02 (-0.48,0.52) | -0. 14 (-0.81,0.53) | -0. 17 (-0.73,0.38) | PMES |  |  |  |
| -1.05 (-1.82,-0.27) | -0.78 (-1.34,-0.21) | -0.62 (-1.20,-0.04) | -0. 11 (-0.65,0.42) | -0.27 (-0.97,0.42) | -0.31 (-0.72,0. 10) | -0. 14 (-0.69,0.42) | a-tDCS-M1-C3 |  |  |
| -1.03 (-1.72,-0.34) | -0.76 (-1.20,-0.32) | -0.60 (-1.06,-0. 15) | -0. 10 (-0.49,0.30) | -0.26 (-0.85,0.34) | -0.29 (-0.76,0. 18) | -0. 12 (-0.55,0.31) | 0.02 (-0.45,0.49) | c-tDCS-FC |  |
| -1. 18 (-1.83,-0.52) | -0.91 (-1.29,-0.52) | -0.75 (-1.15,-0.35) | -0.24 (-0.58,0.09) | -0.40 (-0.96,0. 15) | -0.44 (-0.86,-0.02) | -0.27 (-0.64,0. 10) | -0.13 (-0.55,0.29) | -0. 15 (-0.36,0.07) | a-tDCS-C4 |

Pairwise (upper-right portion) and network (lower-left portion) meta-analysis results are presented as mean differences (MDs) with 95% confidence intervals for analgesic use. Interventions are reported in order of mean ranking for analgesic-use reduction. MD < 0 indicates a greater reduction in analgesic use for the treatment specified by the relevant row/column orientation. Bold results marked with * indicate statistical significance. Abbreviations: 95%CI: 95% confidence interval; a-tDCS-C3 + c-tDCS-Fp2: anode tDCS overC3 and cathode tDCS over Fp2;VC-tDCS-OzCz:Visual cortex- transcranial direct current stimulation over OzCz; Bi-nVNS: Bilateral vagus nerve stimulation; PMES:Transcutaneous Mastoid Electrical Stimulation;c-tDCS-C4:cathode tDCS over C4; Sham/Control：sham control; Lf-rTMS-Cz: Low frequency rTMS over Cz; Bi-nVNS: Bilateral vagus nerve stimulation;

**eTable 6I: League table of the outcome of adverse events**

| Bi-nVNS |  |  |  |  |  |  |  |  |  |  |  |  |
| --- | --- | --- | --- | --- | --- | --- | --- | --- | --- | --- | --- | --- |
| 1.24 (0.24,  6.49) | C-S1-tDCS-CP  4 |  |  |  |  |  |  |  |  |  |  |  |
| 0.83 (0.66,  1.03) | 0.67 (0.13,  3.44) | Sham/Control |  |  |  |  |  |  |  |  |  |  |
| 0.81 (0.49,  1.33) | 0.65 (0.12,  3.56) | 0.97 (0.62,  1.53) | Rt-nVNS |  |  |  |  |  |  |  |  |  |
| 0.83 (0.19,  3.52) | 0.67 (0.13, | 1.00 (0.24, | 1.03 (0.23, | c-M1-tDCS-C |  |  |  |  |  |  |  |  |
|  | 3.44) | 4. 18) | 4.61) | 4 |  |  |  |  |  |  |  |  |
| 0.79 (0.53,  1. 15) | 0.63 (0.12, | 0.95 (0.69, | 0.97 (0.56, | 0.95 (0.22, | Unilateral-nVN |  |  |  |  |  |  |  |
|  | 3.36) | 1.30) | 1.69) | 4. 11) | S |  |  |  |  |  |  |  |
| 0.83 (0.02,  40. 18) | 0.67 (0.01,  44.85) | 1.00 (0.02,  48.24) | 1.03 (0.02,  50.87) | 1.00 (0.02,  62.30) | 1.05 (0.02,  51.50) | tONS-2/100Hz |  |  |  |  |  |  |
| 0.83 (0.02,  40. 18) | 0.67 (0.01,  44.85) | 1.00 (0.02,  48.24) | 1.03 (0.02,  50.87) | 1.00 (0.02,  62.30) | 1.05 (0.02,  51.50) | 1.00 (0.02,  48.24) | tONS-100Hz |  |  |  |  |  |
| 0.70 (0.39,  1.25) | 0.56 (0.10,  3. 16) | 0.84 (0.49,  1.44) | 0.87 (0.43,  1.75) | 0.84 (0.18,  3.89) | 0.89 (0.48, 1.66) | 0.84 (0.02,  42.22) | 0.84 (0.02,  42.22) | a-tDCS-M1-C  3 |  |  |  |  |
| 0.53 (0.16,  1.84) | 0.43 (0.06,  3.32) | 0.65 (0.19,  2. 18) | 0.66 (0.18,  2.43) | 0.65 (0.10,  4.23) | 0.68 (0.19, 2.39) | 0.65 (0.01,  37.55) | 0.65 (0.01,  37.55) | 0.77 (0.20,  2.89) | Lf-rTMS-Cz |  |  |  |
| 0.55 (0.24,  1.25) | 0.44 (0.07,  2.73) | 0.66 (0.30,  1.46) | 0.68 (0.27,  1.69) | 0.66 (0.13,  3.40) | 0.70 (0.30, 1.64) | 0.66 (0.01,  34.60) | 0.66 (0.01,  34.60) | 0.79 (0.30,  2.04) | 1.03 (0.24,  4.37) | sTMS |  |  |
| 0.28 (0.01,  6.47) | 0.22 (0.01,  7.72) | 0.33 (0.01,  7.75) | 0.34 (0.01,  8.23) | 0.33 (0.01,  10.57) | 0.35 (0.01, 8.30) | 0.33 (0.01,  7.75) | 0.33 (0.01,  7.75) | 0.40 (0.02,  9.62) | 0.52 (0.02,  15.06) | 0.50 (0.02,  12.91) | tONS-2Hz |  |
| 0.32 (0.13, | 0.26 (0.04, | 0.39 (0.16, | 0.40 (0.15, | 0.39 (0.07, | 0.41 (0.16, 1.06) | 0.39 (0.01, | 0.39 (0.01, | 0.46 (0.16, | 0.60 (0.13, | 0.59 (0.18, | 1. 17 (0.04, | Hf-rTMS-LDLPF |

| Bi-nVNS |  | | | | | | | | | | | |
| --- | --- | --- | --- | --- | --- | --- | --- | --- | --- | --- | --- | --- |
| 0.81) | 1.68) | 0.95) | 1.09) | 2. 11) |  | 20.79) | 20.79) | 1.31) | 2.73) | 1.94) | 30.78) | C |

Pairwise (upper-right portion) and network (lower-left portion) meta-analysis results are presented as risk ratios (RRs) with 95% confidence intervals for adverse events. Interventions are reported in order of mean ranking of safety. For adverse events, RR < 1 indicates fewer adverse events for the treatment specified by the relevant row/column orientation. Bold results marked with * indicate statistical significance. Abbreviations: 95%CI: 95% confidence interval; a-tDCS-C3 + c-tDCS-Fp2 anode tDCS overC3 and cathode tDCS over Fp2;Hf-rTMS-DLPFC: high frequency rTMS over dorsolateral prefrontal cortex;VC-tDCS-OzCz:Visual cortex- transcranial direct current stimulation over OzCz; Bi-nVNS: Bilateral vagus nerve stimulation; sTMS: single-pulse transcranial magnetic stimulation; a-tDCS-DLPFC-F3: tONS: transcutaneous occipital nerve stimulation. Rt-nVNS:Right vagus nerve stimulation;

**eTable 7. Overall heterogeneity levels for each of the nine outcomes and side-splitting inconsistency model.**

|  | **I^2^** | **Q** | ***P* value** |
| --- | --- | --- | --- |
| **Migraine days** | 71.3% | 10.46 | 0.015 |
| **Attack frequency** | 0% | 2.35 | 0.80 |
| **Response rate** | 0% | 1.32 | 0.72 |
| **Duration** | 0% | 0.73 | 1 |
| **HIT-6** | ----- | | |
| **Pain intensity** | 94.7% | 113.51 | < 0.0001 |
| **Drop-out rate** | 0% | 0.1 | 0.949 |
| **Analgesic** | 0% | 2.61 | 0.856 |
| **Adverse events** | 0% | 0.31 | 0.958 |

**Part 2: side-splitting inconsistency model (p > 0.05 indicates no statistical evidence of local inconsistency):**

1. **Migraine days**


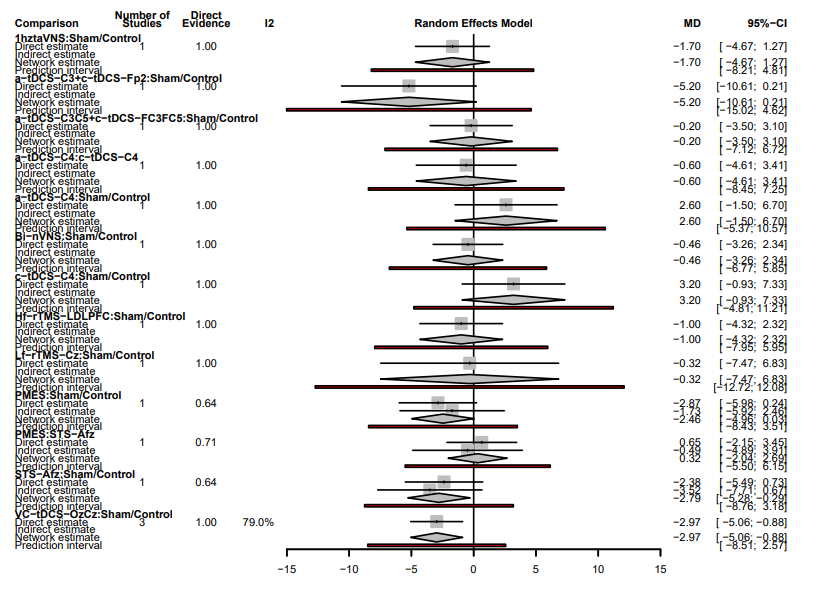


**B.Attack frequency**


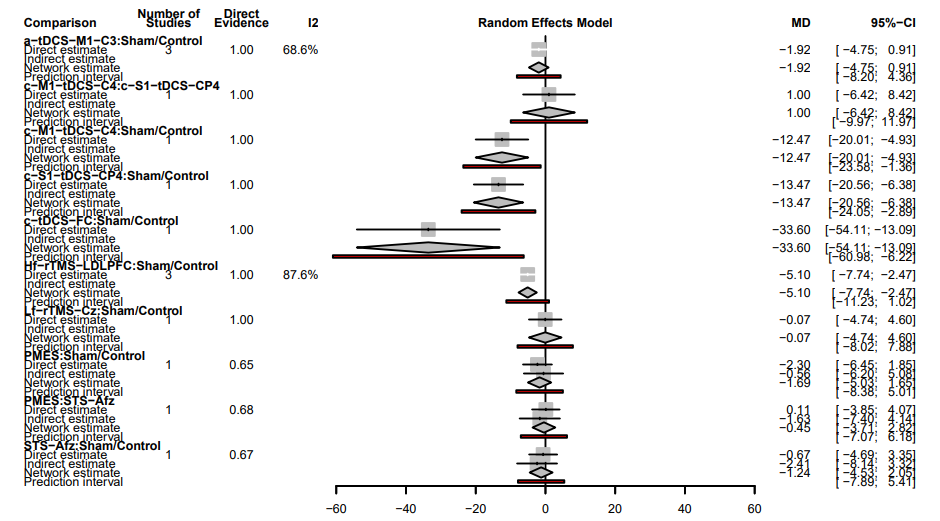


**C.Pain intensity**


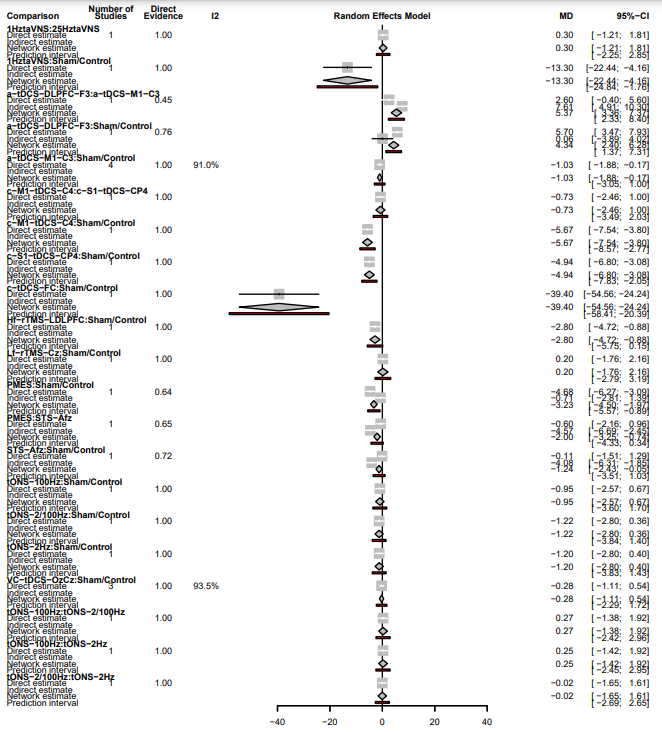


**D.Analgesic**


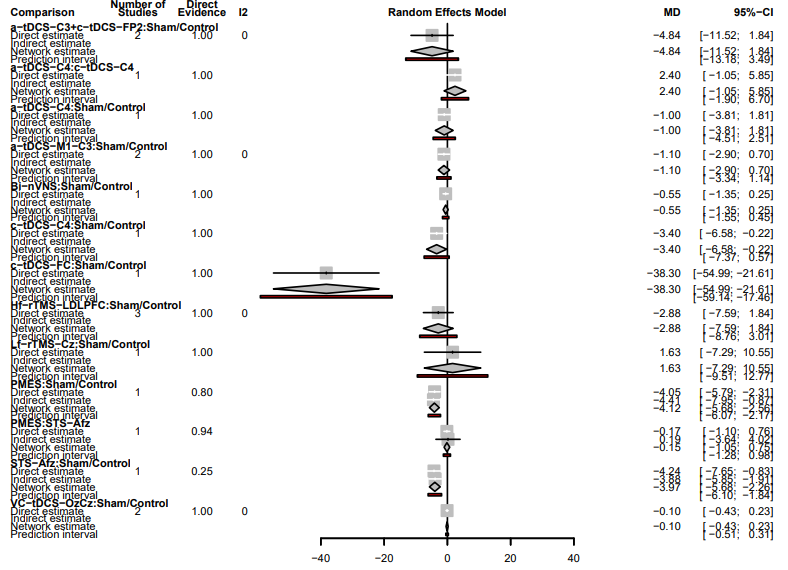


1. **Drop-out rate**


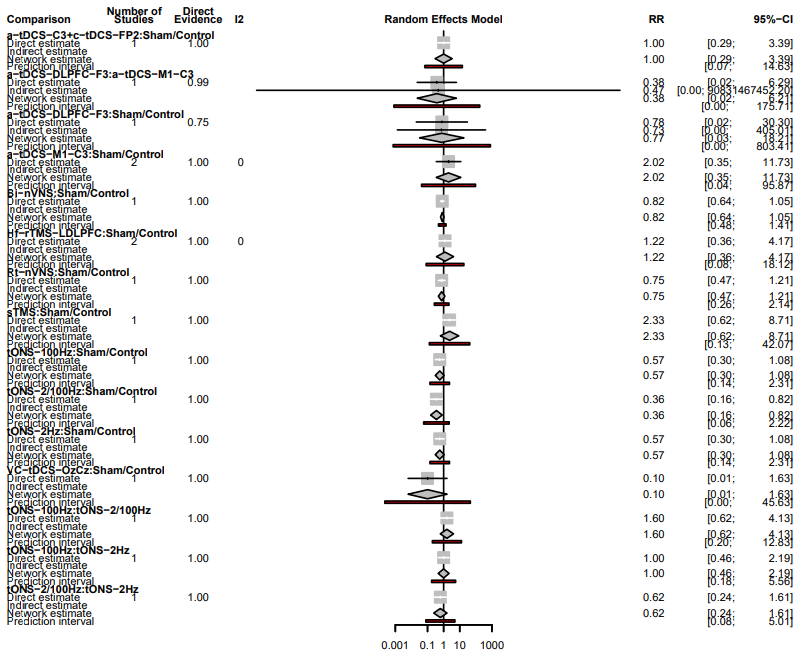


**F.Adverse events**


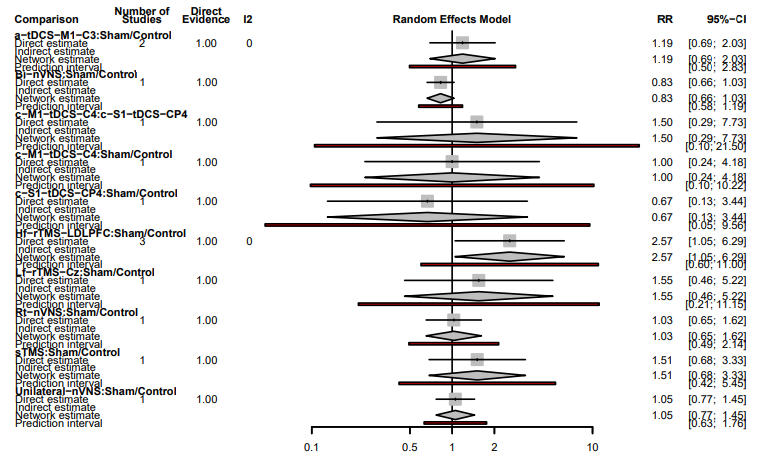


Duration、**Response Rate**、HIT-6 Only indirect comparison.

**eTable 8. Sensitivity analysis**

1. **Migraine days**

| **Treatment** | **Analysis** | **Result** |
| --- | --- | --- |
| Hf-rTMS-LDLPFC | Primary analysis | MD -0.37[95%-CI:-3.01,2.26] |
|  | Excluding studies with high overall risk of bias | MD -0.67 [95%CI: -2.94,2.94] |
|  | Using SMD as effect measure | SMD -0.38 [95%CI: -2.43,1.66] |
| VC-tDCS-OzCz | Primary analysis | MD -1.75[95%CI:-3.37,-0.13] |
|  | Excluding studies with high overall risk of bias | MD -1.78[95%CI:-3.71,0.14] |
|  | Using SMD as effect measure | SMD -1.79 [95%CI:-3.07,0.50] |
| a-tDCS-C3+c-tDCS-Fp2 | Primary analysis | MD -0.86[95%CI:-2.75,1.02] |
|  | Excluding studies with high overall risk of bias | MD -0.80[95%CI:-4.00,2.4] |
|  | Using SMD as effect measure | SMD -0.82[95%CI: -2.88,1.24] |
| 1hztaVNS | Primary analysis | MD -0.73[95%CI:-3.32,1.85] |
|  | Excluding studies with high overall risk of bias | MD -0.71[95%CI:-3.22,1.86] |
|  | Using SMD as effect measure | SMD -0.74[95% CI: -2.72, 1.24] |
| PMES | Primary analysis | MD -0.67[95%CI:-2.77,1.42] |
|  | Excluding studies with high overall risk of bias | MD -0.67[95%CI:-3.24,1.89] |
|  | Using SMD as effect measure | SMD -0.68 [95%CI: -2.29,0.92] |
| STS-Afz | Primary analysis | MD -0.89[95%CI:-3.00,1.21] |
|  | Excluding studies with high overall risk of bias | MD -0.89[95%CI:-3.00,1.21] |
|  | Using SMD as effect measure | SMD -0.89[95%CI:-3.00,1.21] |
| Bi-nVNS | Primary analysis | MD -0.13[95%CI:-2.67,2.41] |
|  | Excluding studies with high overall risk of bias | MD -0.13[95%CI:-2.67,2.41] |
|  | Using SMD as effect measure | SMD -0.13 [95%CI:-2.05,1.79] |
| Lf-rTMS-Cz | Primary analysis | MD -0.04[95%CI:-2.67,2.60] |
|  | Excluding studies with high overall risk of bias | MD -0.37[95%CI:-3.00,1.21] |
|  | Using SMD as effect measure | SMD -0.04 [95%CI:-2.09, 2.02] |
| a-tDCS-C4 | Primary analysis | MD 0.35[95%CI:-2.21,2.91] |
|  | Excluding studies with high overall risk of bias | MD-0.35[95%CI:-2.79,3.48] |
|  | Using SMD as effect measure | SMD 0.35 [95%CI: -1.60, 2.30] |
| c-tDCS-C4 | Primary analysis | MD 0.43[95%CI:-2.13,2.99] |
|  | Excluding studies with high overall risk of bias | MD -0.43[95%CI:-2.71,3.56] |
|  | Using SMD as effect measure | SMD 0.43 [95% CI -1.52,2.39] |

1. **Attack frequency**

| **Treatment** | **Analysis** | **Result** |
| --- | --- | --- |
| PMES | Primary analysis | MD -1.49 [95%-CI: -3.32, 0.33] |
|  | Excluding studies with high overall risk of bias | MD -2.30 [95%-CI: -8.37; 3.77] |
|  | Using SMD as effect measure | SMD -0.53 [95% CI -1.36,0.30] |
| c-tDCS-FC | Primary analysis | MD -1.19 [95%-CI: -2.69, 0.30] |
|  | Excluding studies with high overall risk of bias | MD -33.60 [95%-CI: -54.59, -12.61] |
|  | Using SMD as effect measure | SMD -0.82 [95% CI -1.92 , 0.28] |
| Hf-rTMS-LDLPFC | Primary analysis | MD 0.02 [95%CI: -1.26, 1.30] |
|  | Excluding studies with high overall risk of bias | MD -5.17 [95%CI: -8.86, -1.48] |
|  | Using SMD as effect measure | SMD -1.22 [95% CI -1.89, -0.55] |
| a-tDCS-M1-C3 | Primary analysis | MD -1.38 [95%-CI: -3.20, 0.44] |
|  | Excluding studies with high overall risk of bias | MD -4.10 [95%-CI: -9.46,1.27] |
|  | Using SMD as effect measure | SMD -0.47 [95% CI -1.11,0.18] |
| STS-Afz | Primary analysis | MD -0.44 [95%-CI: -1.90, 1.02] |
|  | Excluding studies with high overall risk of bias | MD -2.41 [95%-CI: -10.91,6.09] |
|  | Using SMD as effect measure | SMD -0.45 [95% CI -1.29 , 0.39] |
| Lf-rTMS-Cz | Primary analysis | MD -0.78 [95%-CI: -2.55, 0.98] |
|  | Excluding studies with high overall risk of bias | NA |
|  | Using SMD as effect measure | SMD -0.02 [95% CI -1.20 ,1.16] |
| c-M1-tDCS-C4 | Primary analysis | MD -0.50 [95%-CI: -2.08, 1.08] |
|  | Excluding studies with high overall risk of bias | NA |
|  | Using SMD as effect measure | SMD -1.43 [95% CI -2.62, -0.23] |
| c-S1-tDCS-CP4 | Primary analysis | MD -0.42 [95%-CI: -2.01, 1.16] |
|  | Excluding studies with high overall risk of bias | NA |
|  | Using SMD as effect measure | SMD -1.54 [95% CI -2.74, -0.34] |

**C.responder rate**

| **Treatment** | **Analysis** | **Result** |
| --- | --- | --- |
| Rt-nVNS | Primary analysis | RR 6.77 [95% CI 0.37 ,125.49] |
|  | Excluding studies with high overall risk of bias | RR 6.77 [95% CI 0.37,125.49] |
|  | Using OR as effect measure | OR 7.51 [95% CI 0.37 , 152.09] |
| Bi-nVNS | Primary analysis | RR 1.28 [95% CI 0.91 ,1.80] |
|  | Excluding studies with high overall risk of bias | NA |
|  | Using OR as effect measure | OR 1.41 [95% CI 0.87,2.27] |
| PMES | Primary analysis | RR 4.46 [95% CI 2.51,7.91] |
|  | Excluding studies with high overall risk of bias | RR 4.71 [95% CI 2.37, 9.38] |
|  | Using OR as effect measure | OR 16.92 [95% CI 6.67 ,42.95] |
| STS-Afz | Primary analysis | RR 3.53 [95% CI 1.95 ,6.41] |
|  | Excluding studies with high overall risk of bias | RR 3.77 [95% CI 1.80, 7.91] |
|  | Using OR as effect measure | OR 6.68 [95% CI 2.54 , 17.58] |
| Hf-rTMS-LDLPFC | Primary analysis | RR 2.22 [95% CI 0.98, 4.99] |
|  | Excluding studies with high overall risk of bias | RR 2.22 [95% CI 0.98 , 4.99] |
|  | Using OR as effect measure | OR 4.95 [95% CI 1.02 , 24.10] |

**D.Duration**

| **Treatment** | **Analysis** | **Result** |
| --- | --- | --- |
| c-tDCS-FC | Primary analysis | MD -0.54 [95% CI -1.52, 0.44] |
|  | Excluding studies with high overall risk of bias | MD -21.60 [95% CI -39.72, -3.48] |
|  | Using SMD as effect measure | SMD -0.60 [95% CI-1.21 – 0.02] |
| a-tDCS-M1-C3 | Primary analysis | MD 0.28 [95% CI-0.68 ,1.24] |
|  | Excluding studies with high overall risk of bias | MD -14.65 [95% CI -19.18, -10.12] |
|  | Using SMD as effect measure | SMD -1.47 [95% CI-1.97 , -0.96] |
| Hf-rTMS-LDLPFC | Primary analysis | MD -0.15 [95% CI-1.11,0.81] |
|  | Excluding studies with high overall risk of bias | MD -10.20 [95% CI -22.03,1.63] |
|  | Using SMD as effect measure | SMD -0.68 [95% CI-1.30, -0.06] |
| tONS-100Hz | Primary analysis | MD -0.48 [95% CI-1.45,0.50] |
|  | Excluding studies with high overall risk of bias | MD -1.42 [95% CI -5.02,2.18] |
|  | Using SMD as effect measure | SMD -0.26 [95% CI-0.85,0.33] |
| c-tDCS-Oz+a-tDCS-Cz | Primary analysis | MD 0.11 [95% CI-0.65,0.87] |
|  | Excluding studies with high overall risk of bias | MD -0.90 [95% CI -6.66,4.86] |
|  | Using SMD as effect measure | SMD -0.55 [95% CI-1.19 , 0.09] |
| tONS-2/100Hz | Primary analysis | MD -0.42 [95% CI-1.41, 0.57] |
|  | Excluding studies with high overall risk of bias | MD 0.95 [95% CI -2.10 ,4.00] |
|  | Using SMD as effect measure | SMD 0.17 [95% CI-0.42,0.77] |
| tONS-2Hz | Primary analysis | MD -1.03 [95% CI-2.10 , 0.04] |
|  | Excluding studies with high overall risk of bias | MD 2.63 [95% CI 0.01,5.25] |
|  | Using SMD as effect measure | SMD 0.48 [95% CI-0.11,1.08] |
| c-M1-tDCS-C4 | Primary analysis | MD -1.34 [95% CI-2.25, -0.43] |
|  | Excluding studies with high overall risk of bias | NA |
|  | Using SMD as effect measure | SMD -1.16 [95% CI-1.91, -0.40] |
| c-S1-tDCS-CP4 | Primary analysis | MD -1.03 [95% CI-2.10, 0.04] |
|  | Excluding studies with high overall risk of bias | NA |
|  | Using SMD as effect measure | SMD -1.16 [95% CI-1.91, -0.40] |
| Lf-rTMS-Cz | Primary analysis | MD 0.59 [95% CI-0.37,1.55] |
|  | Excluding studies with high overall risk of bias | NA |
|  | Using SMD as effect measure | SMD -0.11 [95% CI-0.87,0.64] |

1. **HIT-6**

| **Treatment** | **Analysis** | **Result** |
| --- | --- | --- |
| tONS-2Hz | Primary analysis | MD -0.87 [95% CI-1.75 , 0.01] |
|  | Excluding studies with high overall risk of bias | MD -2.19 [95%-CI -5.63, 1.25] |
|  | Using SMD as effect measure | SMD -0.33 [95% CI -0.93,0.26] |
| c-tDCS-C4 | Primary analysis | MD -0.86 [95% CI-1.60 , -0.12] |
|  | Excluding studies with high overall risk of bias | NA |
|  | Using SMD as effect measure | -0.13 [95% CI -0.55,0.28] |
| tONS-2/100Hz | Primary analysis | MD -0.55 [95% CI-1.42 , 0.32] |
|  | Excluding studies with high overall risk of bias | MD -6.37 [95%-CI -10.06, -2.68] |
|  | Using SMD as effect measure | SMD -0.97 [95% CI -1.57, -0.36] |
| a-tDCS-C3+c-tDCS-FP2 | Primary analysis | MD -0.23 [95% CI-0.96 , 0.49] |
|  | Excluding studies with high overall risk of bias | MD -4.00 [95%-CI -8.47, 0.47] |
|  | Using SMD as effect measure | SMD -0.66 [95% CI -1.42, 0.10] |
| a-tDCS-C4 | Primary analysis | MD -0.20 [95% CI-0.92 , 0.52] |
|  | Excluding studies with high overall risk of bias | NA |
|  | Using SMD as effect measure | SMD -0.10 [95% CI -0.51, 0.32] |
| Hf-rTMS-LDLPFC | Primary analysis | MD -0.04 [-95% CI 0.45, 0.38] |
|  | Excluding studies with high overall risk of bias | MD -9.20 [95%-CI -16.02, -2.38] |
|  | Using SMD as effect measure | SMD -0.99 [95% CI -1.77, -0.22] |
| tONS-100Hz | Primary analysis | MD -0.10 [95% CI-0.32 , 0.51] |
|  | Excluding studies with high overall risk of bias | MD -1.96 [95%-CI -5.96, 2.04] |
|  | Using SMD as effect measure | SMD -0.30 [95% CI -0.89, 0.30] |

**F.Pain intensity**

| **Treatment** | **Analysis** | **Result** |
| --- | --- | --- |
| Lf-rTMS-Cz | Primary analysis | MD -2.60 [95% CI -6.27, 1.07] |
|  | Excluding studies with high overall risk of bias | NA |
|  | Using SMD as effect measure | SMD 0.11 [95%-CI -2.31, 2.53] |
| tONS-2Hz | Primary analysis | MD -2.17 [95% CI -5.82, 1.48] |
|  | Excluding studies with high overall risk of bias | MD -1.20 [95% CI -3.14, 0.74] |
|  | Using SMD as effect measure | SMD -0.83 [95%-CI -3.20, 1.55] |
| STS-Afz | Primary analysis | MD -1.15 [95% CI -4.40, 2.11] |
|  | Excluding studies with high overall risk of bias | MD -4.08 [95% CI -6.80, -1.36] |
|  | Using SMD as effect measure | SMD -0.89 [95%-CI -2.82, 1.03] |
| c-M1-tDCS-C4 | Primary analysis | MD -0.77 [95% CI -4.39, 2.85] |
|  | Excluding studies with high overall risk of bias | NA |
|  | Using SMD as effect measure | SMD -3.41 [95%-CI -5.91, -0.90] |
| tONS-100Hz | Primary analysis | MD -0.53 [95% CI -4.12,3.05] |
|  | Excluding studies with high overall risk of bias | MD -0.95 [95% CI -2.91,1.01] |
|  | Using SMD as effect measure | SMD -0.65 [95%-CI -3.03,1.72] |
| VC-tDCS-OzCz | Primary analysis | MD -0.28 [95% CI -2.81, 2.25] |
|  | Excluding studies with high overall risk of bias | MD -0.18 [95% CI -1.47, 1.11] |
|  | Using SMD as effect measure | SMD -0.97 [95%-CI -2.42, 0.48] |
| a-tDCS-M1-C3 | Primary analysis | MD -0.19 [95% CI -3.14, 2.76] |
|  | Excluding studies with high overall risk of bias | MD -1.57 [95% CI -3.28, 0.13] |
|  | Using SMD as effect measure | SMD -0.63 [95%-CI -1.87, 0.60] |
| 25HztaVNS | Primary analysis | MD -0.15 [95% CI -3.40, 3.10] |
|  | Excluding studies with high overall risk of bias | NA |
|  | Using SMD as effect measure | SMD -1.03 [95%-CI -4.38, 2.31] |
| a-tDCS-DLPFC-F3 | Primary analysis | MD -0.09 [95% CI -3.67; 3.48] |
|  | Excluding studies with high overall risk of bias | MD 4.09 [95% CI 1.86; 6.32] |
|  | Using SMD as effect measure | SMD 1.23 [95%-CI -1.28; 3.74] |
| Hf-rTMS-LDLPFC | Primary analysis | MD -0.08 [95% CI -3.65; 3.49] |
|  | Excluding studies with high overall risk of bias | MD -2.80 [95% CI -5.01; -0.59] |
|  | Using SMD as effect measure | SMD -1.56 [95%-CI -4.00; 0.89] |
| tONS-2/100Hz | Primary analysis | MD 0.09 [95% CI -3.48; 3.66] |
|  | Excluding studies with high overall risk of bias | MD -1.22 [95% CI -3.15; 0.71] |
|  | Using SMD as effect measure | SMD -0.84 [95%-CI -3.22; 1.53] |
| PMES | Primary analysis | MD 0.11 [95% CI -2.73; 2.96] |
|  | Excluding studies with high overall risk of bias | MD -4.68 [95% CI -6.62; -2.74] |
|  | Using SMD as effect measure | SMD -1.90 [95%-CI -3.83; 0.03] |
| 1HztaVNS | Primary analysis | MD 0.74 [95% CI -1.78; 3.26] |
|  | Excluding studies with high overall risk of bias | MD -13.30 [95% CI -22.50; -4.10] |
|  | Using SMD as effect measure | SMD -0.75 [95%-CI -3.11; 1.61] |
| cS1-tDCS-CP4 | Primary analysis | MD 0.85 [95% CI -2.76; 4.45] |
|  | Excluding studies with high overall risk of bias | NA |
|  | Using SMD as effect measure | SMD -2.97 [95%-CI -5.45; -0.49] |
| c-tDCS-FC | Primary analysis | MD 1.84 [95% CI -1.83; 5.51] |
|  | Excluding studies with high overall risk of bias | MD -39.40 [95% CI -54.60; -24.20] |
|  | Using SMD as effect measure | SMD -1.30 [95%-CI -3.69; 1.09] |

**G. Drop-out rate**

| **Treatment** | **Analysis** | **Result** |
| --- | --- | --- |
| a-tDCS-M1-C3 | Primary analysis | RR: 2.02 [0.35; 11.73] |
|  | Excluding studies with high overall risk of bias | NA |
|  | Using OR as effect measure | OR 2.36 [95% CI 0.32 – 17.63] |
| tONS-100Hz | Primary analysis | RR: 0.57 [0.30; 1.08] |
|  | Excluding studies with high overall risk of bias | RR 0.57 [95% CI 0.30 – 1.08] |
|  | Using OR as effect measure | OR 0.33 [95% CI 0.10 – 1.12] |
| tONS-2Hz | Primary analysis | RR: 0.57 [0.30; 1.08] |
|  | Excluding studies with high overall risk of bias | RR 0.57 [95% CI 0.30 – 1.08] |
|  | Using OR as effect measure | OR 0.33 [95% CI 0.10 – 1.12] |
| tONS-2/100Hz | Primary analysis | RR: 0.36 [0.16; 0.82] |
|  | Excluding studies with high overall risk of bias | RR 0.36 [95% CI 0.16 – 0.82] |
|  | Using OR as effect measure | OR 0.17 [95% CI 0.04 – 0.63] |
| a-tDCS-C3+c-tDCS-FP2 | Primary analysis | RR: 1.00 [0.29; 3.39] |
|  | Excluding studies with high overall risk of bias | RR 1.00 [95% CI 0.29 – 3.39] |
|  | Using OR as effect measure | OR 1.00 [95% CI 0.21 – 4.81] |
| Hf-rTMS-LDLPFC | Primary analysis | RR: 1.22 [0.36; 4.17] |
|  | Excluding studies with high overall risk of bias | RR 1.22 [95% CI 0.36 – 4.17] |
|  | Using OR as effect measure | OR 1.29 [95% CI 0.31 – 5.30] |
| a-tDCS-DLPFC-F3 | Primary analysis | RR: 0.77 [0.03; 18.21] |
|  | Excluding studies with high overall risk of bias | NA |
|  | Using OR as effect measure | OR 0.70 [95% CI 0.02 – 25.27] |
| VC-tDCS-OzCz | Primary analysis | RR: 0.10 [0.01; 1.63] |
|  | Excluding studies with high overall risk of bias | RR 0.10 [95% CI 0.01 – 1.63] |
|  | Using OR as effect measure | OR 0.06 [95% CI 0.00 – 1.29] |
| sTMS | Primary analysis | RR: 2.33 [0.62; 8.71] |
|  | Excluding studies with high overall risk of bias | RR 2.33 [95% CI 0.62 – 8.71] |
|  | Using OR as effect measure | OR 2.46 [95% CI 0.61 – 9.86] |
| Bi-nVNS | Primary analysis | RR: 0.82 [0.64; 1.05] |
|  | Excluding studies with high overall risk of bias | NA |
|  | Using OR as effect measure | OR 0.71 [95% CI 0.46 – 1.09] |
| Rt-nVNS | Primary analysis | RR: 0.75 [0.47; 1.21] |
|  | Excluding studies with high overall risk of bias | RR 0.75 [95% CI 0.47 – 1.21] |
|  | Using OR as effect measure | OR 0.53 [95% CI 0.19 – 1.51] |

**H.Analgesic**

| **Treatment** | **Analysis** | **Result** |
| --- | --- | --- |
| a-tDCS-C3+c-tDCS-FP2 | Primary analysis | MD -0.93 [95% CI -1.67 -0.20] |
|  | Excluding studies with high overall risk of bias | MD -7.80 [95% CI -18.46 – 2.86] |
|  | Using SMD as effect measure | SMD -0.42 [95% CI -0.97 – 0.14] |
| Bi-nVNS | Primary analysis | MD -0.66 [95% CI -1.17 -0.15] |
|  | Excluding studies with high overall risk of bias | NA |
|  | Using SMD as effect measure | SMD -0.15 [95% CI -0.36 – 0.07] |
| VC-tDCS-OzCz | Primary analysis | MD -0.51 [95% CI -1.03 0.01] |
|  | Excluding studies with high overall risk of bias | MD -0.10 [95% CI -0.43 – 0.23] |
|  | Using SMD as effect measure | SMD -0.06 [95% CI -0.72 – 0.59] |
| Lf-rTMS-Cz | Primary analysis | MD -0.20 [95% CI -0.73 0.34] |
|  | Excluding studies with high overall risk of bias | NA |
|  | Using SMD as effect measure | SMD 0.14 [95% CI -0.62 – 0.89] |
| c-tDCS-C4 | Primary analysis | MD -0.16 [95% CI -0.81 0.49] |
|  | Excluding studies with high overall risk of bias | MD -3.40 [95% CI -6.58 – -0.22] |
|  | Using SMD as effect measure | SMD -0.44 [95% CI -0.86 – -0.03] |
| PMES | Primary analysis | MD -0.02 [95% CI -0.52 0.48] |
|  | Excluding studies with high overall risk of bias | MD -4.05 [95% CI -5.79 – -2.31] |
|  | Using SMD as effect measure | SMD -0.92 [95% CI -1.30 – -0.53] |
| c-tDCS-FC | Primary analysis | MD 0.10 [95% CI -0.30 0.49] |
|  | Excluding studies with high overall risk of bias | MD -38.30 [95% CI -54.99 – -21.61] |
|  | Using SMD as effect measure | SMD -1.20 [95% CI -1.85 – -0.55] |
| a-tDCS-M1-C3 | Primary analysis | MD 0.11 [95% CI -0.42 0.65] |
|  | Excluding studies with high overall risk of bias | MD -2.59 [95% CI -6.49 – 1.31] |
|  | Using SMD as effect measure | SMD -0.27 [95% CI -0.64 – 0.10] |
| a-tDCS-C4 | Primary analysis | MD 0.18 [95% CI -0.56 0.91] |
|  | Excluding studies with high overall risk of bias | MD -1.00 [95% CI -3.81 – 1.81] |
|  | Using SMD as effect measure | SMD -0.13 [95% CI -0.55 – 0.29] |
| STS-Afz | Primary analysis | MD 0.24 [95% CI -0.09 0.58] |
|  | Excluding studies with high overall risk of bias | MD -3.88 [95% CI -5.85 – -1.91] |
|  | Using SMD as effect measure | SMD -0.76 [95% CI -1.16 – -0.36] |
| Hf-rTMS-LDLPFC | Primary analysis | MD -0.37 [95% CI -0.45 1.20] |
|  | Excluding studies with high overall risk of bias | MD -2.74 [95% CI -7.48 – 1.99] |
|  | Using SMD as effect measure | SMD -0.25 [95% CI -0.58 – 0.09] |

**I.Adverse events**

| **Treatment** | **Analysis** | **Result** |
| --- | --- | --- |
| sTMS | Primary analysis | RR: 1.51 [0.68; 3.33] |
|  | Excluding studies with high overall risk of bias | RR 1.51 [95% CI 0.68 – 3.33] |
|  | Using OR as effect measure | OR 1.59 [95% CI 0.65 – 3.86] |
| c-S1-tDCS-CP4 | Primary analysis | RR: 0.67 [0.13; 3.44] |
|  | Excluding studies with high overall risk of bias | NA |
|  | Using OR as effect measure | OR 0.62 [95% CI 0.09 – 4.34] |
| c-M1-tDCS-C4 | Primary analysis | RR: 1.00 [0.24; 4.18] |
|  | Excluding studies with high overall risk of bias | NA |
|  | Using OR as effect measure | OR 1.00 [95% CI 0.17 – 5.98] |
| a-tDCS-M1-C3 | Primary analysis | RR: 1.19 [0.69; 2.03] |
|  | Excluding studies with high overall risk of bias | RR 1.23 [95% CI 0.67 – 2.25] |
|  | Using OR as effect measure | OR 1.28 [95% CI 0.58 – 2.85] |
| Hf-rTMS-LDLPFC | Primary analysis | RR: 2.57 [1.05; 6.29]  64] |
|  | Excluding studies with high overall risk of bias | RR 2.39 [95% CI 0.93 – 6.14] |
|  | Using OR as effect measure | OR 5.79 [95% CI 1.24 – 27.09] |
| tONS-2/100Hz | Primary analysis | RR: 1.00 [0.02; 48.24] |
|  | Excluding studies with high overall risk of bias | NA |
|  | Using OR as effect measure | OR 1.00 [95% CI 0.02 – 52.63] |
| Rt-nVNS | Primary analysis | RR: 1.03 [0.65; 1.62] |
|  | Excluding studies with high overall risk of bias | RR 1.03 [95% CI 0.65 – 1.62] |
|  | Using OR as effect measure | 1.06 [95% CI 0.38 – 2.97] |
| Lf-rTMS-Cz | Primary analysis | RR: 1.55 [0.46; 5.22] |
|  | Excluding studies with high overall risk of bias | NA |
|  | Using OR as effect measure | OR 1.85 [95% CI 0.34 – 10.05] |
| Unilateral-nVNS | Primary analysis | RR: 1.05 [0.77; 1.45] |
|  | Excluding studies with high overall risk of bias | RR 1.05 [95% CI 0.77 – 1.45] |
|  | Using OR as effect measure | OR 1.09 [95% CI 0.64 – 1.85] |
| tONS-100Hz | Primary analysis | RR: 1.00 [0.02; 48.24] |
|  | Excluding studies with high overall risk of bias | NA |
|  | Using OR as effect measure | OR 1.00 [95% CI 0.02 – 52.63] |
| tONS-2Hz | Primary analysis | RR: 3.00 [0.13; 69.79] |
|  | Excluding studies with high overall risk of bias | NA |
|  | Using OR as effect measure | OR 3.14 [95% CI 0.12 – 81.35] |
| Bi-nVNS | Primary analysis | RR: 0.83 [0.66; 1.03] |
|  | Excluding studies with high overall risk of bias | NA |
|  | Using OR as effect measure | OR 0.69 [95% CI 0.45 – 1.06] |

**eTable 9. Risk of bias of each domain for each study.**

| **Study** | **Random sequence generation** | **Allocation concealment** | **Blinding of participants and personnel** | **Blinding of outcome assessment** | **Incomplete outcome data** | **Selective reporting** | **Other Bias** |
| --- | --- | --- | --- | --- | --- | --- | --- |
| Teepker 2010 | low | low | high | low | low | low | unclear |
| Granato 2019 | unclear | low | low | low | high | low | unclear |
| Amin 2020 | low | low | low | unclear | unclear | low | low |
| Misra 2013 | low | low | low | low | low | low | unclear |
| Conforto 2014 | low | low | low | low | low | low | unclear |
| Lipton 2010 | low | low | low | low | unclear | low | low |
| Pohl 2021 | low | low | low | low | unclear | low | high |
| Hodaj 2022 | low | low | low | low | low | low | unclear |
| Dalla 2020 | low | low | low | low | low | low | low |
| DaSilva 2023 | low | low | low | unclear | low | low | unclear |
| Rahimi 2020 | low | low | low | low | unclear | low | high |
| Antal 2011 | unclear | unclear | low | low | low | low | high |
| Rocha 2015 | low | low | unclear | unclear | low | low | high |
| Andrade 2017 | low | low | low | low | high | low | low |
| Grazzi 2020 | low | low | low | unclear | low | low | high |
| Şirin 2021 | low | unclear | low | low | low | low | low |
| Auvichayapat 2012 | unclear | low | low | low | unclear | low | high |
| Aksu 2023 | low | low | low | low | low | low | high |
| Najib 2022 | low | low | low | unclear | unclear | low | low |
| Diener 2019 | low | low | low | unclear | low | low | unclear |
| Zhang 2021 | low | unclear | low | unclear | low | low | unclear |
| Silberstein 2016 | low | low | low | unclear | high | low | low |
| Liu 2017 | low | low | low | low | low | low | high |
| Schoenen 2013 | low | low | low | unclear | low | low | high |
| Deng 2020 | low | low | low | high | unclear | unclear | low |
| Juan 2017 | unclear | low | low | low | unclear | unclear | low |
| Straube 2015 | low | low | low | low | low | low | unclear |
| Song 2025 | low | low | low | low | low | low | low |

**eTable10A: Quality of evidence for primary outcome: changes in monthly migraine days.**

| **comparison** | **Direct evidence** |  | **indirect evidence** | **Network meta analysis** |  |
| --- | --- | --- | --- | --- | --- |
|  | Meandifference[95%CI] | Certainty of direct evidence | Certainty of indirect evidence | Risk difference [95%CI] | Overall quality of evidence |
| Hf-rTMS-LDLPFC  vs  Sham/Control | -1.00 [-2.94,0.94] | ⨁⨁⨁◯  Moderate | / | 0.37[3.01,2.26] | ⨁⨁⨁◯  Moderate |
| Lf-rTMS-Cz  vs  Sham/Control | 0.32 [-6.30,6.94] | ⨁⨁⨁◯  Moderate | / | 0.04[2.60,2.67] | ⨁⨁⨁◯  Moderate |
| VC-tDCS-OzCz  vs  Sham/Control | -2.00 [-2.36, -1.63] | ⨁⨁◯◯  Low | / | -1.75[-3.37,0.13] | ⨁⨁◯◯  Low |
| a-tDCS-C3+c-tDCS-FP2  vs  Sham/Control | -5.20 [-9.89, -0.51] | ⨁⨁⨁⨁  High | / | 0.86[.275,1.02] | ⨁⨁⨁⨁  High |
| a-tDCS-C4  vs  Sham/Control | 2.60 [-0.49,5.69] | ⨁⨁⨁⨁  High | ⨁⨁⨁⨁  High | 0.35[<2.21.2.91>] | ⨁⨁⨁◯  Moderate |
| c-tDCS-C4  vs  Sham/Control | 3.20[0.08,6.32] | ⨁⨁⨁⨁  High | ⨁⨁⨁⨁  High | 0.43[2.13,2.99] | ⨁⨁⨁◯  Moderate |
| STS  vs  Sham/Control | -2.38 [-3.94, -0.82] | ⨁⨁⨁⨁  High | / | 0.89[3.00,1.21] | ⨁⨁⨁◯  Moderate |
| PMES  vs  Sham/Control | -2.87 [-4.43, -1.31] | ⨁⨁⨁◯  Moderate | ⨁⨁◯◯  Low | 0.67[2.77,1.42] | ⨁◯◯◯  Very low |
| a-tDCS-C3/C4+c-tDCS-FP2  vs  Sham/Control | -4.10 [-7.76, -0.44] | ⨁⨁⨁⨁  High | / | 0.86[2.75,1.02] | ⨁⨁⨁⨁  High |
| 1hz taVNS  vs  Sham/Control | -1.70 [-2.94, -0.46] | ⨁⨁⨁◯  Moderate | / | 0.73[3.32,1.85] | ⨁⨁⨁◯  Moderate |
| VC-tDCS-OzCz  vs  Hf-rTMS-LDLPFC |  |  |  | 1.38[4.47,1.72] | ⨁⨁◯◯  Low |
| PMSE  vs  VC-tDCS-OzCz |  |  |  | 1.07[1.58,3.73] | ⨁◯◯◯  Very low |
| PMSE  vs  c-tDCS-C4 |  |  |  | 1.10[4.41,2.21] | ⨁⨁◯◯  Low |

**eTable10B: Quality of evidence for primary outcome: changes in drop out rate**

| **comparison** | **Direct evidence** |  | **indirect evidence** | **Network meta analysis** |  |
| --- | --- | --- | --- | --- | --- |
|  | **Risk difference [95%CI]** | Certainty of direct evidence | Certainty of indirect evidence | **Risk difference [95%CI]** | **Overall quality of evidence** |
| HF-rTMS-LDLPFC  vs  Sham/Control | 1.22 (0.36, 4.17) | ⨁⨁⨁◯  Moderate |  | 0.82 (0.24, 2.79) | ⨁⨁⨁◯  Moderate |
| HF-rTMS-LDLPFC  vs  STMS |  |  |  | 0.52 (0.09, 3.17) | ⨁⨁◯◯  Low |
| HF-rTMS-LDLPFC  vs  a-tDCS-C3+c-tDCS-FP2 |  |  |  | 0.82 (0.14, 4.63) | ⨁⨁◯◯  Low |
| HF-rTMS-LDLPFC  Vs  VC-tDCS-OzCz |  |  |  | 0.08 (0.00, 1.73) | ⨁⨁◯◯  Low |
| HF-rTMS-LDLPFC  vs  a-tDCS-M1-C3 |  |  |  | 0.60 (0.07, 5.16) | ⨁⨁◯◯  Low |
| HF-rTMS-LDLPFC  Vs  a-tDCS-DLPFC-F3 |  |  |  | 0.63 (0.02, 18.75) | ⨁⨁◯◯  Low |
| HF-rTMS-LDLPFC  Vs  Bi-nVNS |  |  |  | 0.67 (0.19, 2.35) | ⨁⨁◯◯  Low |
| HF-rTMS-LDLPFC  Vs  Rt-nVNS |  |  |  | 0.62 (0.16, 2.30) | ⨁⨁◯◯  Low |
| HF-rTMS-LDLPFC  vs  tONS-2Hz |  |  |  | 0.47 (0.12, 1.86) | ⨁⨁◯◯  Low |
| HF-rTMS-LDLPFC  vs  tONS-100Hz |  |  |  | 0.47 (0.12, 1.86) | ⨁⨁◯◯  Low |
| HF-rTMS-LDLPFC  vs  tONS-2/100Hz |  |  |  | 0.29 (0.07, 1.29) | ⨁⨁◯◯  Low |
| Sham/Control  Vs  sTMS | 2.33 (0.62, 8.71) | ⨁⨁⨁◯  Moderate |  | 0.43 (0.11, 1.60) | ⨁⨁⨁◯  Moderate |
| Sham/Control  Vs  a-tDCS-C3+c-tDCS-FP2 | 1.00 (0.29, 3.39) | ⨁⨁⨁◯  Moderate |  | 1.00 (0.29, 3.39) | ⨁⨁⨁◯  Moderate |
| Sham/Control  vs  VC-tDCS-OzCz | 0.10 (0.01, 1.63) | ⨁⨁⨁◯  Moderate |  | 0.10 (0.01, 1.63) | ⨁⨁⨁◯  Moderate |
| Sham/Control  vs  a-tDCS-M1-C3 |  |  |  | 0.49 (0.09, 2.87) | ⨁⨁◯◯  Low |
| Sham/Control  vs  a-tDCS-DLPFC-F3 | 0.78 (0.02, 30.30) | ⨁⨁⨁◯  Moderate |  | 0.77 (0.03, 18.21) | ⨁⨁⨁◯  Moderate |
| Sham/Control  Vs  Bi-nVNS | 0.82 (0.64, 1.05) | ⨁⨁⨁◯  Moderate |  | 0.82 (0.64, 1.05) | ⨁⨁⨁◯  Moderate |
| Sham/Control  vs  Rt-nVNS | 0.75 (0.47, 1.21) | ⨁⨁⨁◯  Moderate |  | 0.75 (0.47, 1.21) | ⨁⨁⨁◯  Moderate |
| Sham/Control  vs  tONS-2Hz | 0.57 (0.30, 1.08) | ⨁⨁⨁◯  Moderate |  | 0.57 (0.30, 1.08) | ⨁⨁⨁◯  Moderate |
| Sham/Control  Vs  tONS-100Hz | 0.57 (0.30, 1.08) | ⨁⨁⨁◯  Moderate |  | 0.57 (0.30, 1.08) | ⨁⨁⨁◯  Moderate |
| Sham/Control  vs  tONS-2/100Hz | 0.36 (0.16, 0.82) | ⨁⨁⨁◯  Moderate |  | 0.36 (0.16, 0.82) | ⨁⨁⨁◯  Moderate |
| sTMS  vs  a-tDCS-C3+c-tDCS-FP2 |  |  |  | 0.43 (0.07, 2.59) | ⨁⨁◯◯  Low |
| sTMS  vs  VC-tDCS-OzCz |  |  |  | 0.04 (0.00, 0.94) | ⨁⨁◯◯  Low |
| sTMS  vs  a-tDCS-M1-C3 |  |  |  | 0.87 (0.10, 7.80) | ⨁⨁◯◯  Low |
| sTMS  vs  a-tDCS-DLPFC-F3 |  |  |  | 0.33 (0.01, 10.15) | ⨁⨁◯◯  Low |
| sTMS  vs  Bi-nVNS |  |  |  | 0.35 (0.09, 1.35) | ⨁⨁◯◯  Low |
| sTMS  Vs  Rt-nVNS |  |  |  | 0.32 (0.08, 1.31) | ⨁⨁◯◯  Low |
| sTMS  vs  tONS-2Hz |  |  |  | 0.24 (0.06, 1.06) | ⨁⨁◯◯  Low |
| sTMS  Vs  tONS-100Hz |  |  |  | 0.24 (0.06, 1.06) | ⨁⨁◯◯  Low |
| sTMS  vs  tONS-2/100Hz |  |  |  | 0.15 (0.03, 0.73) | ⨁⨁◯◯  Low |
| VC-tDCS-OzCz  vs  a-tDCS-M1-C3 |  |  |  | 0.05 (0.00, 1.34) | ⨁⨁◯◯  Low |
| VC-tDCS-OzCz  vs  a-tDCS-DLPFC-F3 |  |  |  | 0.10 (0.00, 2.11) | ⨁⨁◯◯  Low |
| VC-tDCS-OzCz  Vs  Bi-nVNS |  |  |  | 0.12 (0.01, 2.01) | ⨁⨁◯◯  Low |
| VC-tDCS-OzCz  vs  Rt-nVNS |  |  |  | 0.13 (0.01, 2.26) | ⨁⨁◯◯  Low |
| VC-tDCS-OzCz vs tONS-2Hz |  |  |  | 0.18 (0.01, 3.07) | ⨁⨁◯◯  Low |
| VC-tDCS-OzCz  Vs  tONS-100Hz |  |  |  | 0.18 (0.01, 3.07) | ⨁⨁◯◯  Low |
| VC-tDCS-OzCz  vs  tONS-2/100Hz |  |  |  | 0.28 (0.02, 5.16) | ⨁⨁◯◯  Low |
| 1. tDCS-M1-C3   Vs  a-tDCS-DLPFC-F3 | 0.38 (0.02, 6.29) | ⨁⨁⨁◯  Moderate |  | 0.38 (0.02, 6.21) | ⨁⨁⨁◯  Moderate |
| a-tDCS-M1-C3  vs  Bi-nVNS |  |  |  | 0.41 (0.07, 2.40) | ⨁⨁◯◯  Low |
| 1. tDCS-M1-C3   vs  Rt-nVNS |  |  |  | 0.37 (0.06, 2.30) | ⨁⨁◯◯  Low |
| a-tDCS-M1-C3  vs  tONS-2Hz |  |  |  | 0.28 (0.04, 1.83) | ⨁⨁◯◯  Low |
| 1. tDCS-M1-C3   Vs  tONS-100Hz |  |  |  | 0.28 (0.04, 1.83) | ⨁⨁◯◯  Low |
| 1. tDCS-M1-C3   Vs  tONS-2/100Hz |  |  |  | 0.18 (0.03, 1.23) | ⨁⨁◯◯  Low |
| 1. tDCS-DLPFC-F3   Vs  Bi-nVNS |  |  |  | 0.93 (0.04, 22.36) | ⨁⨁◯◯  Low |
| 1. tDCS-DLPFC-F3   vs  Rt-nVNS |  |  |  | 0.98 (0.04, 24.16) | ⨁⨁◯◯  Low |
| 1. tDCS-DLPFC-F3   vs  tONS-2Hz |  |  |  | 0.75 (0.03, 18.88) | ⨁⨁◯◯  Low |
| 1. tDCS-DLPFC-F3   Vs  tONS-100Hz |  |  |  | 0.75 (0.03, 18.88) | ⨁⨁◯◯  Low |
| 1. tDCS-DLPFC-F3   Vs  tONS-2/100Hz |  |  |  | 0.47 (0.02, 12.33) | ⨁⨁◯◯  Low |
| Bi-nVNS  Vs  Rt-nVNS |  |  |  | 0.75 (0.20, 2.79) | ⨁⨁◯◯  Low |
| Bi-nVNS  Vs  tONS-2Hz |  |  |  | 0.57 (0.14, 2.27) | ⨁⨁◯◯  Low |
| Bi-nVNS  vs  tONS-100Hz |  |  |  | 0.57 (0.14, 2.27) | ⨁⨁◯◯  Low |
| Bi-nVNS  vs  tONS-2/100Hz |  |  |  | 0.36 (0.08, 1.57) | ⨁⨁◯◯  Low |
| Rt-nVNS  vs  tONS-2Hz |  |  |  | 0.76 (0.34, 1.68) | ⨁⨁◯◯  Low |
| Rt-nVNS  Vs  tONS-100Hz |  |  |  | 0.76 (0.34, 1.68) | ⨁⨁◯◯  Low |
| Rt-nVNS  vs  tONS-2/100Hz |  |  |  | 0.48 (0.18, 1.24) | ⨁⨁◯◯  Low |
| tONS-2Hz  vs  tONS-100Hz | 1.00 (0.46, 2.19) | ⨁⨁⨁◯  Moderate |  | 1.00 (0.46, 2.19) | ⨁⨁⨁◯  Moderate |
| tONS-2Hz  vs  tONS-2/100Hz | 0.62 (0.24, 1.61) | ⨁⨁⨁◯  Moderate |  | 0.62 (0.24, 1.61) | ⨁⨁⨁◯  Moderate |
| tONS-100Hz  vs  tONS-2/100Hz | 0.62 (0.24, 1.61) | ⨁⨁⨁◯  Moderate |  | 0.62 (0.24, 1.61) | ⨁⨁⨁◯  Moderate |

We evaluated the GRADE ratings according to the rationale of the articles published in the BMJ [1] and the Lancet [2]

**References:**

[1]Puhan MA, Schunemann HJ, Murad MH, Li T, Brignardello-Petersen R, Singh JA, et al. A GRADE Working Group approach for rating the quality of treatment effect estimates from network meta-analysis. Bmj 2014;349:g5630.

[2]Cipriani A, Furukawa TA, Salanti G, Chaimani A, Atkinson LZ, Ogawa Y, et al. Comparative efficacy and acceptability of 21 antidepressant drugs for the acute treatment of adults with major depressive disorder: a systematic review and network meta-analysis. Lancet 2018.

**eTable 11. Prediction intervals for key intervention-versus-sham/control comparisons**

Prediction intervals are summarized for key comparisons requested during peer review. This table is not intended to reproduce the full league table or all possible network contrasts.

**A. Monthly migraine days (MMD)**

| **Intervention vs Sham/Control** | **Effect (MD)** | **95% CI** | **SE** | **95% PI** |
| --- | --- | --- | --- | --- |
| VC-tDCS-OzCz | −1.75 | −3.37 to −0.13 | 0.827 | −5.00 to 1.50 |
| STS-Afz | −0.89 | −3.00 to 1.21 | 1.074 | −4.41 to 2.63 |
| a-tDCS-C3+c-tDCS-FP2 | −0.86 | −2.75 to 1.02 | 0.962 | −4.25 to 2.53 |
| 1Hz_taVNS | −0.73 | −3.32 to 1.85 | 1.319 | −4.55 to 3.09 |
| PMES | −0.67 | −2.77 to 1.42 | 1.069 | −4.18 to 2.84 |
| a-tDCS-C4 | −0.35 | −2.91 to 2.21 | 1.306 | −4.16 to 3.46 |
| Hf-rTMS-LDLPFC | −0.37 | −3.01 to 2.26 | 1.344 | −4.23 to 3.49 |
| Bi-nVNS | −0.13 | −2.67 to 2.41 | 1.296 | −3.92 to 3.66 |
| Lf-rTMS-Cz | −0.04 | −2.67 to 2.60 | 1.344 | −3.90 to 3.82 |

For MMD, 95% prediction intervals were derived using the common between-study variance τ² = 2.066 estimated for the MMD network.

**B. Attack frequency**

| **Intervention vs Sham/Control** | **Effect (MD)** | **95% CI** | **SE** | **95% PI** |
| --- | --- | --- | --- | --- |
| PMES | −1.49 | −3.32 to 0.33 | 0.931 | −3.32 to 0.34 |
| a-tDCS-M1-C3 | −1.38 | −3.20 to 0.44 | 0.929 | −3.20 to 0.44 |
| c-tDCS-FC | −1.19 | −2.69 to 0.30 | 0.763 | −2.69 to 0.31 |
| Lf-rTMS-Cz | −0.78 | −2.55 to 0.98 | 0.901 | −2.55 to 0.99 |
| c-M1-tDCS-C4 | −0.50 | −2.08 to 1.08 | 0.806 | −2.08 to 1.08 |
| STS-Afz | −0.44 | −1.90 to 1.02 | 0.745 | −1.90 to 1.02 |
| c-S1-tDCS-CP4 | −0.42 | −2.01 to 1.16 | 0.809 | −2.01 to 1.17 |
| Hf-rTMS-LDLPFC | −0.02 | −1.30 to 1.26 | 0.653 | −1.30 to 1.26 |

For attack frequency, τ² ≈ 6.82 × 10⁻⁷; therefore, prediction intervals are almost identical to the corresponding confidence intervals.

Abbreviations and calculation: CI = confidence interval; MD = mean difference; MMD = monthly migraine days; PI = prediction interval; SE = standard error; τ² = between-study variance. *SE was back-calculated from the 95% CI as (upper limit − lower limit)/(2 × 1.96). Prediction intervals were calculated as MD ± 1.96 × √(SE² + τ²). The null value for MD is 0; all summarized intervals cross the null.

**eFigure 1A-I: network structure**

The lines connecting the nodes represent direct comparisons observed in different clinical trials. The size of each circle is proportionate to the number of participants who received a specific treatment. The thickness of the lines is proportional to the number of trials that are interconnected to the network.

**(A) Migraine days**


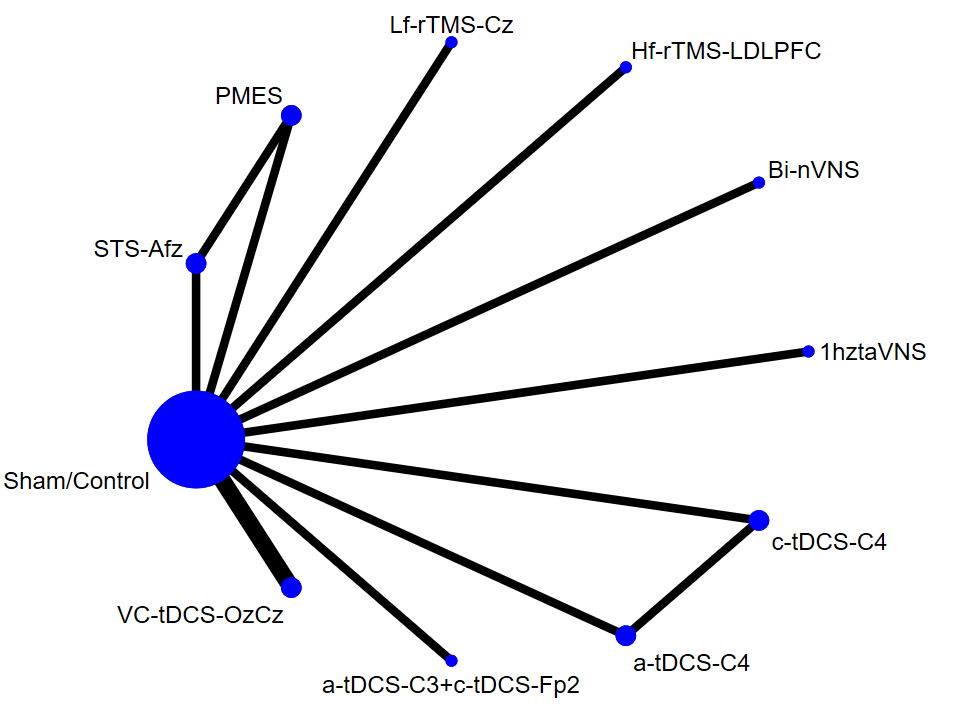


**(B) Attack frequency**


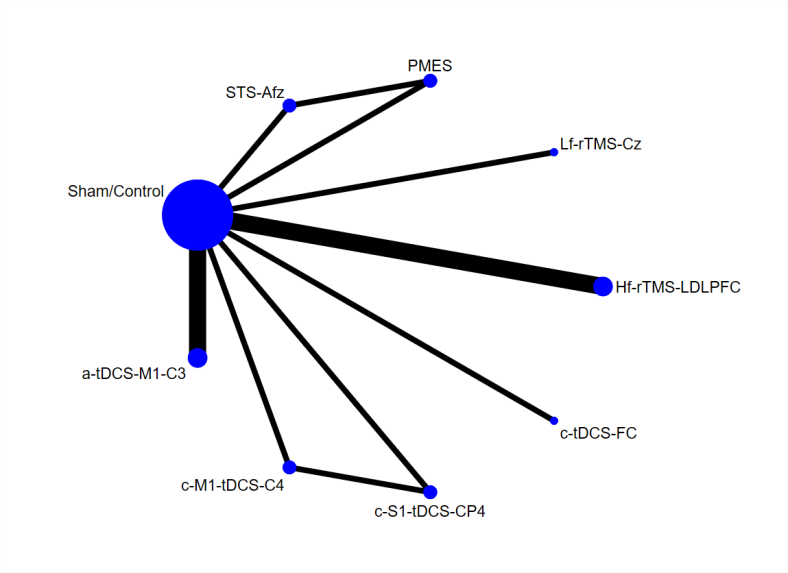


**(C) Responder rate**


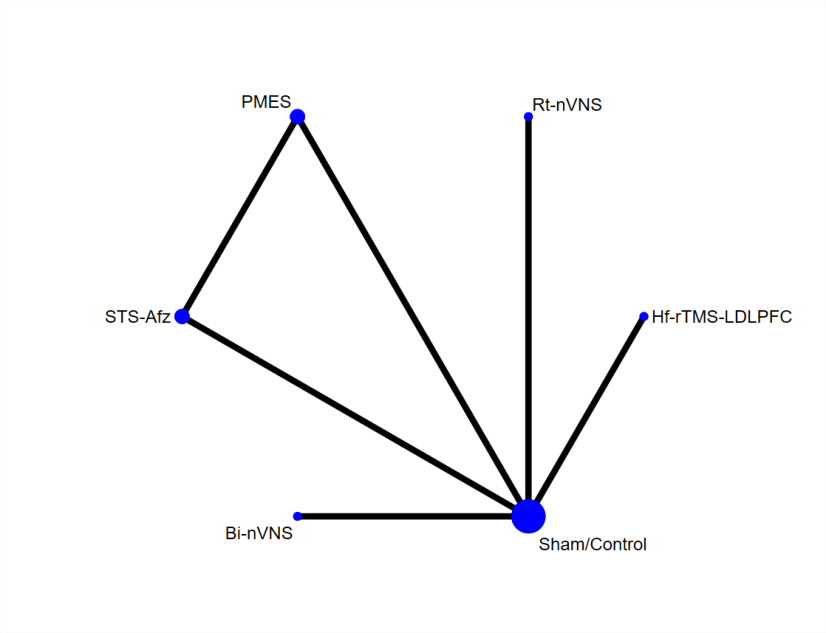


**(D) Duration**


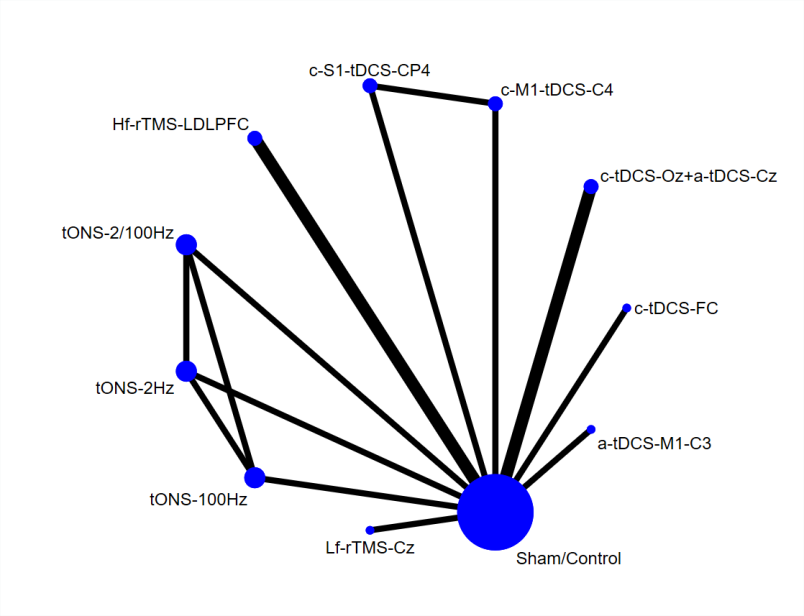


**(E)HIT-6**


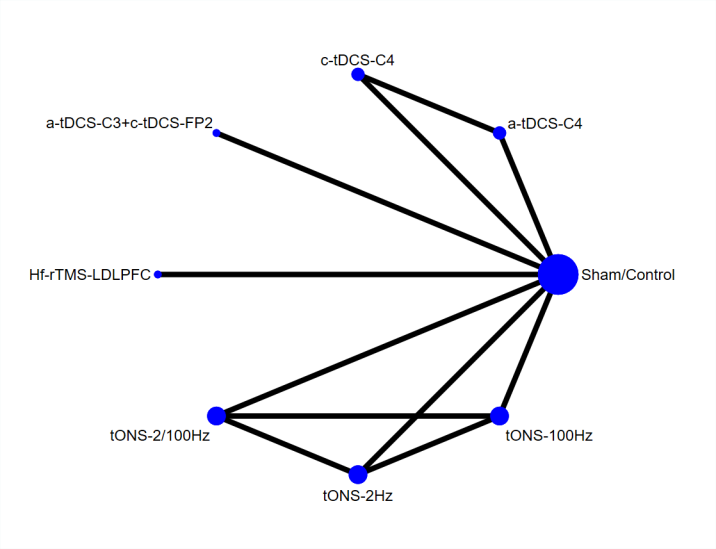


**(F) Pain intensity**


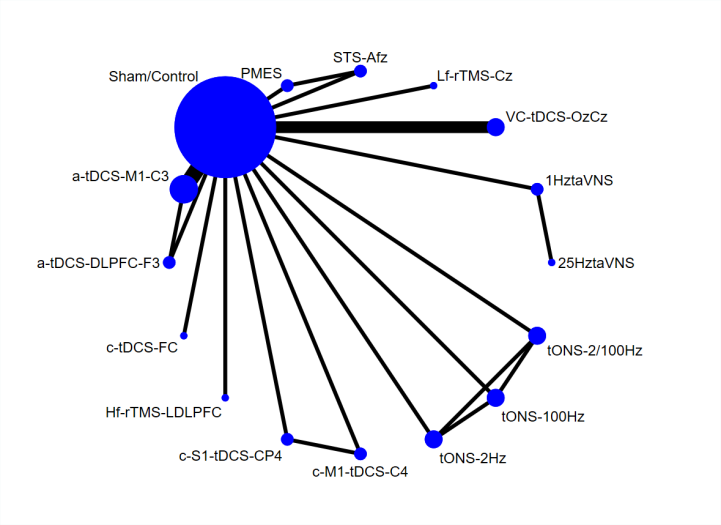


**(G) Drop-out rate**


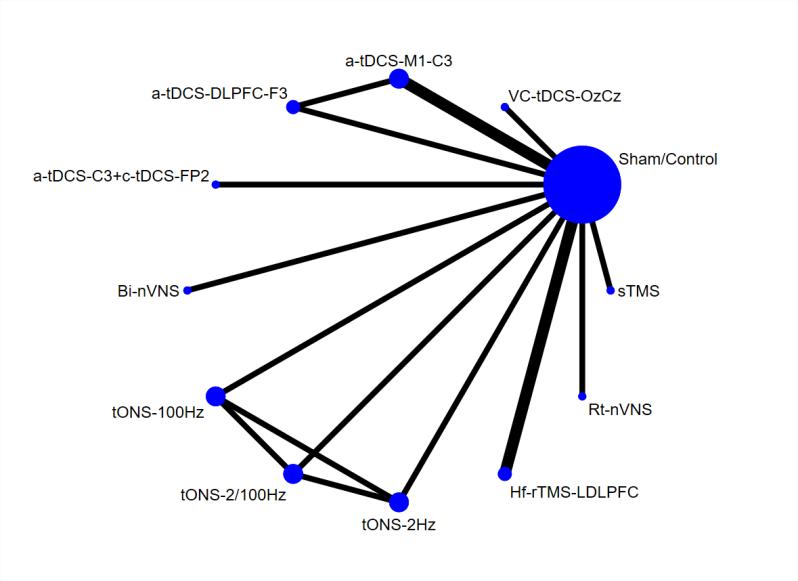


**(H) Analgesic**

.
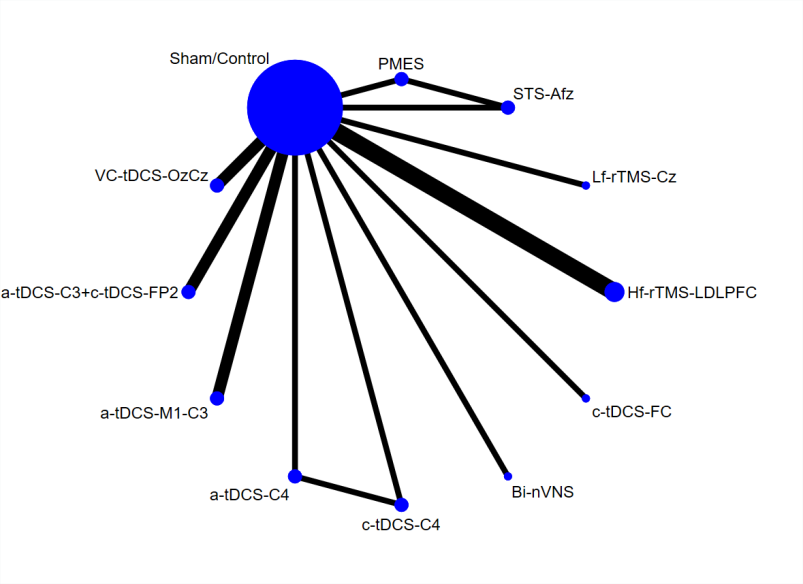


**(I)Adverse events**


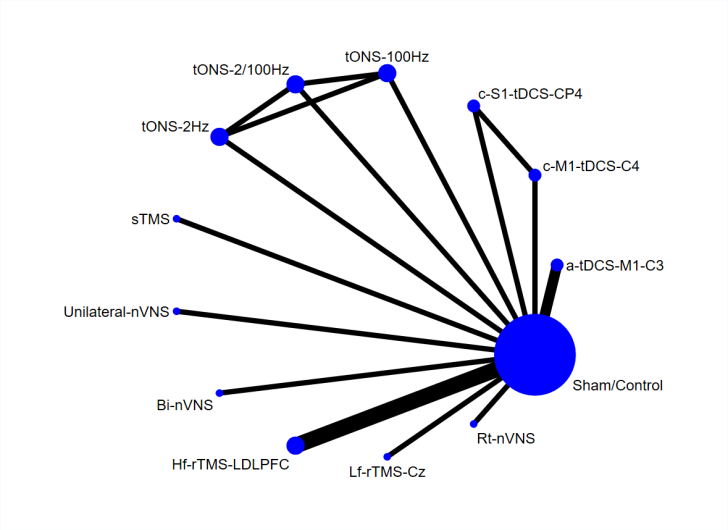


**eFigure 2A-I:Main results of forest map**

When the effect size is less than zero, as presented by the mean difference, the treatment under study resulted in a greater reduction in the number of migraine days compared to the sham control.

1. **Migraine days**


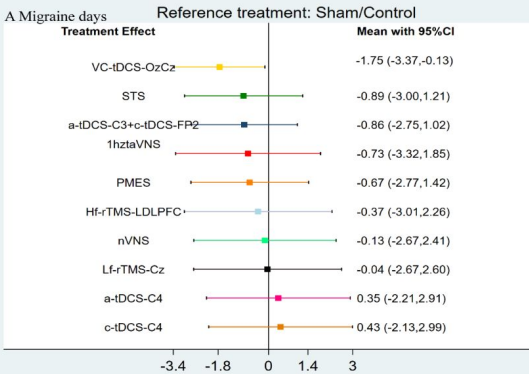


**(B) Attack frequency**


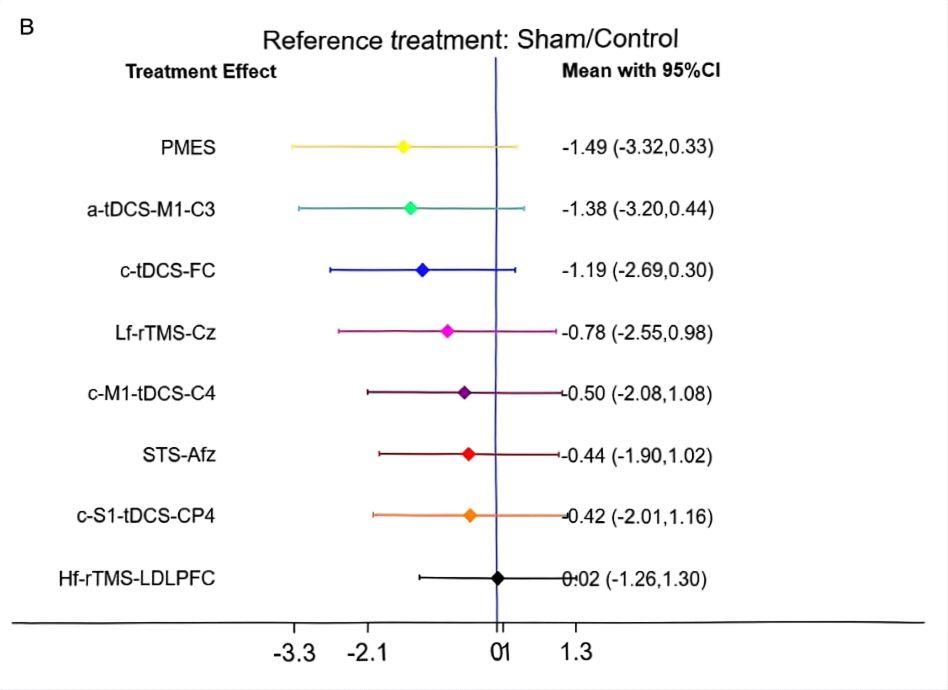


**(C) Responder rate**


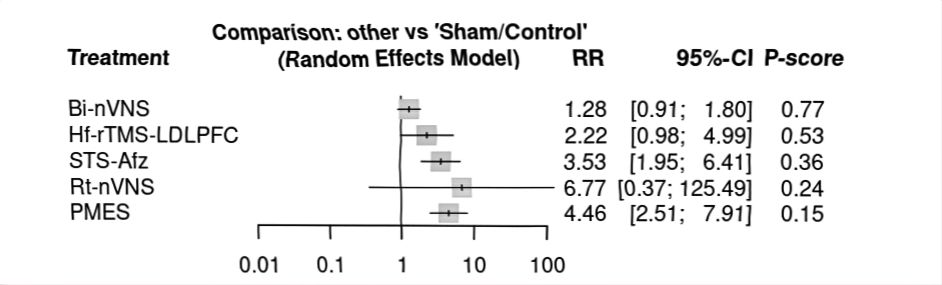


**(D) Duration**


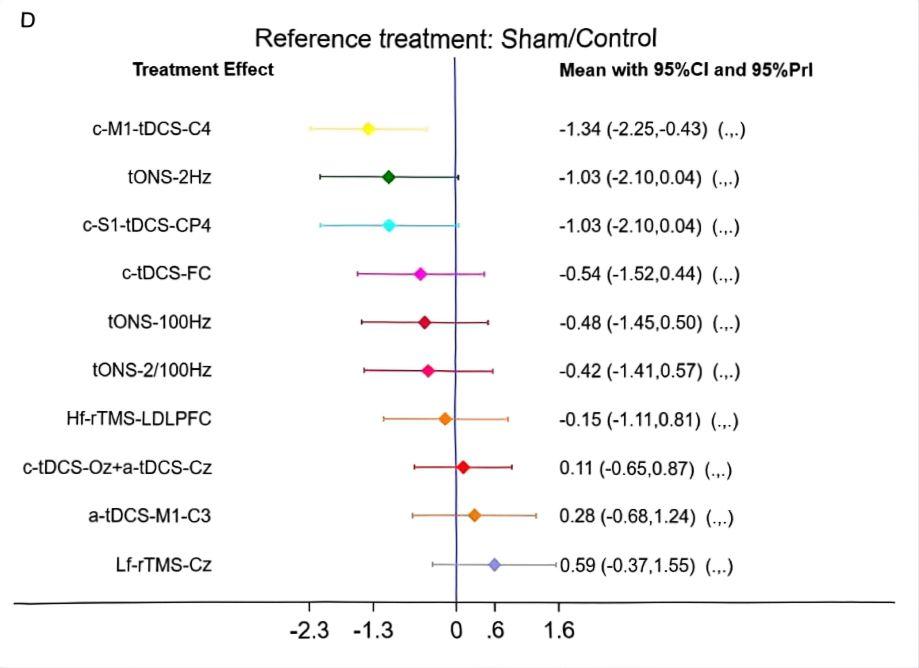


**(E)HIT-6**


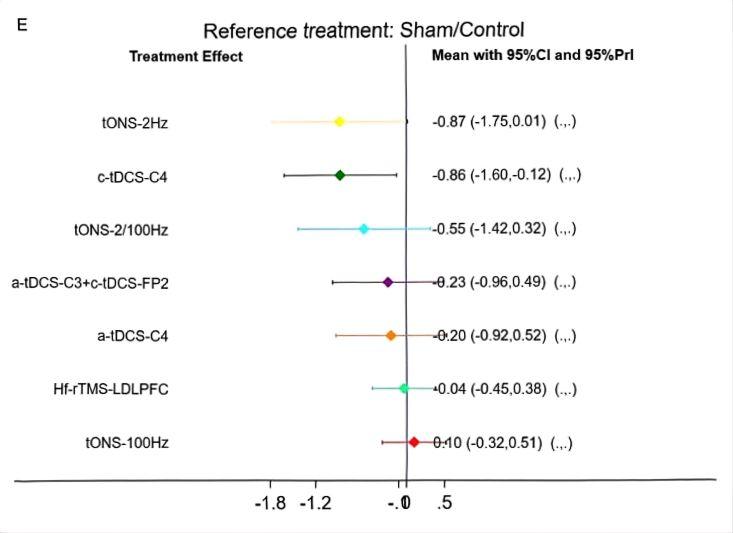


**(F) Pain intensity**


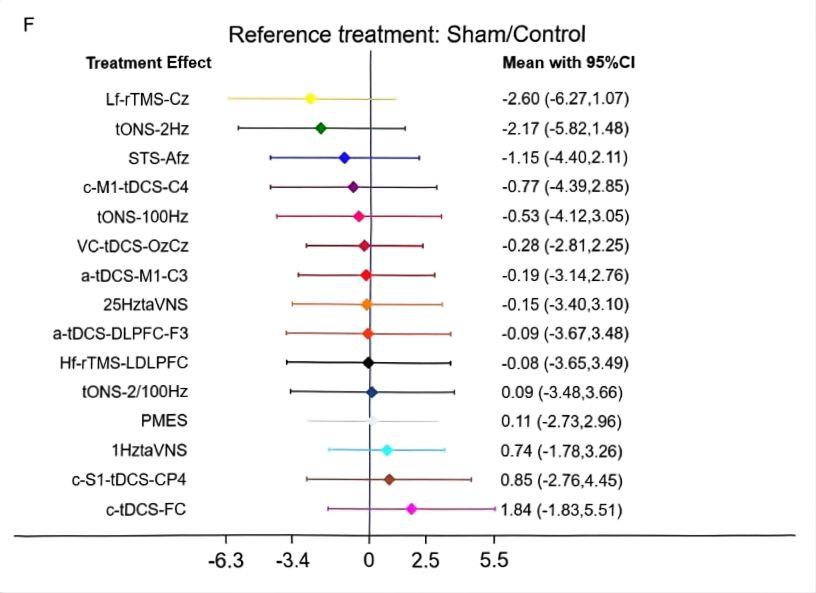


**(G) Drop-out rate**


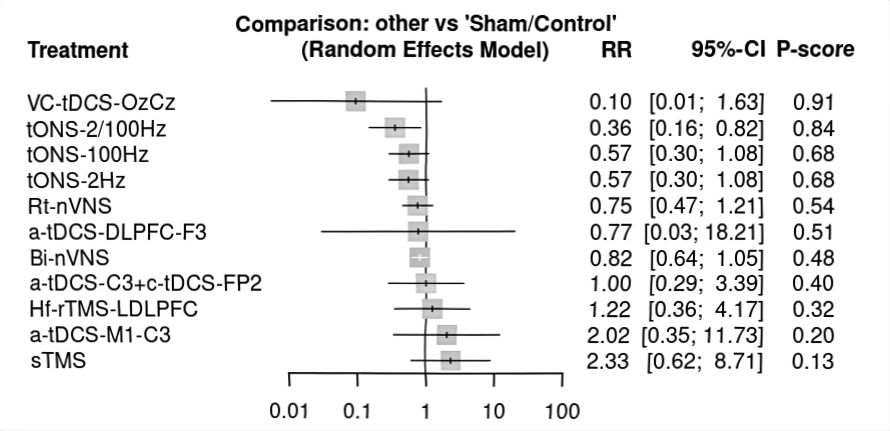


**(H) Analgesic**


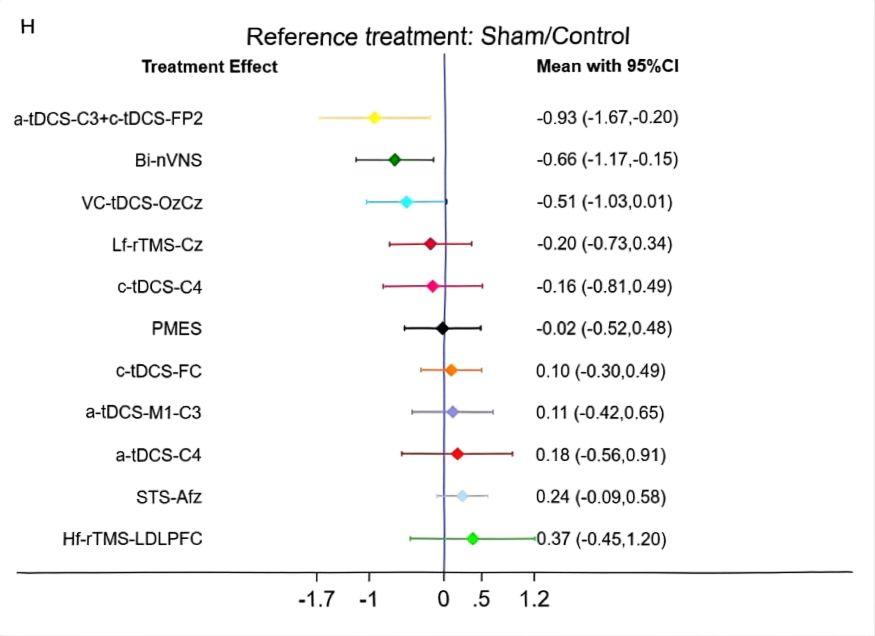


**(I)Adverse events**


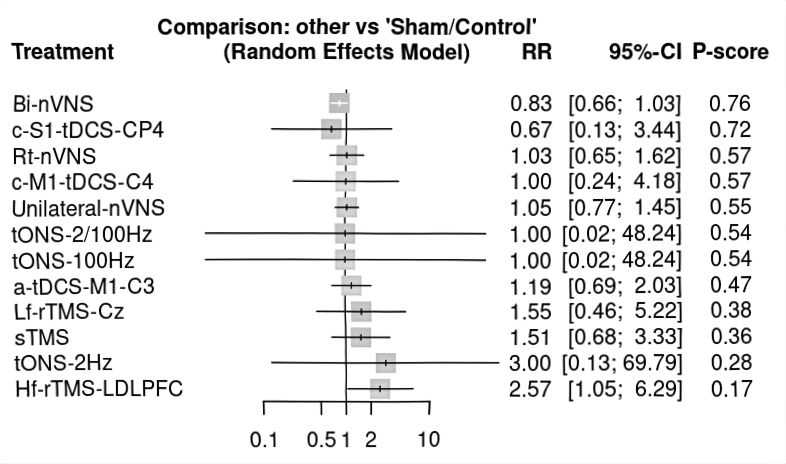


**eFigure 3A-I: SUCRA optimization diagram**

The vertical axis represents the probability for each treatment to be the best option, the best of two options, the best of three options, and so on.

1. **Migraine days**


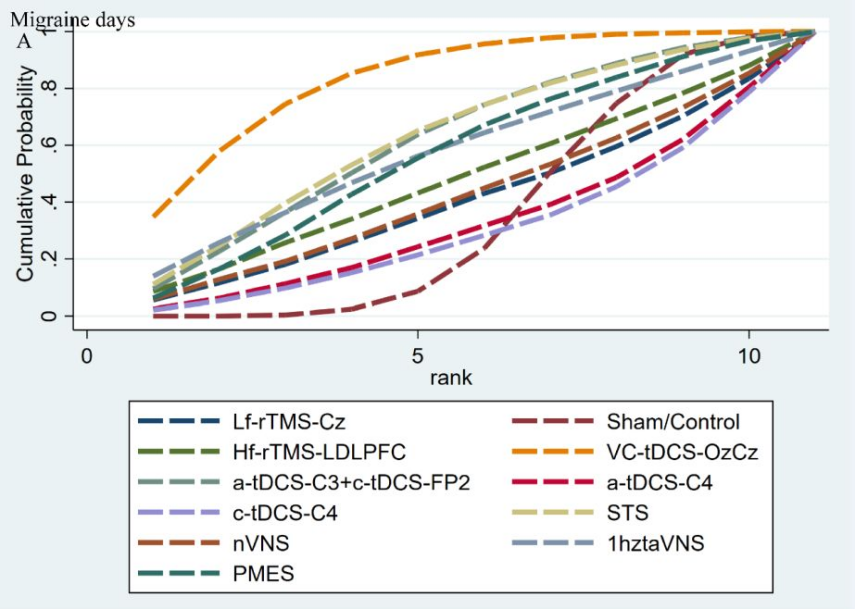


**(B) Attack frequency**

**
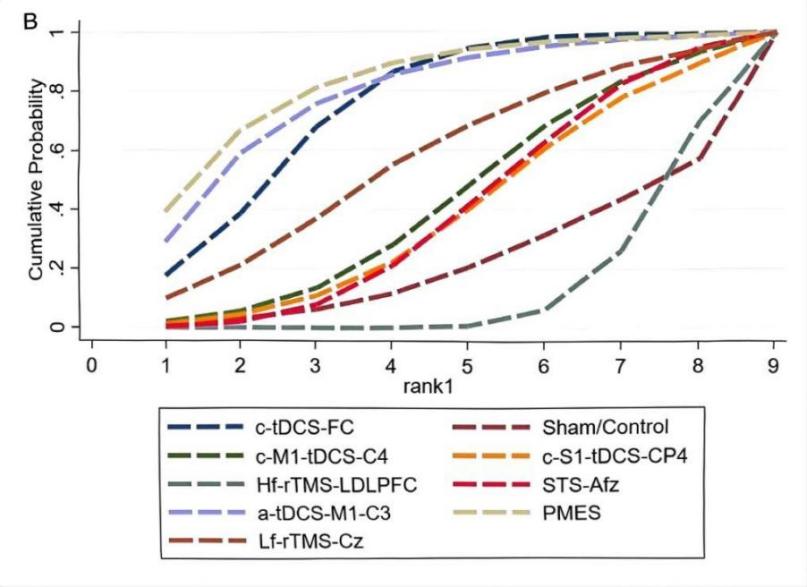
**

**(C) Responder rate**

**
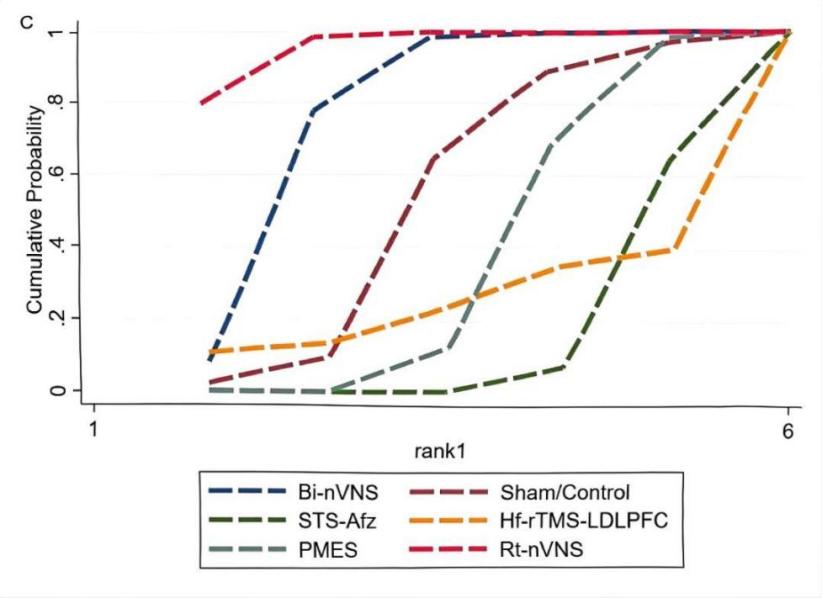
**

**(D) Duration**

**
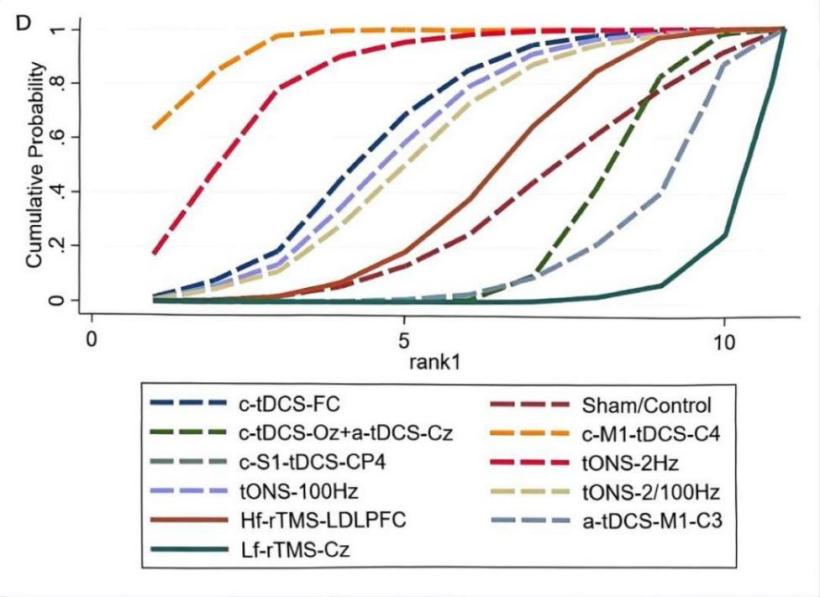
**

**(E)HIT-6**

**
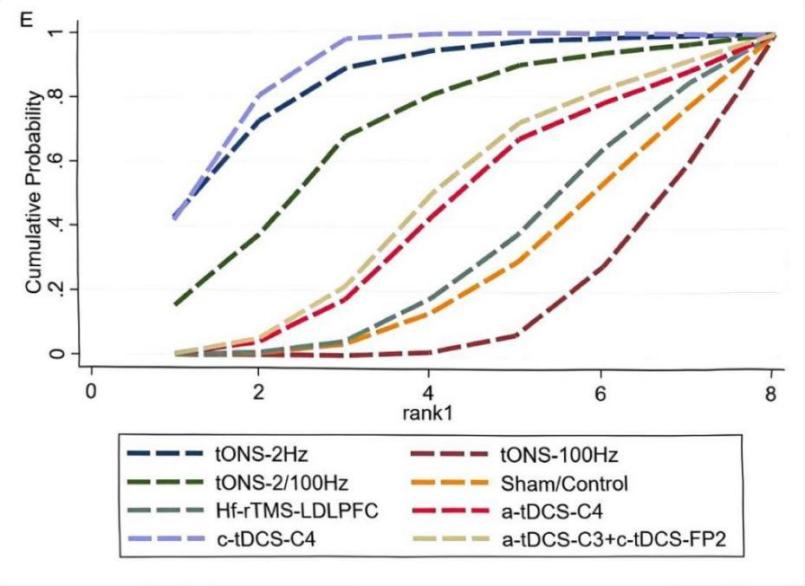
**

**(F) Pain intensity**

**
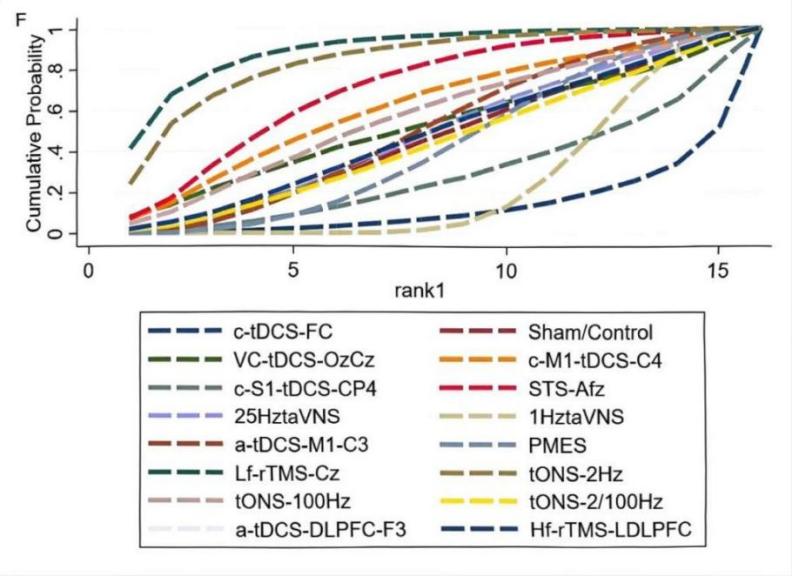
**

**(G) Drop-out rate**

**
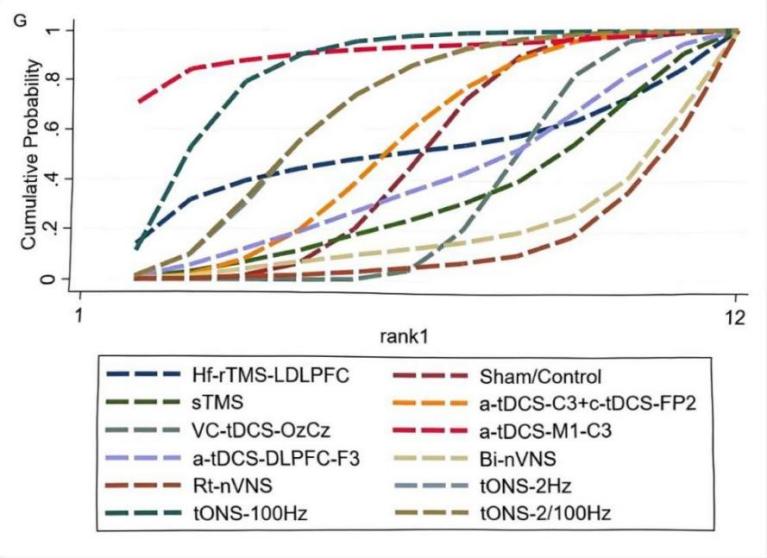
**

**(H) Analgesic**

**
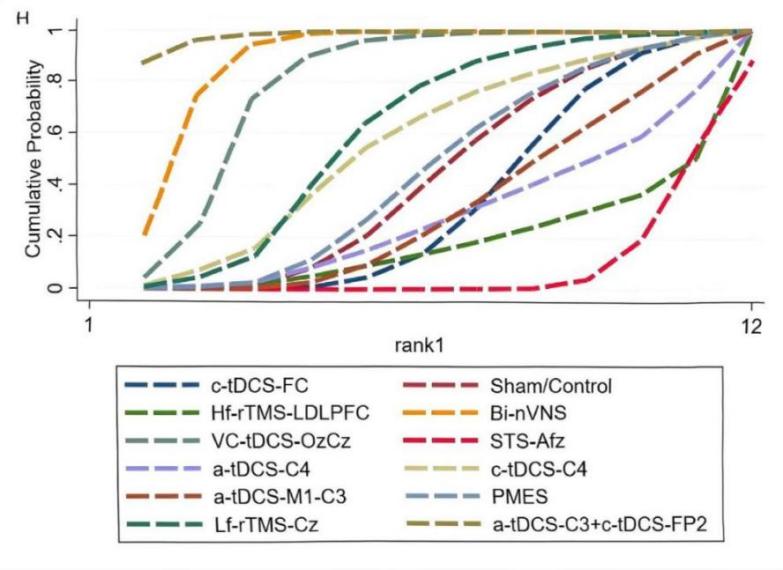
**

**(I)Adverse events**

**
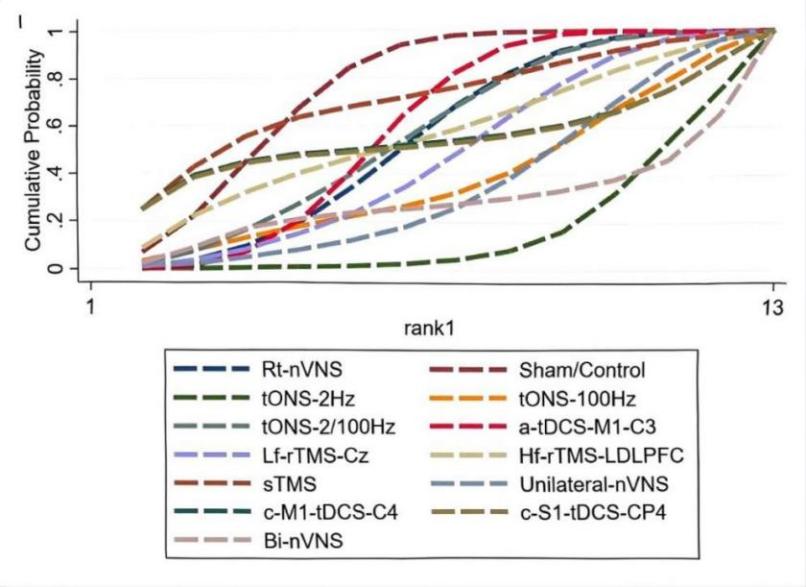
**

**eFigure 4. Funnel plot and Egger/Thompson-Sharp test**

**A.Migraine days**


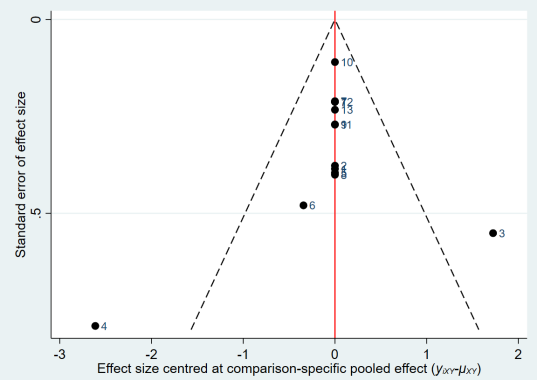


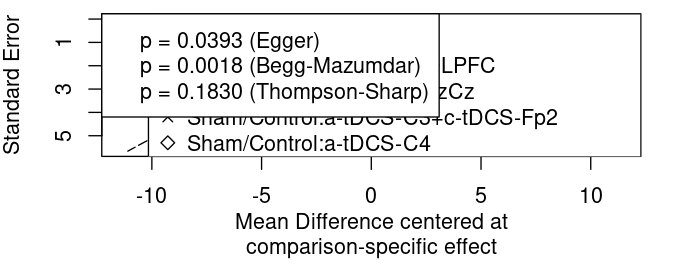


1. **Attack frequency**


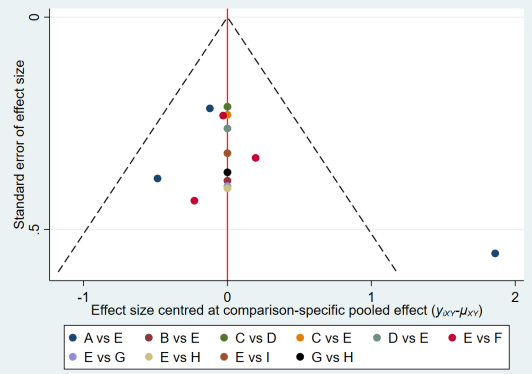


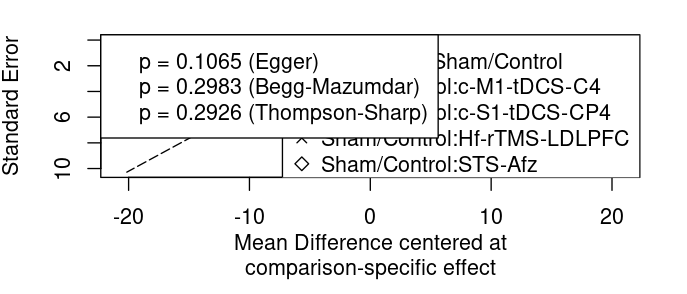


1. **Duration**


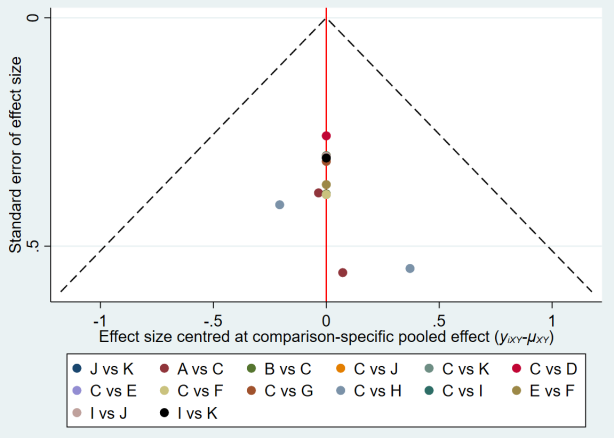


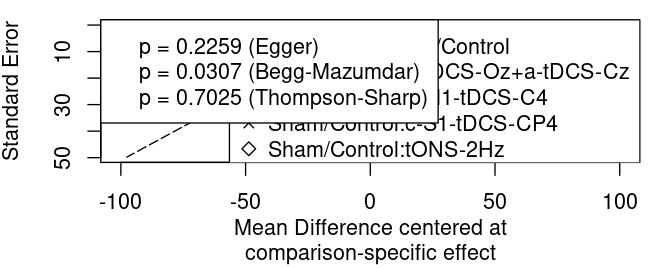


**D.Responder rate**


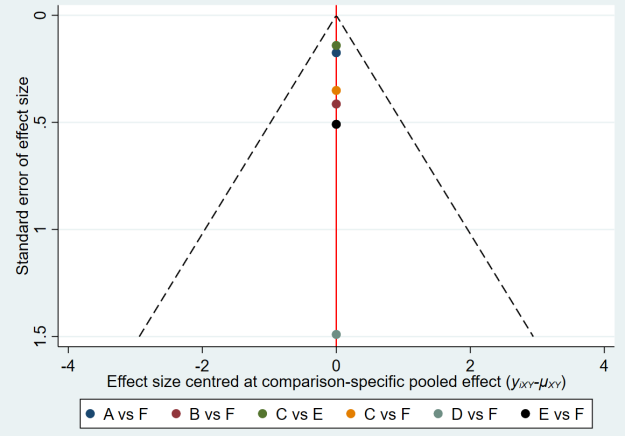


**E.Drop-out rate**


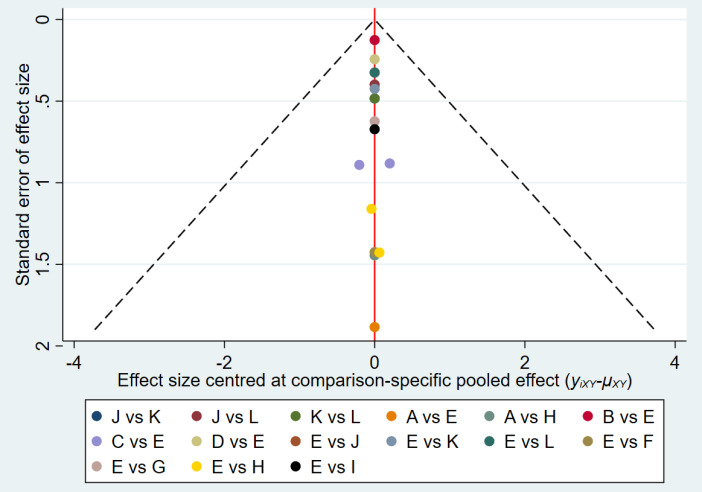


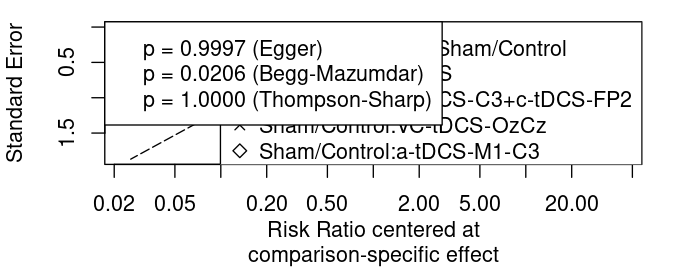


**F.HIT-6**


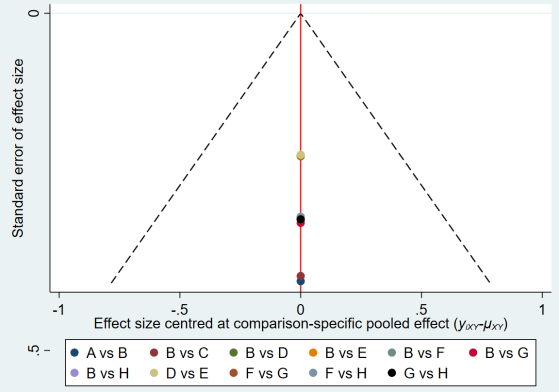


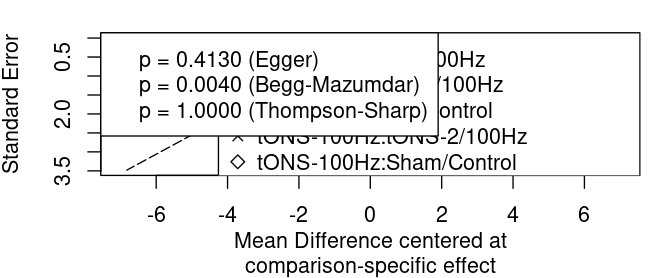


**G.Pain intensity**


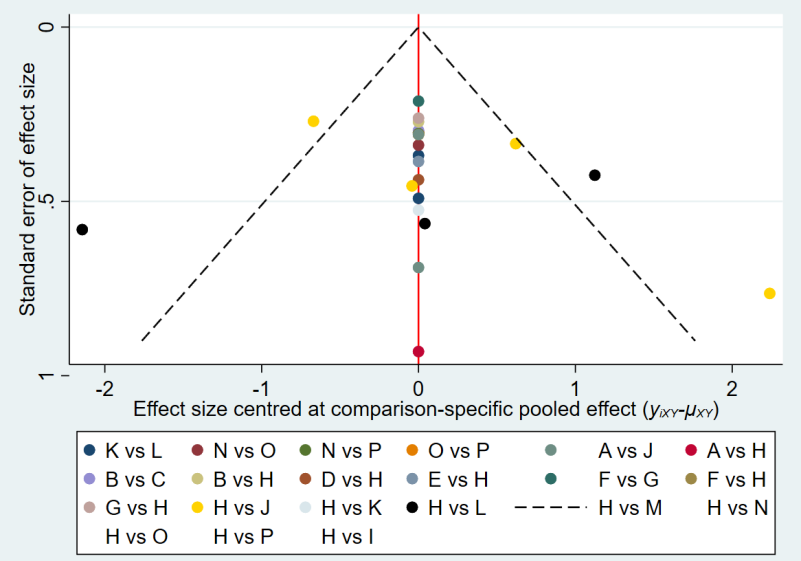


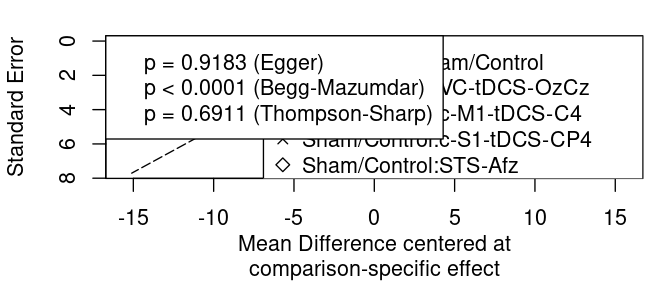


**H.Analgesic**


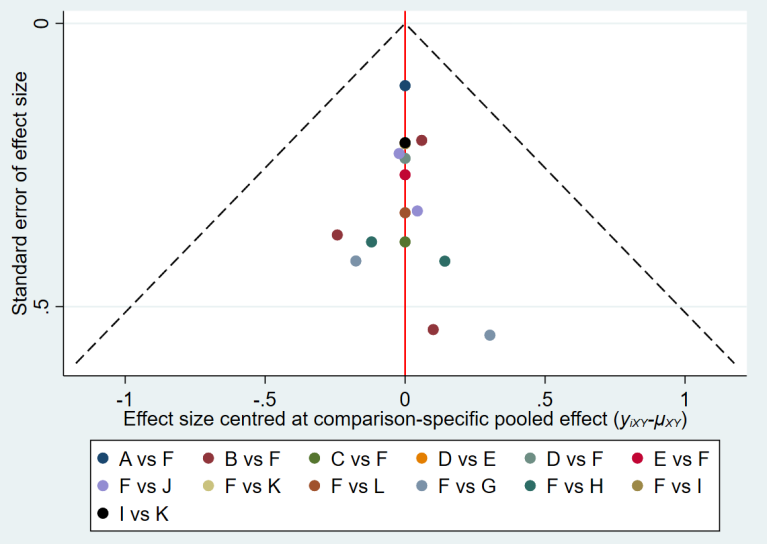


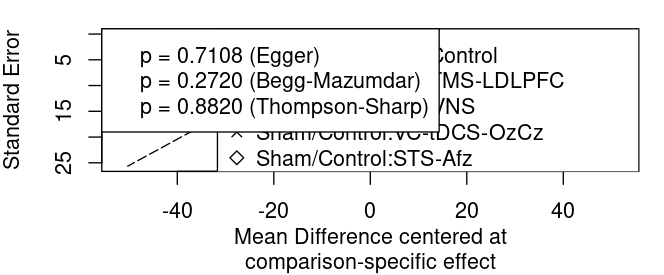


**I.Adverse events**


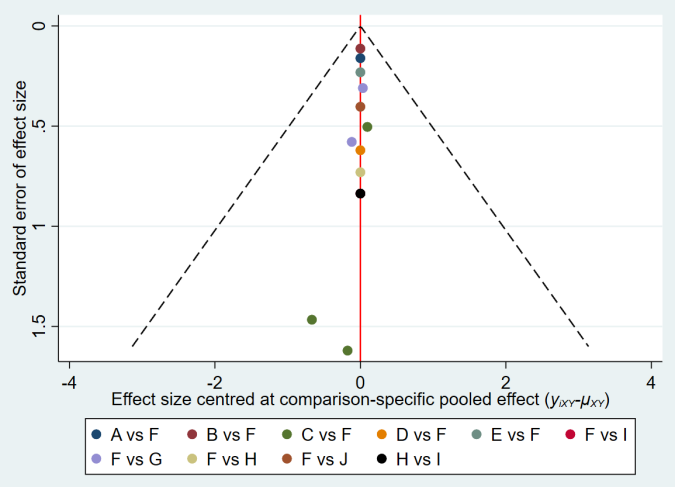

Supplement: Supplementary file 1 — Supplementary Materials: brb371566‐sup‐0001‐SuppMat.docx [file BRB3-16-e71566-s001.docx]
